# Air Quality and Comfort Characterisation within an Electric Vehicle Cabin in Heating and Cooling Operations †

**DOI:** 10.3390/s22020543

**Published:** 2022-01-11

**Authors:** Luigi Russi, Paolo Guidorzi, Beatrice Pulvirenti, Davide Aguiari, Giovanni Pau, Giovanni Semprini

**Affiliations:** 1 Department of Industrial Engineering, Alma Mater Studiorum, University of Bologna, 40136 Bologna, Italy; paolo.guidorzi@unibo.it (P.G.); beatrice.pulvirenti@unibo.it (B.P.); giovanni.semprini@unibo.it (G.S.); 2 Department of Computer Science and Engineering, Alma Mater Studiorum, University of Bologna, 40136 Bologna, Italy; davide.aguiari2@unibo.it (D.A.); giovanni.pau@unibo.it (G.P.); 3 UCLA Samueli Computer Science, University of California, Los Angeles, CA 90024, USA

**Keywords:** electric vehicle, pollutant concentration, HVAC, Arduino sensors, vehicle energetics

## Abstract

This work is aimed at the experimental characterisation of air quality and thermal profile within an electric vehicle cabin, measuring at the same time the HVAC system energy consumption. Pollutant concentrations in the vehicle cabin are measured by means of a low-cost system of sensors. The effects of the HVAC system configuration, such as fresh-air and recirculation mode, on cabin air quality, are discussed. It is shown that the PM concentrations observed in recirculation mode are lower than those in fresh-air mode, while VOC concentrations are generally higher in recirculation than in fresh-air mode. The energy consumption is compared in different configurations of the HVAC system. The novelty of this work is the combined measurement of important comfort parameters such as air temperature distribution and air quality within the vehicle, together with the real time energy consumption of the HVAC system. A wider concept of comfort is enabled, based on the use of low-cost sensors in the automotive field.

## 1. Introduction

One of the major barriers to electric vehicle adoption is due to the limited amount of energy stored in the batteries and needed for traction and auxiliary systems. While ICE vehicles can rely on waste heat for winter requirements, an optimised thermal management of heat loads and gains is crucial for BEVs, paving the way from heat disposal to heat management [1].

Reducing the energy consumption of the HVAC system, as well as other auxiliary systems, is of paramount importance in the era of migration to electric powered transportation; the major challenge being to achieve this while maintaining high levels of comfort inside the cabin. On one hand, the best thermal management of the car’s cabin is obtained by maximising the comfort level along with minimising electrical power demand [2,3,4]. On the other hand, internal air quality (IAQ) related quantities inside the vehicle’s enclosure are affected by outside weather conditions as well as by the heating, ventilation, and air conditioning (HVAC) settings. From this perspective, every improvement of the cabin thermal management must be IAQ aware [5,6,7,8].

As an example, choosing the intake air recirculation mode is recommended under certain conditions to reduce energy use and increase driving range. Provided that energy benefits of the recirculation mode are undisputed, it should also be mentioned that it can produce substantial benefits in relation to certain air contaminants, which are captured by HVAC filters [9], but there are some downsides to consider: CO_2_ and VOC accumulation, pollutant infiltration and odour problems. The United States Environmental Protection Agency (EPA) released fuel credits to manufacturers adopting increased air recirculation in 2017; but cabin air quality, especially when air is recirculating, should be measured and regulated to keep concentrations of certain pollutants below specific thresholds [10,11].

Research in the field of vehicle air quality is leading to new methods of testing and best practices, but still a dedicated standard on performance indicators does not exist. Several efforts have been made to define a standardised test method for interior air quality in the automotive field [12], but this is still an open question. A possible approach to address this issue relies on fractional air recirculation, demonstrating that a compromise is achievable between the benefits of full recirculation and its side effects [13]. Other authors propose approaches based on the use of a signal from environmental prediction services [14], and/or on-board sensors [15] to trigger automatic climate control, even though there is still no clear consensus on how to implement these techniques in the HVAC system’s control strategy. Such an approach would require a trade-off between real time and integral I/O techniques, the former leading to large uncertainties but faster, while the latter provide slower but more stable results [16].

The Joint Research Centre of the European Commission and DG Service Environment are pushing for advancement in this field by stimulating research improvements achieved through the use of low-cost sensors. Although the data measured with these type of sensors are less accurate than laboratory reference equipment, their use has grown greatly in recent years, in applications concerning indoor air quality [17]. Their ease of use, coupled with current scientific advancements [18,19,20], makes them suitable for real-time monitoring applications. The road has therefore been opened for the employment of low-cost sensors in the automotive sector, such as monitoring air quality inside the passenger compartment.

This paper describes a series of experiments on air quality and energy efficiency inside the passenger compartment of an electric vehicle. The measurements were carried out using a portable low-cost sensor system and reading the car’s On-Board Diagnostic bus (OBD) [21]. In this work, the correlation between experimentally measured air quality data and the energy spent by the HVAC system inside the vehicle cabin is investigated. Concentrations of some pollutants in the vehicle cabin are measured by means of a low-cost Arduino sensor-based system. The use of an open-source electronic platform like Arduino allowed fast prototyping and simplified design of the system. In addition, it helped to relax the constraint involved in the construction of hardware and software platforms for data acquisition, following a path that has been outlined by many authors in literature [22,23,24].

The HVAC system configuration regarding fresh-air recirculation mode of the intake air are varied, while PM2.5 and VOC percentages are measured. HVAC air filter performance is evaluated by making cabin air quality measurements with and without the filter installed. The relation between consumed energy, HVAC system settings and pollutant concentrations is obtained, in order to introduce an innovative approach to comfort maintenance inside the car cabin. The novelty of this approach is a win-win perspective, aimed to the concurrent optimisation of the two aspects for BEVs thermal control. The methodology, based on low-cost sensors measurements and applied to a Nissan Leaf Acenta 40 kWh MY2018, is general and applicable to other models of electric car, to show that the use of these sensors for the control of the cabin can yield energy savings together with optimal air quality and comfort levels.

## 2. Materials and Methods

In the following subsections, the integrated experimental setup and the low-cost system of sensors are described in detail.

### 2.1. Description of the Experimental Setup

To characterise the thermal profile and air quality inside the vehicle cabin, two tests have been performed in outdoor parking conditions in the faculty parking lot with the front of the car oriented south.

**Figure 1:**
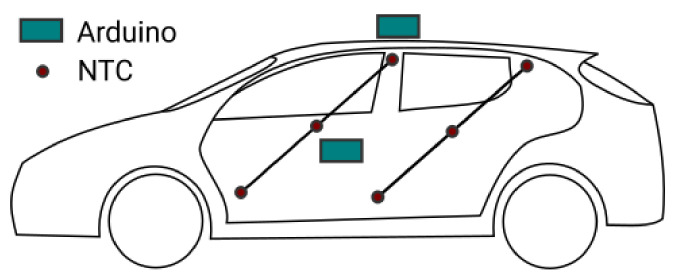
Lateral view of the experimental setup.

The temperature distribution within the vehicle cabin is obtained with a grid of 18 DS18B20 temperature sensors following the approach used in [25,26] to develop a thermal model of a BEV cabin for energy consumption predictions [27]. The cabin has been ideally divided into three slices horizontally: namely the top, middle and bottom levels, as shown in Figure 1 from a lateral view.

On the grid, six sensors are placed for each plane: three in the front side and three in the back side of the cabin. In addition, air quality related quantities have been measured with low-cost sensors on a unique location in the cabin; near the gear shift knob together with the acquisition system.

As external conditions can strongly affect the internal micro-climate [28], a second acquisition system has been placed on the car roof. This is identical to the internal one, except for the presence of a single temperature sensor only. The presence of the second acquisition system is needed to characterise the environment outside the vehicle and to facilitate inside/outside comparisons with data having the same structure and same metrologic fingerprint.

The approach used in the study, conversely from the one used in ISO standards regarding the interior air of road vehicles [29], does not rely on a vehicle test chamber. The latter is well-documented and reliable, but not suitable for real-time operation and low-cost equipment.

To have clearer insights on HVAC capabilities, an on-board diagnostic (OBD) Linux platform was cleverly installed inside the car to directly retrieve and collect different variables from the electronic control units [30]. Specifically, it was the iWave OBD-II: a little device with an ARM Cortex-A7 processor embedded that runs a light Yocto Poky Linux distribution. The iWave OBD-II can upload data via a 4G/LTE CAT4/CAT1 sim modem, geolocate the device with a GPS receiver and it can transmit messages with the Bluetooth Low Energy 4.2 module. Communicating via the OBD-II interface, the board reads the HVAC power consumption, the power used by the auxiliary equipment (e.g., lights, infotainment, rear defroster etc.), and the power used by the heater. A fine-time granularity monitoring of those parameters was necessary to correctly interpret how the cabin air changes throughout the experiment.

The overall measured quantities are: air temperature $t_{a}$, relative humidity RH, air pressure $p_{a}$, TVOC concentration $C_{TVOC}$ and PM_2.5_ concentration $C_{PM}$. The temperature is measured in 18 points as described above, while the other measurements are taken in one point. The same quantities are measured also outside the cabin. Finally, the power usage of the HVAC system is also logged. Table 1 lists the measured quantities and the correspondent accuracy.

Sensor performance is a device dependent issue that can be measured with various qualifiers [31] and ideally addressed individually. In this study, the same approach for all the measured quantities has been used. A sampling time of $T_{s}=10$ s has been adopted. The raw data from the acquisition system has been filtered with a moving mean over a one minute period, this leads to a six-point moving mean. Subsequently, the filtered data has been converted into time-stamped data in tabular form, and eventually re-sampled and synchronised among the three acquisition systems. The data analysis process has been performed using open-source tools, including Python 3.8 and several scientific computing libraries (pandas, matplotlib, numpy and scipy above all) following the exploratory data analysis (EDA) approach provided in [32].

### 2.2. Description of the Arduino-Based System of Sensors

Two independent measurement systems based on Arduino Mega 2560 were built for the measurement of environmental parameters. The systems have an on-board real time clock (RTC), a data logger on flash memory, a fan and a TFT display. The RTC clocks of the two systems are constantly adjusted thanks to time data received from the GPS module. This operation is implemented to facilitate the synchronization of signals from the three acquisition systems.

The internal system is capable of measuring temperatures at 18 locations in the cabin (Maxim Integrated DS18B20 probes). Moreover, the internal system can measure particulate air matter (PM) concentration (Sensirion SPS30 sensor), air TVOC concentration (Sensirion SGP30 sensor), air CO_2_ concentration (Winsen MH-Z19B non-dispersive infrared sensor), concentration formaldehyde (Winsen ZE08 sensor), air temperature, relative humidity and pressure (Bosch BME280 sensor), air flow velocity (hot wire analog sensor) and GPS position, at a unique location. The external system, albeit sharing the same characteristics and using the same sensors, it lacks of the 18-spots temperature measurement, the GPS receiver and the air flow velocity sensor. Both systems are equipped with a fan that conveys air inside the device enclosure, where CO_2_ and formaldehyde sensors are mounted, while the SPS30 sensor is equipped with its built-in fan. Both systems independently sampled data at 10-s intervals. All digital sensors used in the measurement device include a microcontroller that implements optimisation and self-calibration algorithms.

High-precision, easy-to-use DS18B20 sensors were used to measure temperatures inside the cabin in 18 distinct positions; they have a typical accuracy of ±0.5 °C from −10 °C to 85 °C and digitally transmit temperature data on a 1-Wire^®^ bus. The use of 1-Wire protocol [33], together with the unique 64-bit serial code allows many sensors on the same bus, thus reducing the cable length and allowing to uniquely associate a sensor output with its position in the network through a serial-position coupling. Specifically, the DS18B20 actual temperature is provided by a 12-bit analog to digital converter built-in in the digital sensor, with a fine temperature resolution up to 0.0625 °C. Its operating range is between −55 °C to +125 °C.

The BME280 is a high linearity and high accuracy combined temperature, humidity and pressure digital sensor. Its pressure sensing mechanism is resistive, with an operation range of 300 h Pa to 1100 hPa, the temperature sensing principle is of the type diode-voltage with a measurement range of −45 °C to 85 °C, the measurement principle behind humidity is capacitive and its range is 0% to 100% [34]. It features an extremely fast response time $\tau_{63\%}$ of 1 s, thus enabling a consistent oversampling if compared with the current application time granularity of 1 min.

The sensing principle of SPS30 PM sensor is based on laser-scattering, and allows mass concentration and number concentration sensing for particle sizes ranging from 1 μm to 10 μm. As discussed in [20,35], the SPS30 is an optical particle counter (OPC) optimised for PM2.5 and smaller particle analysis. Sensirion PM sensors are indeed calibrated using regularly maintained and aligned high-end reference instruments (e.g., the TSI Optical Particle Sizer Model 3330 or the TSI DustTrak™ DRX 8533) only for 2.5 μm particles size. Moreover, as stated in the sensor specification sheet from the manufacturer, PM4 and PM10 outputs are not directly measured but estimated from smaller particle counts using typical aerosol profiles. A miniaturized fan and a high efficiency particulate air (HEPA) filter are included to reduce the optical part contamination; it also runs its fan at full speed for 10 s every seven days as an automatic cleaning procedure. The mass concentration measurement range is 0 μg/m^3^ to 1000 μg/m^3^.

The SGP30 TVOC sensor is a digital “multi-pixel” gas sensor. It uses multiple sensors, housed on a single metal-oxide gas sensor chip, placed on a thermally controlled hotplate. Digital data output from the sensor includes raw measurements of ethanol and H_2_, and calculated values of TVOC and equivalent CO_2_ via internal algorithm, such as automatic baseline compensation of the measurement [18]. The TVOC data range from this sensor is between 0 to 60,000 ppb. This sensor’s equivalent CO_2_ were disregarded due to its low sensitivity to external pollutants and due to the absence of passengers in parking conditions.

The measurement system has been characterised both in winter and in summer conditions. In the following sections, two typical conditions for winter and summer have been chosen in order to characterise the HVAC system performance in heating and cooling operations, respectively. Winter tests have been performed on 29 January 2021, while summer test have been carried out from 14 July to 15 July 2021.

## 3. Results and Discussion

In this section, the combined measures of air quality and comfort parameters, together with the energy consumption by the electric car are shown and discussed in different seasons, in order to show the differences in relation to the operational mode for the air conditioning. Moreover, an estimation of the filtration performances of the HVAC system is given, by comparing the results corresponding to new and used filters. Ultimately, a detailed treatment of high spatial resolution cabin air temperature profiles is provided in the Supplementary Materials.

### 3.1. Measurements during Heating Operation

Two different test conditions have been investigated for the winter, starting from a state of equilibrium with the external environment, obtained maintaining all systems off and all doors opened for 15 min. Once the equilibrium was reached, the proper test was performed while maintaining the heater on for one hour, and the set-point temperature at its maximum of 30 °C, the fan speed was at its maximum (position 7), and all of the windows and all the doors were closed. During the first test, the recirculation system was off (meaning that the air ventilation system was in the fresh-air configuration), while during the second test the recirculation system was on instead.

#### 3.1.1. Fresh-Air Configuration

**Figure 2:**
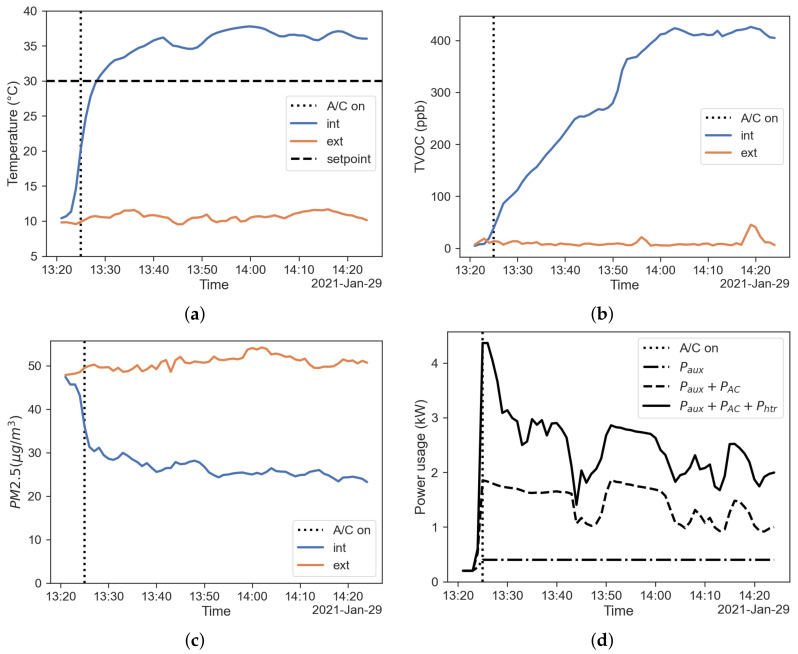
Results regarding the fresh-air mode. (**a**) Temperature inside (blue) and outside (red) the cabin; (**b**) TVOC concentration inside (blue) and outside (red) the cabin; (**c**) PM2.5 concentration inside (blue) and outside (red) the cabin; (**d**) power usage of the HVAC system.

The temperature measured inside and outside the cabin keeping the fresh-air mode is shown in Figure 2a. The air temperature inside the cabin is obtained by the average of the air temperature measurements on the sensors placed on the grid shown in Figure 1, i.e., $t_{int}=t_{avg}$. The TVOC concentration measured inside and outside the cabin in fresh-air mode are shown in Figure 2b. It is shown that the TVOC concentration increases while the HVAC system is working, even in the fresh-air mode. This effect is related to the presence of sources of VOC inside the vehicle cabin and the build up phenomena during the HVAC operation, as expected from the literature [29]. Figure 2c displays the PM2.5 concentration measured inside and outside the cabin in fresh-air mode. The PM concentrations decrease while the HVAC system is working due to the filtering activity of the HVAC filter. This result ties with what is found in the literature [9]. For the case analysed, a filtration efficiency of about η=0.5 for PM2.5 is ascertained. The power usage of the HVAC system, with the contributions of power used by auxiliary equipment, A/C system and PTC heater recorded by the OBD system is shown in the stacked line plot in Figure 2d.

#### 3.1.2. Recirculation Configuration

**Figure 3:**
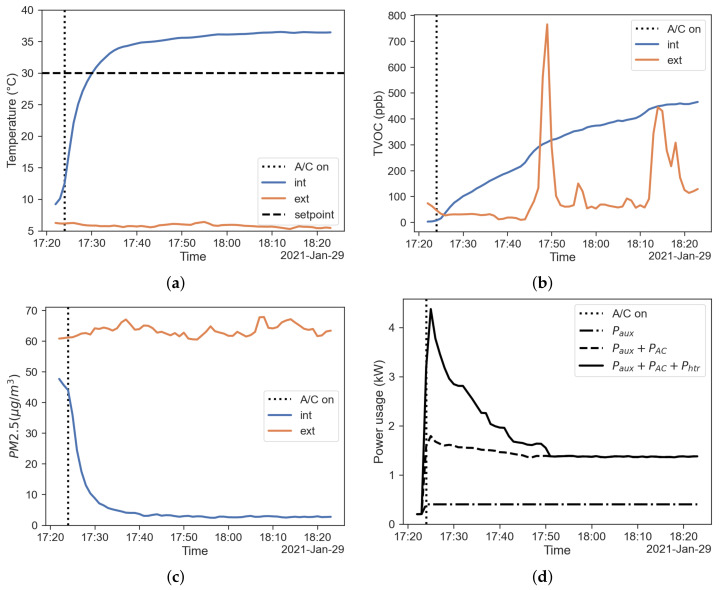
Results regarding the recirculation mode. (**a**) Temperature inside (blue) and outside (red) the cabin; (**b**) TVOC concentration inside (blue) and outside (red) the cabin; (**c**) PM2.5 concentration inside (blue) and outside (red) the cabin; (**d**) power usage of the HVAC system.

Similar considerations can be made for the case with recirculation activated. From the results shown in Figure 3 it is clear that the time to steady state is close to 20 min; again the over-temperature issue remains significant. TVOC concentration reached a value similar to the case without recirculation, but in a longer time with respect to the fresh-air mode. A possible explanation for this behaviour could rely on the fact the source of VOCs inside the cabin is compensated by an improved adsorption performance, as observed by [9]. The PM concentrations decrease to lower values with respect to the fresh-air mode, as shown in Figure 3c. This result shows that the filtration performance is improved by the recirculation mode. Figure 3d shows the power usage of the HVAC system. The figure shows the contributions of auxiliary equipment, the A/C system and the PTC heater to the overall power usage.

#### 3.1.3. Comparison between Fresh-Air and Recirculation Mode in Winter

**Figure 4:**
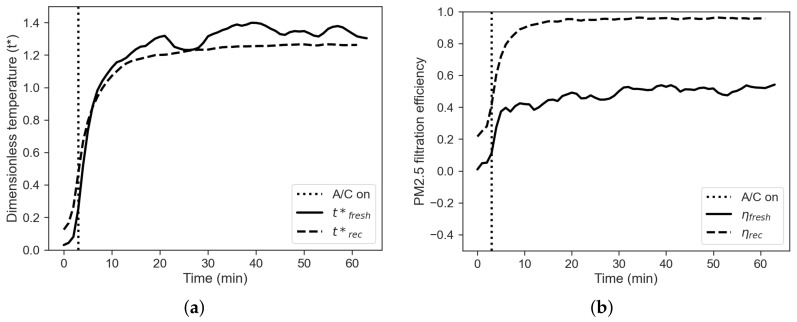
(**a**) Dimensionless temperature profiles, comparison between fresh-air (solid line) and recirculation (dashed line) mode. (**b**) Filtration efficiency, comparison between fresh-air (solid line) and recirculation mode (dashed line).

The open-field tests conducted in this work have been chosen because representative of the real operating conditions of the vehicle. On the other hand, the experiments have been performed with no control on the environment outside the cabin, with repeatability issues. In order to compare the experiments, the following dimensionless temperature is defined:

(1) $$t^{*}=\frac{t_{int}-t_{ext}}{t_{set}-t_{ext}}$$

where $t^{*}$ is the dimensionless temperature, $t_{int}$ is the air temperature measured inside the cabin, $t_{ext}$ is the air temperature measured outside the cabin and $t_{set}$ is the set-point temperature. It is worth to underline that when $t_{int}=t_{ext}$, dimensionless temperature $t^{*}$ is equal to 0, while when $t_{int}=t_{set}$, then $t^{*}$ is equal to 1. These two key points represent two relevant physical states, equilibrium with the external environment and fulfilment of the set-point request, respectively. Figure 4a shows a comparison between the dimensionless temperatures obtained for the two experiments.

It is noticeable that the dimensionless temperature obtained without recirculation is always higher than the one obtained in the case of recirculation mode, thus suggesting that the over-temperature issue is more significant in this case. In addition, the set-point is reached faster during the fresh-air mode than during the recirculation mode.

The filtration efficiency of the vehicle can be defined using a black box approach, where the vehicle cabin is considered as a system with an unknown filtration capacity, while inlet (external) and outlet (internal) concentrations are known. The filtration efficiency is then defined by

(2) $$\eta =1-\frac{C_{int}}{C_{ext}}$$

where $C_{int}$ and $C_{ext}$ are the internal and external concentrations, respectively. Figure 4b shows a comparison between the PM filtration efficiency obtained in the two regimes. The figure shows that PM filtration efficiency with recirculation mode is almost double than the one obtained with the fresh-air mode. It is also noticeable that the filtration efficiency does never reach the ideal value of η=1, suggesting that infiltration rate not equal to zero occur even if the vehicle is parked.

**Figure 5:**
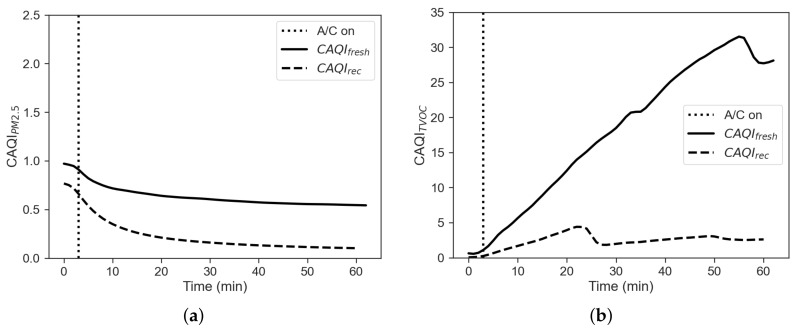
(**a**) CAQI for PM2.5 and (**b**) CAQI for TVOC.

An alternative method to get insights about IAQ of a vehicle cabin relies on a time integrated inside/outside approach proposed in [12]. The associated index, the cabin air quality index (CAQI), is defined as follows:

(3) $$CAQI=\frac{\int_{t_{i}}^{t_{f}}C_{int}\left(t\right)\;dt}{\int_{t_{i}}^{t_{f}}C_{ext}\left(t\right)\;dt}$$

where $C_{int}$ is the internal concentration, $C_{ext}$ is the external concentration, $t_{i}$ is the start time and $t_{f}$ is the stop time. Results based on this index for PM2.5 and TVOC are given in Figure 5.

**Figure 6:**
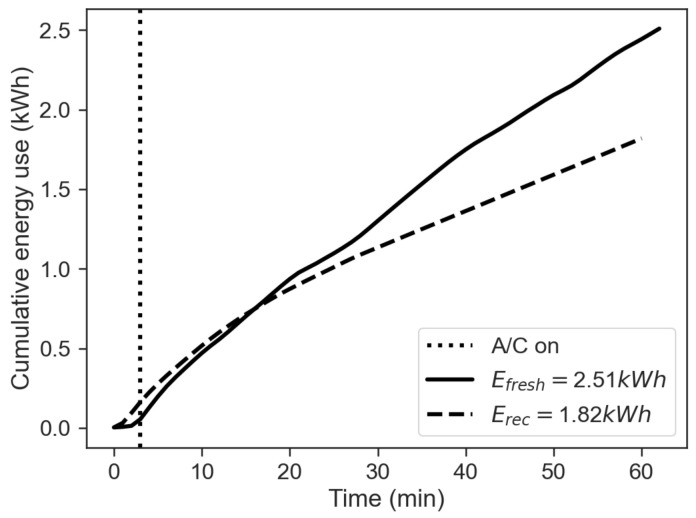
Cumulative energy use, comparison between fresh-air (solid line) and recirculation (dashed line) mode.

The figure shows that the CAQI indexes for PM2.5 and VOC obtained for the fresh-air mode are much greater than the one obtained for the recirculation mode. Figure 6 shows the comparison between the cumulative energy consumption in the two cases of recirculation on and off, calculated as the approximate cumulative integral of $P_{tot}=P_{aux}+P_{AC}+P_{Htr}$ via the trapezoidal method, in order to integrate numeric data rather than a functional expression:

(4) $$E=\int_{t_{i}}^{t_{f}}P_{tot}\left(t\right)\;dt\approx \frac{t_{f}-t_{i}}{2N}\sum_{n=1}^{N}\left(P_{tot}\left(t_{n}\right)+P_{tot}\left(t_{n+1}\right)\right)$$

where $t_{i}$ is the start time, $t_{f}$ the final time and N+1 the number of samples available (equally spaced). The total energy consumption obtained in the recirculation mode is about 3/4 of the value obtained for in fresh-air mode. This result can explain what is shown in Figure 2d and Figure 3d. These figures show that the power usage from the HVAC system is similar for the two modes in the first minutes of operation. However, when the effects of recirculation become prevalent, the values of the HVAC power usage related to the two modes differ considerably. In fact, while $P_{tot}$ peaks at more than 4 kW in the first minutes of operation in both modes, it varies significantly towards the end of the test.

### 3.2. Measurements during Cooling Operation

Two different test conditions have been investigated starting from a state of equilibrium with the external environment, obtained by maintaining all systems off and all doors opened for 15 min. Once the equilibrium was reached, the proper test was performed while maintaining the A/C on for one hour, the set-point temperature at its minimum of 16 °C, the fan speed at its maximum (position 7), and all windows and all doors closed. During the first test, the recirculation system was off (that means that the air ventilation system was in fresh-air configuration), while during the second test the recirculation system was on.

#### 3.2.1. Fresh-Air Configuration

All the experiments confirm that the cooling system is not capable of reaching a quasi-steady state condition in about 60 min, i.e., the temperature reached by the air inside the cabin is far from the set-point temperature value.

**Figure 7:**
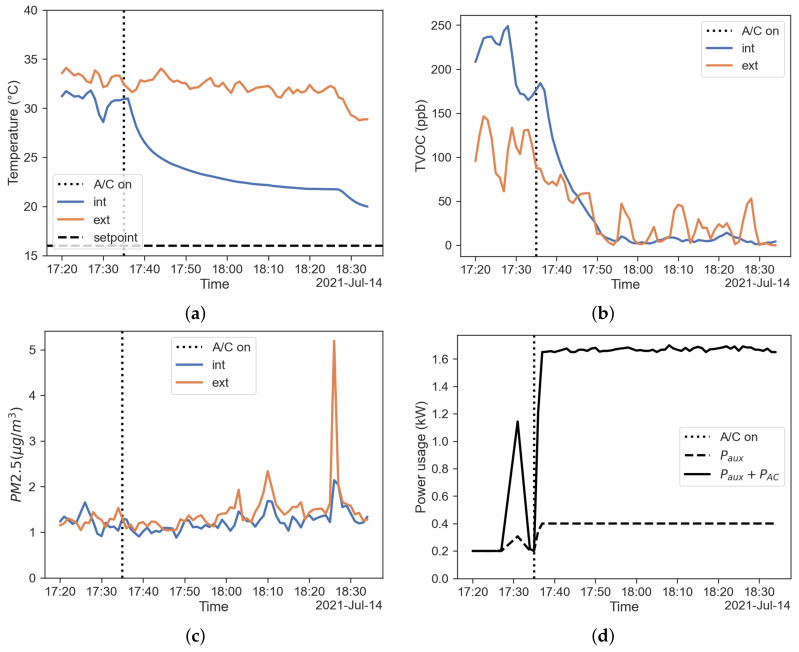
Results regarding the fresh-air mode. (**a**) Temperature inside (blue) and outside (red) the cabin; (**b**) TVOC concentration inside (blue) and outside (red) the cabin; (**c**) PM2.5 concentration inside (blue) and outside (red) the cabin; (**d**) power usage of the HVAC system.

Figure 7a shows the temperature measured inside and outside the cabin keeping the fresh-air mode. Like for the winter operation, the air temperature inside the cabin is calculated as the mean value of the 18 temperature readings for each timestamp.

Figure 7b shows TVOC measured inside and outside the cabin in fresh-air mode. The figure reports that the TVOC concentration is higher than that of the external air at the beginning, but decreases while the HVAC system operates. The lowest concentration is reached, despite the fluctuation on the outside. This behaviour can be explained with a drop of temperature inside the cabin combined with fresh-air mixing, thus reducing the emission from the internal sources.

Figure 7c reports plots of the PM2.5 concentration measured inside and outside the cabin in fresh-air configuration. This result appears to be in contrast with the results shown by [9], i.e., the cabin filter is not able to lower the PM concentration inside the car with a steady state filtration efficiency η= 0 to 0.2. Here we need to consider that the value of PM2.5 concentrations measured were extremely low and under the sensor precision for that particle size range (±10 μ g/m^3^).

Figure 7d shows the power usage of the HVAC system. The figure shows the contributions of power used by auxiliary equipment and A/C system; with PTC heater power being indeed equal to zero in cooling operation.

#### 3.2.2. Recirculation Configuration

**Figure 8:**
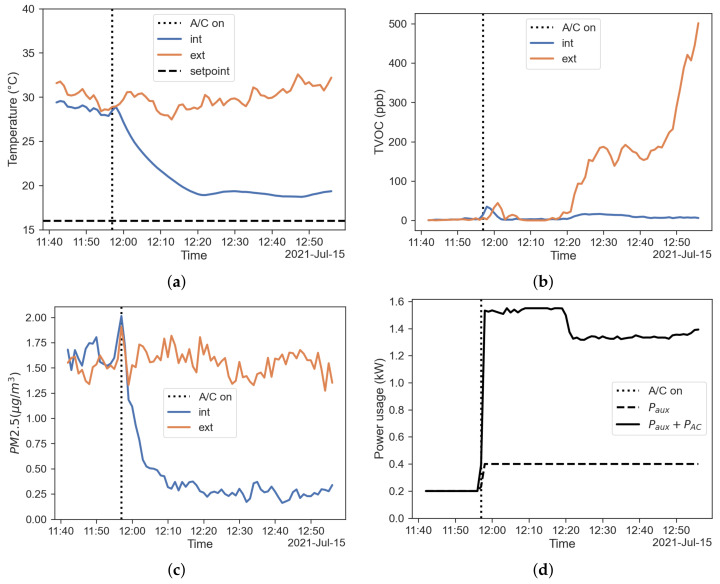
Results regarding the recirculation mode. (**a**) Temperature inside (blue) and outside (red) the cabin; (**d**) TVOC concentration inside (blue) and outside (red) the cabin; (**c**), PM2.5 concentration inside (blue) and outside (red) the cabin; (**d**) power usage of the HVAC system.

As for the previous case with recirculation activated, time to steady state is close to 20 min of operation, while the over-temperature issue is still significant. TVOC concentration shows a different trend. Despite external concentration peaks at the end of the test, the internal one remains quite low. As for PM values, they follow a completely different trend; it is quite clear from Figure 8c how the filtration performance is improved by the recirculation mode, even with absolute values well within the precision range as is the previous test. Figure 8d shows the power usage of the HVAC system, with the contributions of power used by auxiliary equipment and A/C system recorded by the OBD system.

#### 3.2.3. Comparison between Fresh-Air and Recirculation Modes in Summer

**Figure 9:**
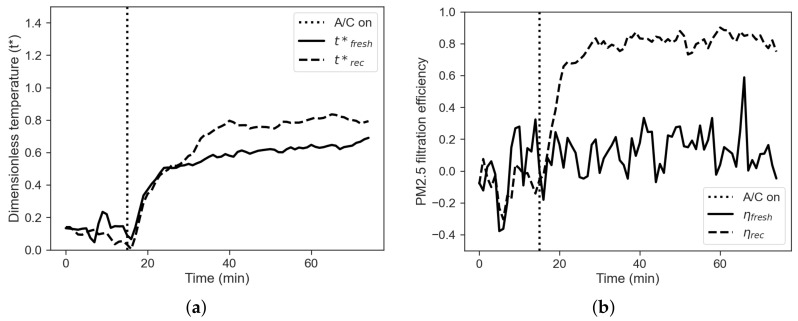
(**a**) Dimensionless temperature profiles, comparison between fresh-air (solid line) and recirculation (dashed line) mode. (**b**) Filtration efficiency, comparison between fresh-air (solid line) and recirculation mode (dashed line).

Experiments in cooling as well as heating mode have been performed in real parking conditions with no control on the environment outside the cabin, repeatability issues are worsen by the increased contribution of solar load in summer. In order to compare the experiments, a temperature adimensionalisation is performed according to Equation (1). Figure 9a shows a comparison between the dimensionless temperatures obtained for the two experiments.

It is noticeable that the value of dimensionless temperature obtained with recirculation is always higher than the one obtained in the fresh-air case, thus suggesting that the cabin approaches better the set-pot in the first case.

**Figure 10:**
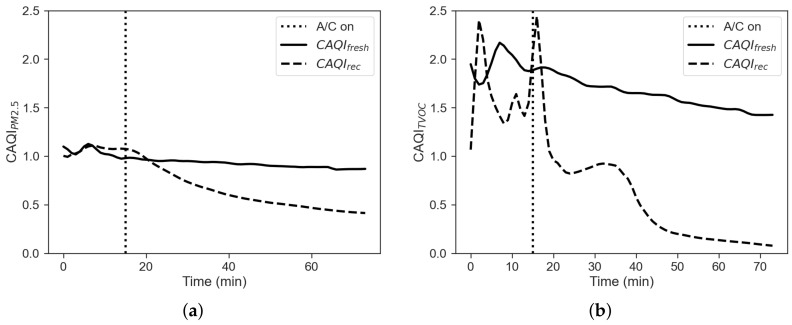
(**a**) CAQI for PM2.5 and (**b**) CAQI for TVOC.

The filtration efficiency of the vehicle can be defined again using a black box approach, where the vehicle cabin is considered as a system with an unknown filtration capacity, while inlet (external) and outlet (internal) concentrations are known, according to Equation (2). Figure 9b shows a comparison between the two cases. The PM filtration efficiency with recirculation mode is well over the one with the fresh-air mode. It is also noticeable that as for the winter case the filtration efficiency never does reach the ideal value of η=1, but it is even lower indeed. This trend can be explained with η being a function of particle size [9], but also of particle concentration itself. As shown for the winter case, another way to investigate cabin performance on airborne pollutants is provided by Equation (3). Figure 10 reports the CAQI trend for PM2.5 and TVOC in fresh-air and recirculation, the latter being less prone to build up of pollutants during operation.

**Figure 11:**
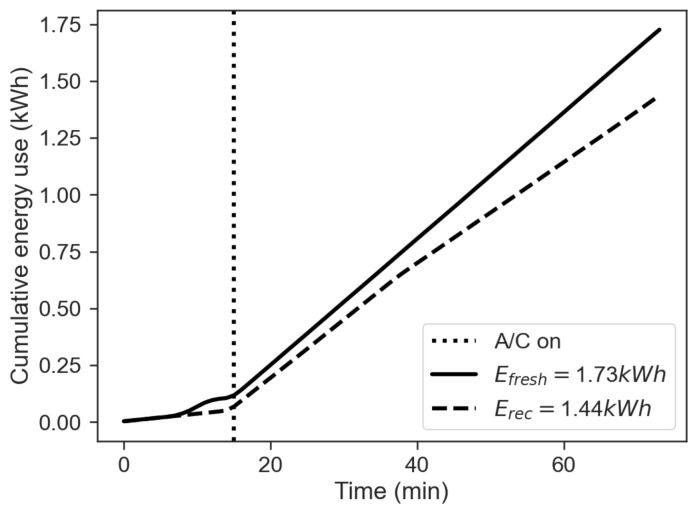
Cumulative energy use, comparison between fresh-air mode (solid line) and recirculation (dashed line) mode.

Figure 11 shows the comparison between the cumulative energy consumption in the two cases of recirculation on and off, calculated as the approximate cumulative integral of $P_{tot}=P_{aux}+P_{AC}$ via the trapezoidal method as done in the winter case with Equation (4). The total energy consumption obtained in the recirculation mode on is about 4/5 of the value obtained for the fresh-air mode. This result can explain what is shown in Figure 7d and Figure 8d. These figures show that the power usage from the HVAC system is similar for the two modes in the first minutes of operation. However, when the effects of recirculation become prevalent, the HVAC power usage related to this mode decreases after about 20 min, even though the difference is less prominent than in winter operation.

### 3.3. Effect of Filter Conditions on Filtration Performance

**Figure 12:**
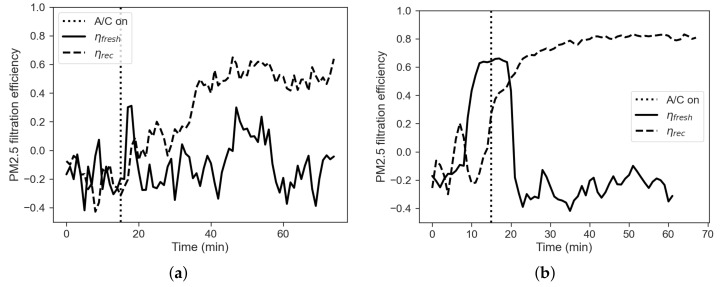
Filtration efficiency, comparison between fresh-air mode (solid line) and recirculation (dashed line) mode, with (**a**) no cabin filter installed and (**b**) with brand new cabin filter installed.

In this section we report some results regarding the filtration performances of the Leaf cabin filter (Figure 12). In detail we performed two tests in summer operating conditions manipulating the filter. First, we performed the experiment without the filter. Then, a second experiment was performed after installing a brand-new filter. Figure 12a shows that the presence of the filter has an effect of filtration efficiency, lowering its value from 80% to 60%. The figure shows that even without a filter the recirculation mode provides a sort of filtration. This result suggests that part of the filtration is made by the filter and part is given by other devices in the HVAC system, i.e., a fraction of the pollutants is captured by the evaporator fins, or by the ducts between the cabin and the evaporator. Curves for fresh-air mode show filtration efficiency around zero for both the cases (no filter and with a filter), with more fluctuations for the case without a filter. Then, the presence of the filter does not improve the air quality within the cabin both by using a filter and by not using it.

## 4. Conclusions

In this paper, a characterisation of the air quality within the cabin of a battery electric vehicle (BEV), together with real time measurements of HVAC system energy consumption has been presented. The temperature, PM, and VOC concentrations have been measured by means of a low-cost Arduino-based system of sensors. Comparisons between the air quality obtained in the cabin during different configuration modes of the air-ventilation system have been carried out.

The results show that, while PMs are filtered, VOCs concentrations increase during operation in recirculation mode. At the same time, the HVAC energy consumption in recirculation mode is about 70% of the energy consumption measured in fresh-air mode during heating operation. In the cooling operation, the HVAC energy consumption in recirculation mode is about 80% of the energy consumption measured in fresh-air mode.

Recirculation mode is found to be the best choice for BEVs, both for reducing some pollutants concentrations and for saving energy. The use of a new filter can improve the filtration efficiency in recirculation mode.

The methodology presented in this paper, applied to a Nissan Leaf Acenta 40 kWh, can be easily extended to other vehicles. This approach is very important for BEVs, as the parameters analysed are crucial for these vehicles. In fact, the air quality is strongly related to air-circulation modes, such as the fresh-air or recirculation modes. The recirculation mode should be chosen for energy saving in order to extend the BEV drive range, but a fresh-air mode is needed in some cases to ensure low concentrations of pollutants within the cabin. Control systems should consider these results in order to manage the HVAC system operation in a win-win approach for BEVs. 

## Figures and Tables

**Table 1:** Measured quantities.

Variable (Unit)	Sensors Specifications			
	Description	Manufacturer	Model	Accuracy (Offset + Gain)
*t_a_*(°C)	Air temp.	Maxim Integrated	DS18B20	±(0.5 °C + 1% mv ${}^{a}$)
RH(%)	Air rel. hum.	Bosch	BME280	±(3%RH+1%RH)
$p_{a}\left(h\mathrm{Pa}\right)$	Air pres.	Bosch	BME280	±(1.5 hPa+0.12 hPa)
TVOC(ppb)	TVOC conc.	Sensirion	SGP30	±(15% mv)
PM (μg/m${}^{3}$)	PM2.5 conc.	Sensirion	SPS30	±(10 μg/m${}^{3}$ + 10% mv)
$P_{i}\left(kW\right)$	Subsystem i power usage	iWave	OBD/Linux	±(250 W)

^a^ mv = measured value.

## Data Availability

Not Applicable.

## Supplementary Materials

The following are available online at https://www.mdpi.com/article/10.3390/s22020543/s1, Figure S1: Heating temperature profiles, fresh-air mode, Figure S2: Heating temperature difference profiles, fresh-air mode, Figure S3: Heating temperature profiles, recirculation mode, Figure S4: Heating temperature difference profiles, recirculation mode, Figure S5: Cooling temperature profiles, fresh-air mode, Figure S6: Cooling temperature difference profiles, fresh-air mode, Figure S7: Cooling temperature profiles, recirculation mode, Figure S8: Cooling temperature difference profiles, recirculation mode.

## Abbreviations

The following abbreviations are used in this manuscript:

BEV	Battery Electric Vehicle
HVAC	Heating, Ventilation and Air Conditioning
A/C	Air Conditioning
PTC	Positive Temperature Coefficient
IAQ	Internal Air Quality
OBD	On-Board Diagnostic
MY	Model Year
I/O	Inside/Outside
NTC	Negative Temperature Coefficient
PM	Particulate Matter
VOC	Volatile Organic Compounds
