# Supplementary material for: Air Quality and Comfort Characterisation within an Electric Vehicle Cabin in Heating and Cooling Operations
Source: Sensors (Basel). 2022 Jan 11;22(2):543. doi: 10.3390/s22020543 (PMC8778250; doi:10.3390/s22020543)
Supplement: Supplementary file 1 [file sensors-22-00543-s001.zip › sensors-1450505 -supp -for pub-final/combined_comparison_20211224.pdf]

Draftable

Draftable Comparison Export

This document is an exported comparison with limited functionality, generated by Draftable Desktop. To access full functionality, use Draftable's powerful comparison viewer in any of our products.

**Left document:**	metroatomotive_VIAQ___Special_Issue_on_MDPI_Sensors___major_revisions.pdf
**Right document:**	Air_Quality_and_Comfort_Characterisation_within_an_Electric_Vehicle_Cabin_in_Heating_and_Cooling_Operations___2nd_round.pdf

What is this document?

This is a comparison of two documents. The two documents are interleaved such that the left document is displayed on even pages and the right document is displayed on odd pages.

Is there a specific way I should view this file?

This document is intended to be viewed in Two Page Continuous mode (or sometimes called 'Two Page Scrolling'). It should open in this mode by default when using Adobe Acrobat and most popular PDF readers.

If the document opens in a different view, you can often change this in the settings. In Adobe Acrobat, go to **View > Page Display > Two Page Scrolling**.

Why are there blank pages?

Blank pages are inserted to keep both documents as aligned as much as possible.

How do I read the changes?

Text deleted from the left document and, hence, not in right document is highlighted red. Text added to the right document and, hence, not in left document is highlighted green.

Tip for printing

When printing this document, we recommend printing double-sided and include this first page. This will result in the matching text being displayed on different pages and easily readable, much like a book.

For more information

Draftable offers powerful document comparison solutions for all use-cases. To view our products, please visit our website: draftable.com.

Article

Characterisation of Comfort and Air Quality in an Electric Vehicle in Heating and Cooling Operations†

Luigi Russi [1,‡,*], Paolo Guidorzi [1], Beatrice Pulvirenti [1], Davide Aguiari [2], Giovanni Pau[21] and Giovanni Semprini [1]

[1] Department of Industrial Engineering, Alma Mater Studiorum, University of Bologna; luigi.russi@unibo.it
[2] Department of Computer Science and Engineering, Alma Mater Studiorum, University of Bologna; davide.aguiari2@unibo.it
* Correspondence: luigi.russi@unibo.it
† This paper is an extended version of [1]
‡ Current address: Viale del Risorgimento 2, 40136, Bologna, Italy

Abstract: This work is aimed at the experimental characterisation of air quality within a vehicle cabin, by measuring at the same time the HVAC system energy consumption. Pollutant concentrations in the vehicle cabin are measured by means of a low-cost system of sensors. The effects of the HVAC system configuration on the cabin air quality are discussed, such as fresh-air and recirculation mode. It is shown that the PM concentrations obtained in the recirculation mode are lower than those obtained in fresh-air mode, while the VOC concentrations obtained in the recirculation mode are higher than those obtained in fresh-air mode. The energy consumption is measured in relation to the configuration of the HVAC system and the measured concentrations. The novelty of this work is the combined measurement of important comfort parameters such as temperature distribution and air quality within the vehicle and the real time energy consumption of the HVAC system. A wider concept of comfort is enabled, based on the use of low-cost sensors in the automotive field.

Keywords: Electric vehicle, pollutant concentration, HVAC, Arduino sensors, vehicle energetics

Citation: Title. *Sensors* **2021**, *1*, 0. https://doi.org/

Received:
Accepted:
Published:

Publisher's Note: MDPI stays neutral with regard to jurisdictional claims in published maps and institutional affiliations.

Copyright: © 2021 by the authors. Submitted to *Sensors* for possible open access publication under the terms and conditions of the Creative Commons Attribution (CC BY) license (https://creativecommons.org/licenses/by/ 4.0/).

1. Introduction

This paper describes a series of experimental readings of air quality and energy efficiency inside the passenger compartment of a car. The measurements were carried out using low cost sensor systems and connecting to the car's On-Board Diagnostic bus (OBD) [1]. The temperature and the other environmental parameters inside the vehicle's enclosure are affected by outside weather conditions as well as by the Heating, Ventilation, and Air Conditioning (HVAC) working state. Reducing the power consumption of the HVAC system, as well as other secondary systems of course, is of paramount importance in this era of migration to electric powered transportation, and the major challenge is to achieve this while maintaining high levels of comfort in the cabin. The best thermal management in the cabin of the car is obtained by maximizing comfort level along with minimizing electrical power demand. For example, choosing the intake air recirculation mode is recommended under certain conditions to economize energy and increase driving duration. EPA released fuel credits with the growth of re-circulation of air in 2017. The energy benefits of the recirculation mode are undisputed, but there are some points to consider: CO_2 and VOC accumulation, pollutant infiltration and odour problems. Cabin air quality, especially when air is re-circulating, should be measured and regulated to keep concentrations of certain pollutants below specific thresholds [2]. It should also be mentioned that air re-circulation can produce substantial benefits in relation to certain air contaminants, which are captured by HVAC filters. Research in the field of vehicle air quality is leading to new methods of testing and best practices, but

Article

Air Quality and Comfort Characterisation within an Electric Vehicle Cabin in Heating and Cooling Operations†

Luigi Russi [1,‡,*], Paolo Guidorzi [1], Beatrice Pulvirenti [1], Davide Aguiari [2], Giovanni Pau[21] and Giovanni Semprini [1]

[1] Department of Industrial Engineering, Alma Mater Studiorum, University of Bologna; luigi.russi@unibo.it
[2] Department of Computer Science and Engineering, Alma Mater Studiorum, University of Bologna; davide.aguiari2@unibo.it
* Correspondence: luigi.russi@unibo.it

† This paper is an extended version of [1]
‡ Current address: Viale del Risorgimento 2, 40136, Bologna, Italy

Abstract: This work is aimed at the experimental characterisation of air quality and thermal profile within an electric vehicle cabin, measuring at the same time the HVAC system energy consumption. Pollutant concentrations in the vehicle cabin are measured by means of a low-cost system of sensors. The effects of the HVAC system configuration, such as fresh-air and recirculation mode, on cabin air quality are discussed. It is shown that the PM concentrations observed in recirculation mode are lower than those in fresh-air mode, while VOC concentrations are generally higher in recirculation than in fresh-air mode. The energy consumption is compared in different configurations of the HVAC system. The novelty of this work is the combined measurement of important comfort parameters such as air temperature distribution and air quality within the vehicle, together with the real time energy consumption of the HVAC system. A wider concept of comfort is enabled, based on the use of low-cost sensors in the automotive field.

Keywords: Electric vehicle, pollutant concentration, HVAC, Arduino sensors, vehicle energetics

1. Introduction

One of the major barriers to electric vehicle adoption is due to the limited amount of energy stored in the batteries and needed for traction and auxiliary systems. While ICE vehicles can rely on waste heat for winter requirements, an optimised thermal management of heat loads and gains is crucial for BEVs, paving the way from heat disposal to heat management [2].

Reducing the energy consumption of the HVAC system, as well as other auxiliary systems is of paramount importance in the era of migration to electric powered transportation; the major challenge being to achieve this while maintaining high levels of comfort inside the cabin. On one hand, the best thermal management of the car's cabin is obtained by maximising comfort level along with minimising electrical power demand [3–5]. On the other hand, internal air quality (IAQ) related quantities inside the vehicle's enclosure are affected by outside weather conditions as well as by the Heating, Ventilation, and Air Conditioning (HVAC) settings. From this perspective, every improvement of the cabin thermal management must be IAQ aware [6–9].

As an example, choosing the intake air recirculation mode is recommended under certain conditions to reduce energy use and increase driving range. Provided that energy benefits of the recirculation mode are undisputed, it should also be mentioned that it can produce substantial benefits in relation to certain air contaminants, which are captured by HVAC filters [10], but there are some downsides to consider: CO_2 and VOC accumulation, pollutant infiltration and odour problems. United States Environmental Protection Agency (EPA) released fuel credits to manufacturers adopting increased air

Citation: Title. *Sensors* **2021**, *1*, 0. https://doi.org/

Received:
Accepted:
Published:

Publisher's Note: MDPI stays neutral with regard to jurisdictional claims in published maps and institutional affiliations.

Copyright: © 2021 by the authors. Submitted to *Sensors* for possible open access publication under the terms and conditions of the Creative Commons Attribution (CC BY) license (https://creativecommons.org/licenses/by/ 4.0/).

still a standard on performance indicators does not exist. Several efforts have been made in order to define a standardized test method for automotive interior air quality [3], but this is still an open question. The Joint Research Center of the European Commission and DG Service Environment are pushing for advancement in this field by stimulating research improvements achieved through the use of low-cost sensors. Although the data measured with these types of sensors are less accurate than laboratory reference equipment, their use has grown greatly in recent years, in applications concerning indoor air quality (IAQ) [4]. This is due also to their ease of use, which makes them suitable for real-time monitoring applications. The road has therefore been opened for the use of low-cost sensors in the automotive sector, for monitoring the air quality inside the passenger compartment. In this paper, the correlation between experimentally measured air quality data and the energy spent by the HVAC system inside the vehicle cabin is investigated. Concentrations of some pollutants in the vehicle cabin [5] are measured by means of a low-cost Arduino sensor-based system [6]. The HVAC system configuration and fresh air recirculation mode of the intake air are varied while PM2.5 and VOC percentages are measured. HVAC air filter performance is evaluated by making cabin air quality measurements with and without the filter installed. The relation between consumed energy, HVAC system working state and pollutant concentrations is obtained in order to investigate a new approach to comfort maintenance inside the car cabin, and for optimizing the development of BEVs vehicles methodologies.

2. Materials and Methods

In the following subsections, the integrated experimental setup and the low-cost sensors chosen are described.

2.1. Description of the experimental setup

To characterise the thermal profile and air quality inside the vehicle cabin, two tests have been performed in real outdoor parking conditions. The vehicle was a 2018 Nissan Leaf Acenta 40 kWh, parked in the faculty parking lot with the front of the car oriented south.

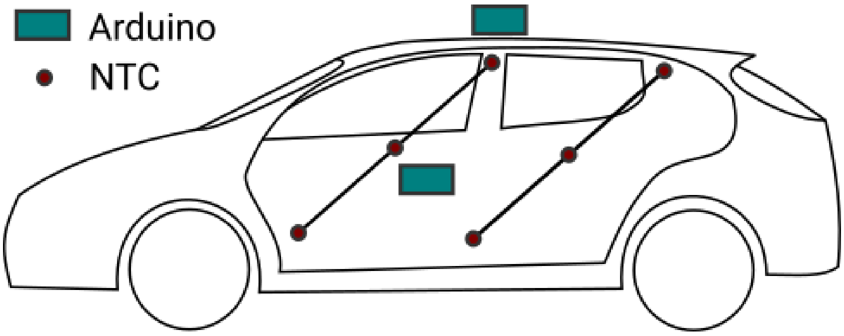

Figure 1. Lateral view of the experimental setup

The temperature distribution within the vehicle cabin is obtained with a grid of 18 DS18B20 temperature sensors following the approach used in [7] and [8] to develop a thermal model of a BEV cabin for energy consumption predictions [9]. The cabin has been ideally divided into three slices horizontally, namely top, middle and bottom level, as shown in Fig. 1 from a lateral view.

On the grid, six sensors are placed on each plane: three in the front side and three in the back side of the cabin. In addition, air quality related quantities have been measured with low-cost sensors on a single location in the cabin; near the gear shift knob together with the acquisition system.

As external conditions can strongly affect the internal micro-climate [10], a second acquisition system has been placed on the car roof. This is identical to the internal one, except for the presence of a single temperature sensor only. The presence of the second

recirculation in 2017; but cabin air quality, especially when air is recirculating, should be measured and regulated to keep concentrations of certain pollutants below specific thresholds [11,12].

Research in the field of vehicle air quality is leading to new methods of testing and best practices, but still a dedicated standard on performance indicators does not exist. Several efforts have been made to define a standardised test method for interior air quality in the automotive field [13], but this is still an open question. A possible approach to address this issue relies on fractional air recirculation, demonstrating that a compromise is achievable between the benefits of full recirculation and its side effects [14]. Other authors propose approaches based on the use of a signal from environmental prediction services [15], and/or on-board sensors [16] to trigger automatic climate control, even though there is still no clear consensus on how to implement these techniques in the HVAC system's control strategy. Such approach would require a trade-off between real time and integral I/O techniques, the former leading to large uncertainties but faster, while the latter provide slower but more stable results [17].

The Joint Research Centre of the European Commission and DG Service Environment are pushing for advancement in this field by stimulating research improvements achieved through the use of low-cost sensors. Although the data measured with these type of sensors are less accurate than laboratory reference equipment, their use has grown greatly in recent years, in applications concerning indoor air quality [18]. Their ease of use, coupled with current scientific advancements [19–21], makes them suitable for real-time monitoring applications. The road has therefore been opened for the employment of low-cost sensors in the automotive sector, such as monitoring air quality inside the passenger compartment.

This paper describes a series of experiments on air quality and energy efficiency inside the passenger compartment of an electric vehicle. The measurements were carried out using a portable low-cost sensor system and reading the car's On-Board Diagnostic bus (OBD) [1]. In this work, the correlation between experimentally measured air quality data and the energy spent by the HVAC system inside the vehicle cabin is investigated. Concentrations of some pollutants in the vehicle cabin are measured by means of a low-cost Arduino sensor-based system. The use of an open-source electronic platform like Arduino allowed fast prototyping and simplified design of the system. In addition, it helped to relax the constraint involved in the construction of hardware and software platforms for data acquisition, following a path that has been outlined by many authors in literature [22–24].

The HVAC system configuration regarding fresh-air recirculation mode of the intake air are varied while PM2.5 and VOC percentages are measured. HVAC air filter performance is evaluated by making cabin air quality measurements with and without the filter installed. The relation between consumed energy, HVAC system settings and pollutant concentrations is obtained, in order to introduce an innovative approach to comfort maintenance inside the car cabin. The novelty of this approach is a win-win perspective, aimed to the concurrent optimisation of the two aspects for BEVs thermal control. The methodology, based on low-cost sensors measurements and applied to a Nissan Leaf Acenta 40 kWh MY2018, is general and applicable to other models of electric car, to show that the use of these sensors for the control of the cabin can yield energy saving together with an optimal air quality and comfort levels.

2. Materials and Methods

In the following subsections, the integrated experimental setup and the low-cost system of sensors are described in detail.

acquisition system is needed to characterize the environment outside the vehicle and to facilitate inside/outside comparisons with data having the same structure and same metrologic fingerprint.

The approach used in the study, conversely from the one used in ISO standards regarding interior air of road vehicles[11], does not rely on a vehicle test chamber. The latter being well documented and reliable, but not suitable for real-time operation and low-cost equipment.

To have clearer insights on HVAC capabilities, an On-Board Diagnostic (OBD) Linux platform was cleverly installed inside the car to directly retrieve and collect different variables from the electronic control units [12]. Specifically, it was the *iWave OBD-II*: a little device with an *ARM Cortex-A7* processor embedded that runs a light *Yocto Poky* Linux distribution. The *iWave OBD-II* can upload data via a 4G/LTE CAT4/CAT1 sim modem, geolocate the device with a GPS receiver and it can transmit messages with the Bluetooth Low Energy 4.2 module. Communicating via the OBD-II interface, the board reads the HVAC power consumption, the power used by the auxiliary equipment (e.g. lights, infotainment, rear defroster etc.), and the power used by the heater. A fine-time granularity monitoring of those parameters was necessary to correctly interpret how the cabin air changes throughout the experiment.

The overall measured quantities are: air temperature t_a, relative humidity RH, air pressure p_a, TVOC concentration C_{TVOC} and $PM_{2.5}$ concentration C_{PM}. The temperature is measured in 18 points as described above, while the other measurements are taken in one point. The same quantities are measured also outside the cabin. Finally, the power usage of the HVAC system is also logged. Table 1 lists the measured quantities and the correspondent accuracy.

Table 1. Measured quantities.

**Variable (Unit)**	***Description***	**Specifications** ***Sensor***	***Accuracy (offset + gain)***
t_a(°C)	Air temp.	DS18B20	$\pm(0.5\,°C + 1\,\%mv^a)$
RH(%)	Air rel. hum.	BME280	$\pm(3\,\%RH + 1\,\%RH)$
p_a(hPa)	Air pres.	BME280	$\pm(1.5\,hPa + 0.12\,hPa)$
$TVOC$(ppb)	TVOC conc.	SGP30	$\pm(15\,\%mv)$
$PM_{2.5}$(µg/m^3)	PM2.5 conc.	SPS30	$\pm(10\,\mu g/m^3 + 10\,\%mv)$
P_i(kW)	Subsystem i power usage	OBD/Linux	$\pm(250\,W)$

[a] mv = measured value.

Sensor performance is a device dependent issue that can be measured with various qualifiers [13] and ideally addressed individually. In this study, the same approach for all the measured quantities has been used. A sampling time of $T_s = 10$ seconds has been adopted. The raw data from the acquisition system have been filtered with a moving mean over a 1 minute period, this leads to a 6 point moving mean. Subsequently, the filtered data have been converted to time-stamped data in tabular form, and eventually re-sampled and synchronized among the three acquisition systems. The data analysis process has been performed using open source tools, python 3.8 and several scientific computing libraries (pandas, matplotlib, numpy and scipy above all) following the exploratory data analysis (EDA) approach provided in [14].

2.2. Description of the Arduino-based system of sensors

Two independent measurement systems based on Arduino Mega 2560 were built for the measurement of environmental parameters. The systems have an on-board Real Time Clock (RTC), a data logger on flash memory, a fan and a TFT display. The RTC clocks of the two systems are constantly adjusted thanks to time data received from the

2.1. Description of the experimental setup

To characterise the thermal profile and air quality inside the vehicle cabin, two tests have been performed in outdoor parking conditions in the faculty parking lot with the front of the car oriented south.

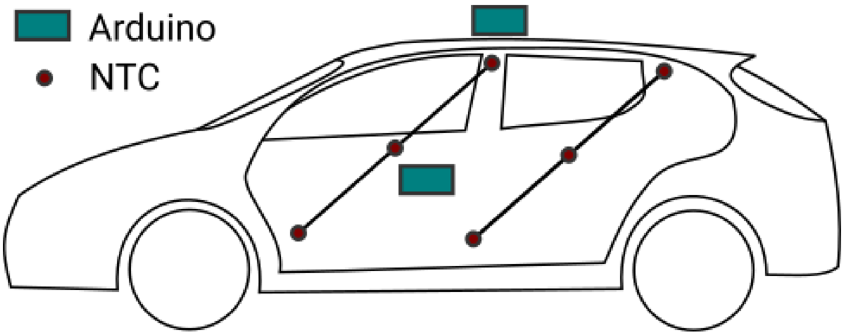

Figure 1. Lateral view of the experimental setup

The temperature distribution within the vehicle cabin is obtained with a grid of 18 DS18B20 temperature sensors following the approach used in [25] and [26] to develop a thermal model of a BEV cabin for energy consumption predictions [27]. The cabin has been ideally divided into three slices horizontally, namely top, middle and bottom level, as shown in Fig. 1 from a lateral view.

On the grid, six sensors are placed for each plane: three in the front side and three in the back side of the cabin. In addition, air quality related quantities have been measured with low-cost sensors on a unique location in the cabin; near the gear shift knob together with the acquisition system.

As external conditions can strongly affect the internal micro-climate [28], a second acquisition system has been placed on the car roof. This is identical to the internal one, except for the presence of a single temperature sensor only. The presence of the second acquisition system is needed to characterise the environment outside the vehicle and to facilitate inside/outside comparisons with data having the same structure and same metrologic fingerprint.

The approach used in the study, conversely from the one used in ISO standards regarding interior air of road vehicles [29], does not rely on a vehicle test chamber. The latter being well documented and reliable, but not suitable for real-time operation and low-cost equipment.

To have clearer insights on HVAC capabilities, an On-Board Diagnostic (OBD) Linux platform was cleverly installed inside the car to directly retrieve and collect different variables from the electronic control units [30]. Specifically, it was the *iWave OBD-II*: a little device with an *ARM Cortex-A7* processor embedded that runs a light *Yocto Poky* Linux distribution. The *iWave OBD-II* can upload data via a 4G/LTE CAT4/CAT1 sim modem, geolocate the device with a GPS receiver and it can transmit messages with the Bluetooth Low Energy 4.2 module. Communicating via the OBD-II interface, the board reads the HVAC power consumption, the power used by the auxiliary equipment (e.g., lights, infotainment, rear defroster etc.), and the power used by the heater. A fine-time granularity monitoring of those parameters was necessary to correctly interpret how the cabin air changes throughout the experiment.

The overall measured quantities are: air temperature t_a, relative humidity RH, air pressure p_a, TVOC concentration C_{TVOC} and $PM_{2.5}$ concentration C_{PM}. The temperature is measured in 18 points as described above, while the other measurements are taken in one point. The same quantities are measured also outside the cabin. Finally, the power usage of the HVAC system is also logged. Table 1 lists the measured quantities and the correspondent accuracy.

Sensor performance is a device dependent issue that can be measured with various qualifiers [31] and ideally addressed individually. In this study, the same approach for

GPS module. This operation is implemented to facilitate the synchronization of signals from the three acquisition systems.

The internal system is capable of measuring temperatures at 18 locations in the cabin (Maxim Integrated DS18B20 probes). Moreover, the internal system can measure Particulate air Matter (PM) concentration (Sensirion SPS30 sensor), air TVOC concentration (Sensirion SGP30 sensor), air CO_2 concentration (Winsen MH-Z19B non-dispersive infrared sensor), concentration formaldehyde (Winsen ZE08 sensor), air temperature, relative humidity and pressure (Bosch BME280 sensor), air flow velocity (hot wire analog sensor) and GPS position, at a single location. The external system, albeit sharing the same characteristics and using the same sensors, it lacks of the 18-spots temperature measurement, the GPS receiver and the air flow velocity sensor. Both systems are equipped with a fan that conveys air inside the device enclosure, where CO_2 and formaldehyde sensors are mounted, while the SPS30 sensor is equipped with its built-in fan. Both systems independently sampled data at 10-second intervals. All digital sensors used in the measurement device include a microcontroller that implements optimization and self-calibration algorithms.

High-precision, easy-to-use DS18B20 sensors were used to measure temperatures inside the cabin in 18 different positions; they have a typical accuracy of ±0.5°C from -10°C to +85°C and digitally transmit temperature data on a 1-Wire® bus. The use of 1-Wire protocol [15], together with the unique 64-bit serial code allows many sensors on the same bus, thus reducing the cable length and allowing to uniquely associate a sensor output with its position in the network trough a serial-position coupling. Specifically, the DS18B20 actual temperature is provided by a 12-bit analog to digital converter built-in in the digital sensor, with a fine temperature resolution up to 0.0625 °C. Its operating range is between -55°C to +125°C.

The BME280 is a high linearity and high accuracy combined temperature, humidity and pressure digital sensor. Its pressure sensing mechanism is resistive, with an operation range of 300 hPa to 1100 hPa, temperature sensing principle is of the type diode-voltage with a measurement range of −45 °C to 85 °C, measurement principle behind humidity is capacitive and its range is 0 % to 100 % [16]. It features an extremely fast response time $\tau_{63\%}$ of 1 s, thus enabling a consistent oversampling if compared with the current application time granularity of min.

The sensing principle of SPS30 PM sensor is based on laser-scattering, and allows mass concentration and number concentration sensing for particle sizes ranging from 1 µm to 10 µm. As discussed in [17] and [18], the SPS30 is an optical particle counter (OPC) optimised for PM2.5 and smaller particle analysis. Sensirion PM sensors are indeed calibrated using regularly maintained and aligned high-end reference instruments (e.g., the TSI Optical Particle Sizer Model 3330 or the TSI DustTrak™ DRX 8533) only for 2.5 µm particles size. Moreover, as stated in the sensor specification statement from the producer, PM4 and PM10 outputs are not directly measured but estimated from smaller particle counts using typical aerosol profiles. A miniaturized fan and a High Efficiency Particulate Air (HEPA) filter are included to reduce the optical part contamination; it also runs its fan at full speed for 10 seconds every 7 days as an automatic cleaning procedure. Mass concentration measurement range: 0 $\mu g/m^3$ to 1000 $\mu g/m^3$.

SGP30 TVOC sensor is a digital "multi-pixel" gas sensor. It uses multiple sensors, housed on a single metal-oxide gas sensor chip, placed on a thermally controlled hotplate. Digital data output from the sensor includes raw measurements of ethanol and H_2, and calculated values of TVOC and equivalent CO_2 via internal algorithm, such as automatic baseline compensation of the measurement [19]. The TVOC data range from this sensor is between 0 to 60000 ppb. This sensor's equivalent CO_2 were disregarded due to its low sensitivity to external pollutants and due to the absence of passengers in parking conditions.

Table 1. Measured quantities.

Variable (Unit)	*Description*	Sensors specifications *Manufacturer*	*Model*	*Accuracy (offset + gain)*
t_a(°C)	Air temp.	Maxim Integrated	DS18B20	$\pm(0.5\,°C + 1\,\%mv^a)$
RH(%)	Air rel. hum.	Bosch	BME280	$\pm(3\,\%RH + 1\,\%RH)$
p_a(hPa)	Air pres.	Bosch	BME280	$\pm(1.5\,hPa + 0.12\,hPa)$
$TVOC$(ppb)	TVOC conc.	Sensirion	SGP30	$\pm(15\,\%mv)$
$PM_{2.5}(\mu g/m^3)$	PM2.5 conc.	Sensirion	SPS30	$\pm(10\,\mu g/m^3 + 10\,\%mv)$
P_i(kW)	Subsystem i power usage	iWave	OBD/Linux	$\pm(250\,W)$

[a] mv = measured value.

all the measured quantities has been used. A sampling time of $T_s = 10$ seconds has been adopted. The raw data from the acquisition system has been filtered with a moving mean over a 1-minute period, this leads to a 6-point moving mean. Subsequently, the filtered data has been converted into time-stamped data in tabular form, and eventually re-sampled and synchronised among the three acquisition systems. The data analysis process has been performed using open-source tools, python 3.8 and several scientific computing libraries (pandas, matplotlib, numpy and scipy above all) following the exploratory data analysis (EDA) approach provided in [32].

2.2. Description of the Arduino-based system of sensors

Two independent measurement systems based on Arduino Mega 2560 were built for the measurement of environmental parameters. The systems have an on-board Real Time Clock (RTC), a data logger on flash memory, a fan and a TFT display. The RTC clocks of the two systems are constantly adjusted thanks to time data received from the GPS module. This operation is implemented to facilitate the synchronization of signals from the three acquisition systems.

The internal system is capable of measuring temperatures at 18 locations in the cabin (Maxim Integrated DS18B20 probes). Moreover, the internal system can measure Particulate air Matter (PM) concentration (Sensirion SPS30 sensor), air TVOC concentration (Sensirion SGP30 sensor), air CO_2 concentration (Winsen MH-Z19B non-dispersive infrared sensor), concentration formaldehyde (Winsen ZE08 sensor), air temperature, relative humidity and pressure (Bosch BME280 sensor), air flow velocity (hot wire analog sensor) and GPS position, at a unique location. The external system, albeit sharing the same characteristics and using the same sensors, it lacks of the 18-spots temperature measurement, the GPS receiver and the air flow velocity sensor. Both systems are equipped with a fan that conveys air inside the device enclosure, where CO_2 and formaldehyde sensors are mounted, while the SPS30 sensor is equipped with its built-in fan. Both systems independently sampled data at 10-second intervals. All digital sensors used in the measurement device include a microcontroller that implements optimisation and self-calibration algorithms.

High-precision, easy-to-use DS18B20 sensors were used to measure temperatures inside the cabin in 18 distinct positions; they have a typical accuracy of $\pm0.5\,°C$ from $-10\,°C$ to $85\,°C$ and digitally transmit temperature data on a 1-Wire® bus. The use of 1-Wire protocol [33], together with the unique 64-bit serial code allows many sensors on the same bus, thus reducing the cable length and allowing to uniquely associate a sensor output with its position in the network through a serial-position coupling. Specifically, the DS18B20 actual temperature is provided by a 12-bit analog to digital converter built-in in the digital sensor, with a fine temperature resolution up to 0.0625 °C. Its operating range is between $-55\,°C$ to $125\,°C$.

The BME280 is a high linearity and high accuracy combined temperature, humidity and pressure digital sensor. Its pressure sensing mechanism is resistive, with an

operation range of 300 hPa to 1100 hPa, temperature sensing principle is of the type diode-voltage with a measurement range of −45 °C to 85 °C, measurement principle behind humidity is capacitive and its range is 0 % to 100 % [34]. It features an extremely fast response time $\tau_{63\%}$ of 1 s, thus enabling a consistent oversampling if compared with the current application time granularity of 1 min.

The sensing principle of SPS30 PM sensor is based on laser-scattering, and allows mass concentration and number concentration sensing for particle sizes ranging from 1 µm to 10 µm. As discussed in [35] and [21], the SPS30 is an optical particle counter (OPC) optimised for PM2.5 and smaller particle analysis. Sensirion PM sensors are indeed calibrated using regularly maintained and aligned high-end reference instruments (e.g., the TSI Optical Particle Sizer Model 3330 or the TSI DustTrak™ DRX 8533) only for 2.5 µm particles size. Moreover, as stated in the sensor specification sheet from the manufacturer, PM4 and PM10 outputs are not directly measured but estimated from smaller particle counts using typical aerosol profiles. A miniaturized fan and a High Efficiency Particulate Air (HEPA) filter are included to reduce the optical part contamination; it also runs its fan at full speed for 10 seconds every 7 days as an automatic cleaning procedure. Mass concentration measurement range: 0 µg/m^3 to 1000 µg/m^3.

SGP30 TVOC sensor is a digital "multi-pixel" gas sensor. It uses multiple sensors, housed on a single metal-oxide gas sensor chip, placed on a thermally controlled hotplate. Digital data output from the sensor includes raw measurements of ethanol and H_2, and calculated values of TVOC and equivalent CO_2 via internal algorithm, such as automatic baseline compensation of the measurement [19]. The TVOC data range from this sensor is between 0 to 60000 ppb. This sensor's equivalent CO_2 were disregarded due to its low sensitivity to external pollutants and due to the absence of passengers in parking conditions.

The measurement system has been characterised both in winter and in summer conditions. In the following sections, two typical conditions for winter and summer have been chosen, to characterise the HVAC system performance in heating and cooling operations, respectively. Winter tests have been performed on January 29, 2021, while summer test have been carried out from July 14 to July 15, 2021.

3. Results and discussion

In this section, the combined measures of air quality and comfort parameters, together with the energy consumption by the electric car are shown and discussed in different seasons, in order to show the differences in relation to the operational mode for the air conditioning. Moreover, an estimation of the filtration performances of the HVAC system is given, by comparing the results corresponding to new and used filters.

3.1. Measurements during heating operation

Two different test conditions have been investigated, for the winter, starting from a state of equilibrium with the external environment, obtained maintaining all systems off and all doors opened for 15 minutes. Once the equilibrium was reached, the proper test was performed while maintaining the heater on for 1 hour, the set-point temperature at its maximum of 30 °C, the fan speed at its maximum (position 7), all the windows and all the doors closed. During the first test, the recirculation system was off (meaning that the air ventilation system was in fresh-air configuration), during the second test the recirculation system was instead on.

3.1.1. Fresh-air configuration

The temperature measured inside and outside the cabin keeping the fresh-air mode is shown in Fig. 2(a). The air temperature inside the cabin is obtained by the average of the air temperature measurements on the sensors placed on the grid shown in Fig. 1, *i.e.*, $t_{int} = t_{avg}$. The TVOC concentration measured inside and outside the cabin in fresh-air mode are shown in Fig. 2(b). It is shown that the TVOC concentration increases

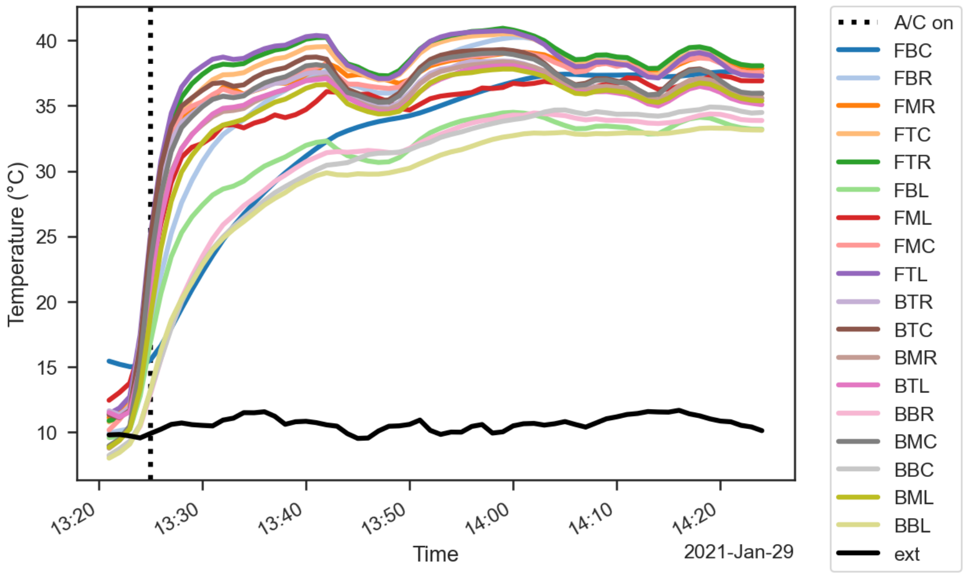

Figure 2. Heating temperature profiles, fresh-air mode.

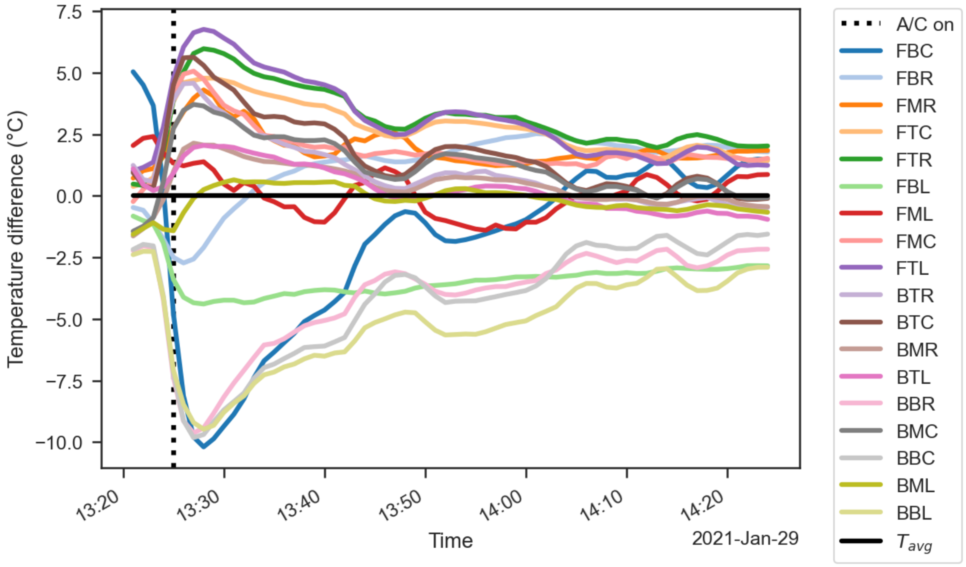

Figure 3. Heating temperature difference profiles, fresh-air mode.

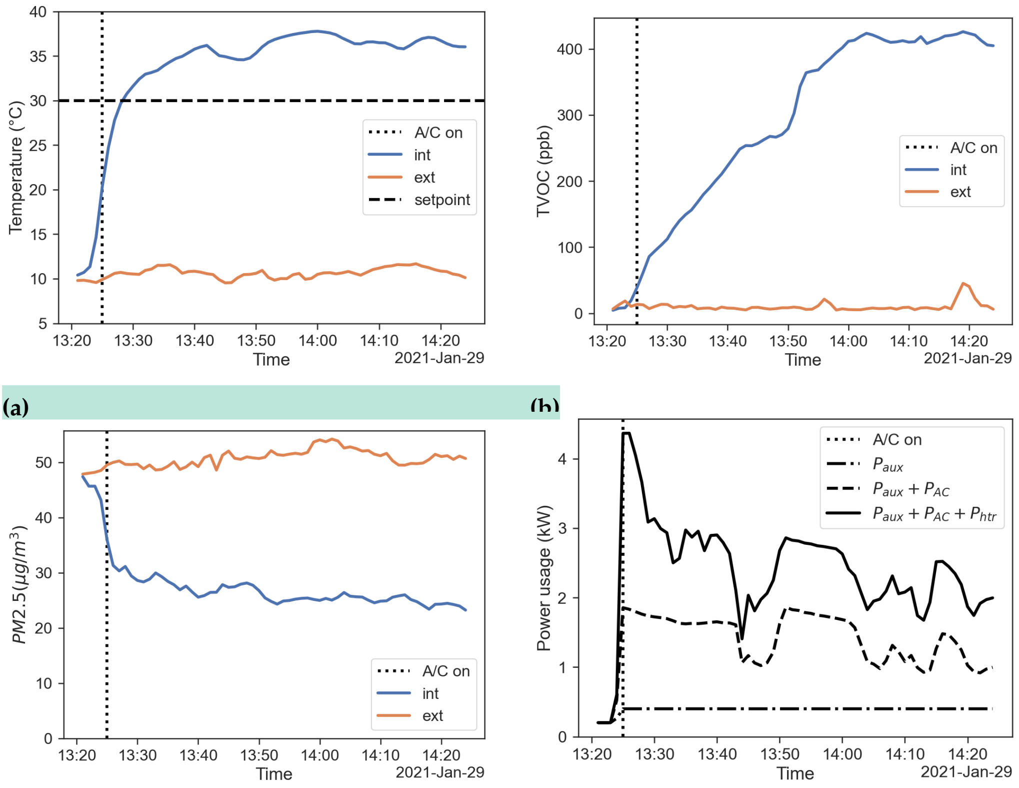

(a) **(b)**

(c) **(d)**

Figure 2. Results regarding the fresh-air mode. (a) Temperature inside (blue) and outside (red) the cabin; (b) TVOC concentration inside (blue) and outside (red) the cabin; (c) PM2.5 concentration inside (blue) and outside (red) the cabin; (d) power usage of the HVAC system.

while the HVAC system is working, even with a fresh-air mode. This effect is related to the presence of sources of VOC inside the vehicle cabin and build up phenomena during HVAC operation, as expected from the literature [29]. Figure 2(c) displays the PM2.5 concentration measured inside and outside the cabin in fresh-air mode. Figure shows that the PM concentrations decrease while the HVAC system is working, due to the filtering activity of the HVAC filter. This result ties with what is found in the literature [10]. For the case analysed, a filtration efficiency of about $\eta = 0.5$ for PM2.5 is ascertained. The power usage of the HVAC system, with the contributions of power used by auxiliary equipment, A/C system and PTC heater recorded by the OBD system is shown in the stacked line plot in Fig. 2(d).

3.1.2. Recirculation configuration

Similar considerations can be made for the case with recirculation activated. From the results shown in Figure 3 it is clear that the time to steady state is close to 20 min; again the over-temperature issue remains significant. TVOC concentration reached a value similar to the case without recirculation, but in a longer time with respect to the fresh-air mode. A possible explanation for this behaviour could rely on the fact the source of VOCs inside the cabin is compensated by an improved adsorption performance, as observed by [10]. The PM concentrations decrease to lower values with respect to the fresh-air mode, as shown in Fig. 3(c). This result shows that the filtration performance is improved by the recirculation mode. Figure 3(d) shows the power usage of the HVAC system. The figure shows contributions of auxiliary equipment, A/C system and PTC heater to the overall power usage.

3. Results

The measurement system has been characterised both in winter and in summer conditions. In the following sections, two typical conditions for winter and summer have been chosen, to characterise the HVAC system performance in heating and cooling operations respectively. Winter tests have been performed on January 29, 2021, while summer test have been carried out from July 14 to July 15, 2021.

3.1. Heating operation

Amid winter period, two different test conditions have been investigated, starting from a state of equilibrium with the external environment, obtained maintaining all systems off and all doors opened for 15 minutes. Once the equilibrium was reached, the proper test was performed while maintaining the heater on for 1 hour, the set-point temperature at its maximum of 30 °C, the fan speed at its maximum, all the windows and all the doors closed. During the first test, the recirculation system was off (that means that the air ventilation system was in fresh-air configuration), while during the second test the recirculation system was on.

3.1.1. Fresh-air configuration

All the experiments confirm that the heating system is capable of reaching a quasi-steady state condition in about 20 min, even though the temperature reached by the air inside the cabin is well over the set-point temperature. Figure 2 shows the readings from all the 18 temperature sensors in the cabin for the case with fresh-air mode with a 3 letters naming scheme:

1. The first letter tells us if we are in the front (F) or in the back (B) of the cabin.
2. The second letter refers to the location in vertical direction, namely bottom (B), middle (M) and top (T) plane.
3. The third letter refers to the location in the horizontal direction, namely left (L) or driver, center (C) or right (R) side.

Colder spots can be found at feet level of the back seats, moreover this is in disagreement with the studies of Nilsson on equivalent temperature of body segments [20]. In other words, analysing the results of the work that led to the development of the only available standard for comfort evaluation in vehicles (ISO-14505), we can infer that occupants will be likely to accept colder temperatures in upper body parts during winter, while here we are in the opposite situation. Data from position FBC have a different behaviour because it refers to the sensor installed inside the acquisition system box, thus suffering from thermal inertia issues.

Defining a temperature difference $\Delta t = t_i - t_{avg}$; where t_i is the temperature in one generic position of the grid, and t_{avg} the mean value of the 18 temperature readings for each timestamp; is possible to obtain Figure 3. It is worth noting that air temperature inside the cabin can reach discrepancies of more than 15 °C in the first minutes of operation, while the Δt values at the end of test is lower and the temperature distribution much more uniform.

Figure 4(a) shows the temperature measured inside and outside the cabin keeping the fresh-air mode. In the following air temperature inside the cabin is considered equal to the average of all the sensors on the grid $t_{int} = t_{avg}$.

Figure 4(b) reports TVOC concentration measured inside and outside the cabin in fresh-air mode. The system is able to detect a TVOC concentration increase while the HVAC system operates, even with an external air mixing. This behaviour confirms the presence of a source of VOC inside the Leaf's cabin and a build up phenomenon during HVAC operation, as expected from literature [11].

Figure 4(c) displays the PM2.5 concentration measured inside and outside the cabin in fresh-air mode. This result ties with what is found in literature [21], the cabin filter is well capable of lowering the PM concentration inside the car with a steady state filtration efficiency of about $\eta = 0.5$.

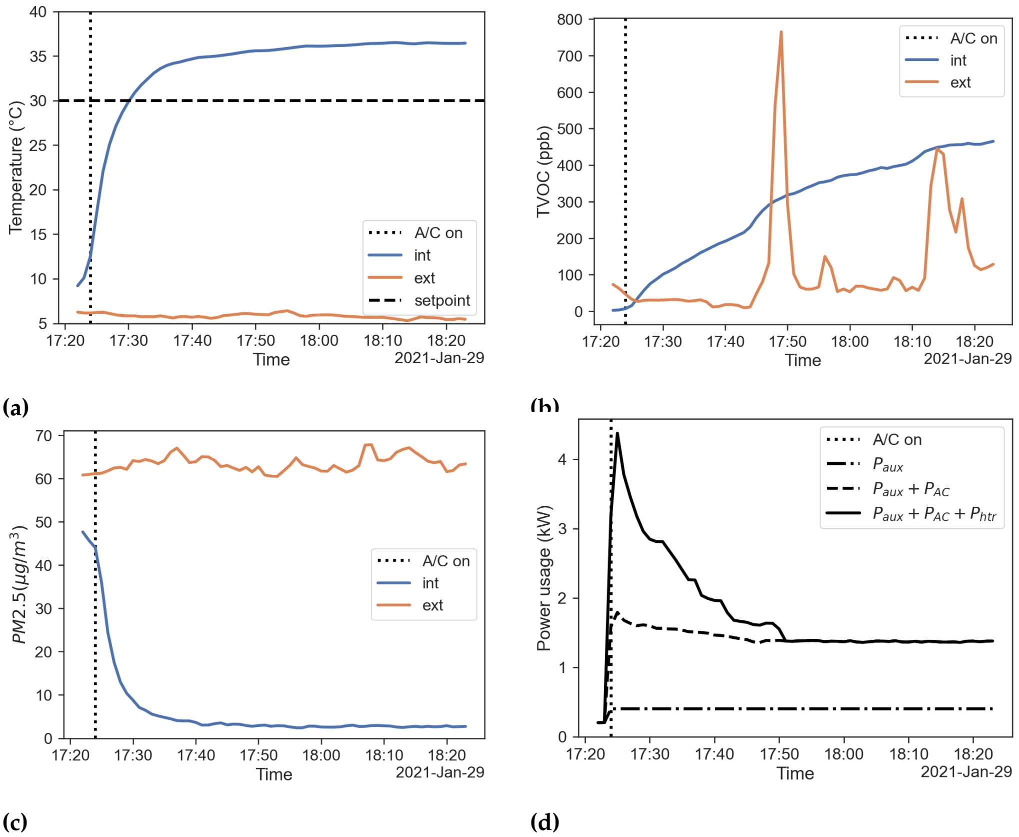

Figure 3. Results regarding the recirculation mode. (a) Temperature inside (blue) and outside (red) the cabin; (b) TVOC concentration inside (blue) and outside (red) the cabin; (c), PM2.5 concentration inside (blue) and outside (red) the cabin; (d) power usage of the HVAC system.

3.1.3. Comparison between fresh-air and recirculation mode in winter

The open-field tests conducted in this work have been chosen because representative of the real operating conditions of the vehicle. On the other hand, the experiments have been performed with no control on the environment outside the cabin, with repeatability issues. In order to compare the experiments, the following dimensionless temperature is defined:

$$t^* = \frac{t_{int} - t_{ext}}{t_{set} - t_{ext}} \tag{1}$$

where t^* is the dimensionless temperature, t_{int} is the air temperature measured inside the cabin, t_{ext} is the air temperature measured outside the cabin and t_{set} is the set-point temperature. It is worth to underline that when $t_{int} = t_{ext}$, dimensionless temperature t^* is equal to 0, while when $t_{int} = t_{set}$, then t^* is equal to 1. These two key points represent two relevant physical states, equilibrium with the external environment and fulfilment of the set-point request, respectively. Figure 4(a) shows a comparison between the dimensionless temperatures obtained for the two experiments. It is noticeable that the dimensionless temperature obtained without recirculation is always higher than the one obtained in the case of recirculation mode, thus suggesting that the over-temperature issue is more significant in this case. In addition, the set-point is reached faster during the fresh-air mode than during the recirculation mode.

The filtration efficiency of the vehicle can be defined using a black box approach, where the vehicle cabin is considered as a system with an unknown filtration capacity, while inlet (external) and outlet (internal) concentrations are known. The filtration efficiency is then defined by

$$\eta = 1 - \frac{C_{int}}{C_{ext}} \tag{2}$$

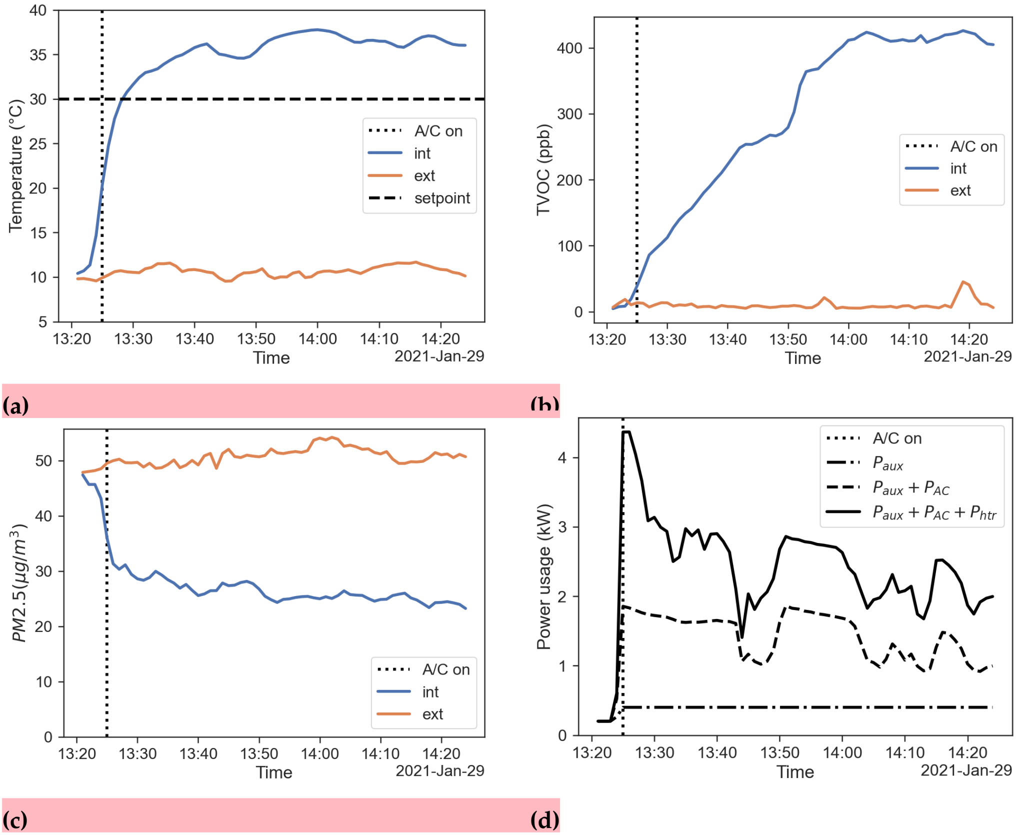

Figure 4. Results regarding the fresh-air mode. (a) Temperature inside (blue) and outside (red) the cabin; (b) TVOC concentration inside (blue) and outside (red) the cabin; (c) PM2.5 concentration inside (blue) and outside (red) the cabin; (d) power usage of the HVAC system.

A first example of what kind of data can be acquired with the OBD system is given in figure 4(d), where stacked plots of the power usage of the HVAC system are shown.

3.1.2. Recirculation configuration

Figure 5 shows the readings from all the 18 temperature sensors in the cabin for the case with recirculation mode. Air temperature inside the cabin shows slightly higher discrepancies with respect to the previous case, colder spots can be found again at feet level of the back seats. Signal from the FBC sensor shows similar responsiveness issues as for the case without recirculation. Higher temperature gradients in the startup phase are evident from Figure 6, while a smoother profile is reached at the end of test.

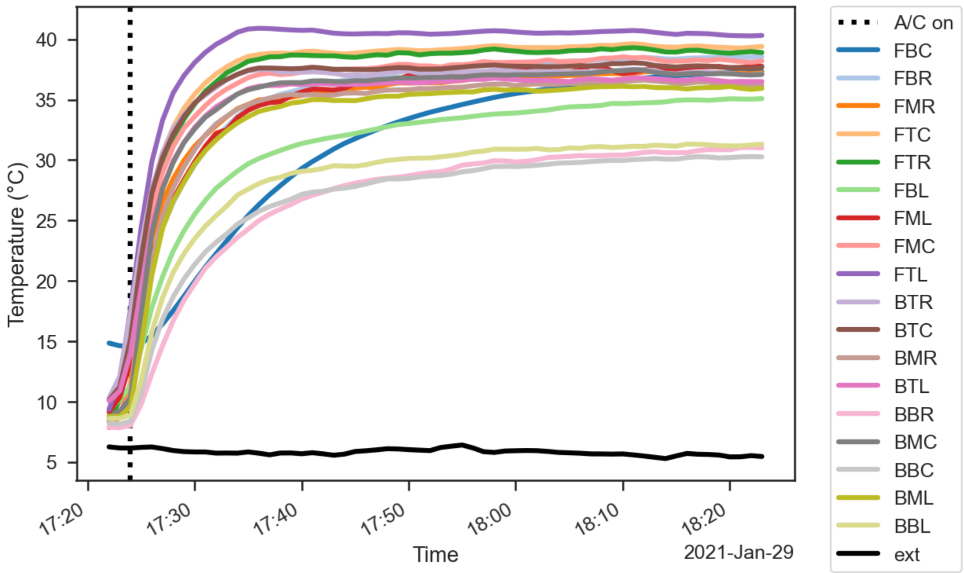

Figure 5. Heating temperature profiles, recirculation mode.

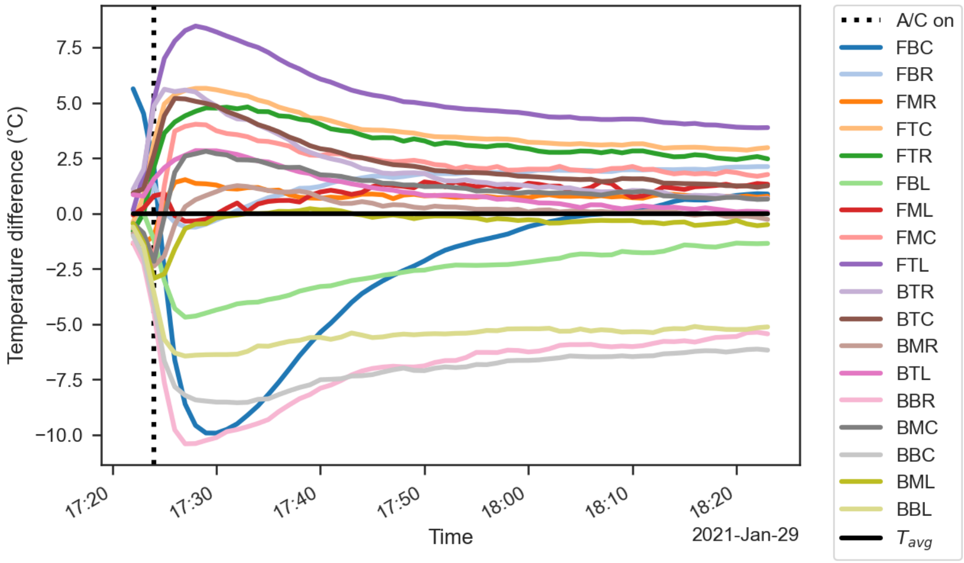

Figure 6. Heating temperature difference profiles, recirculation mode.

Similar considerations can be made for the case with recirculation activated. From the results shown in Figure 7 it is clear that the time to steady state is close to 20 min; again the over-temperature issue remains significant. TVOC concentration reached a similar value as the case without recirculation, but slower. A possible explanation for

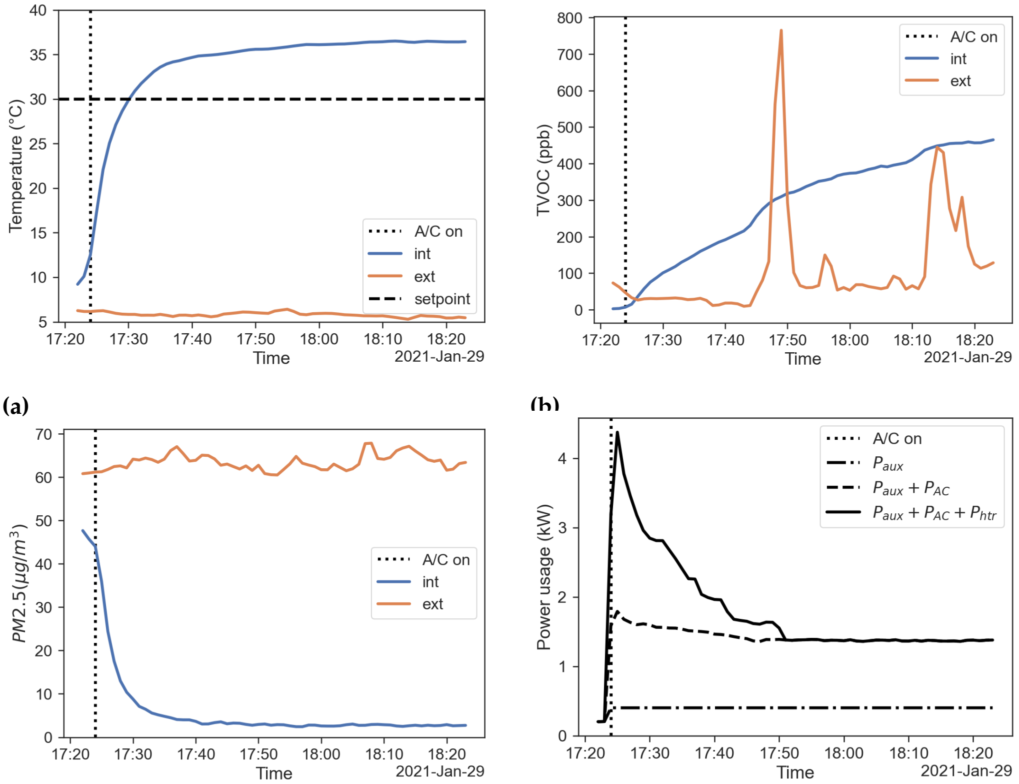

(a) **(b)**

(c) **(d)**

Figure 7. Results regarding the recirculation mode. (a) Temperature inside (blue) and outside (red) the cabin; (b) TVOC concentration inside (blue) and outside (red) the cabin; (c), PM2.5 concentration inside (blue) and outside (red) the cabin; (d) power usage of the HVAC system.

this behaviour could rely on the fact that even without fresh air mixing, the source of VOCs inside the cabin is compensated by an improved adsorption performance in recirculation mode. This happens mostly if the breakthrough time is not yet reached [21]. As for the PM values, it is pretty clear from Fig. 7(c) how the filtration performance is improved by the recirculation mode.

3.1.3. Comparison between fresh-air and recirculation mode in winter

The open field tests conducted in this work share similarities with the real operating conditions of the vehicle. On the other hand, the experiments have been performed with no control on the environment outside the cabin, with repeatability issues. In order to compare the experiments, the following dimensionless temperature is defined:

$$t^* = \frac{t_{int} - t_{ext}}{t_{set} - t_{ext}} \tag{1}$$

where t^* is the dimensionless temperature, t_{int} is the air temperature measured inside the cabin, t_{ext} is the air temperature measured outside the cabin and t_{set} is the set-point temperature. It is worth to underline that when $t_{int} = t_{ext}$, dimensionless temperature t^* is equal to 0, while when $t_{int} = t_{set}$, then t^* is equal to 1. These two key points represent two relevant physical states, equilibrium with the external environment and fulfilment of the set-point request respectively. Figure 8 shows a comparison between the dimensionless temperatures obtained for the two experiments. It is noticeable that the

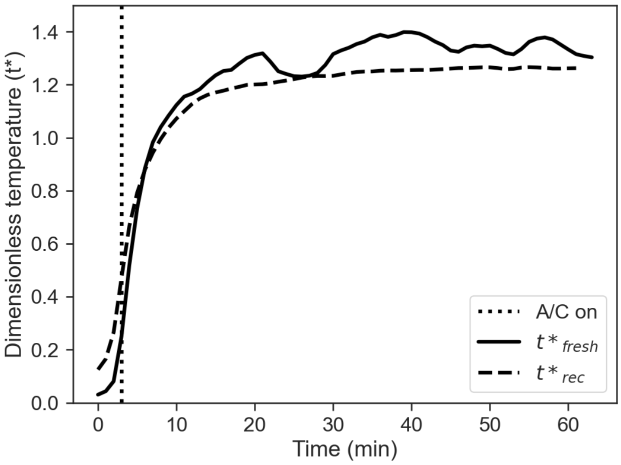

Figure 8. Dimensionless temperature profiles, comparison between fresh-air mode (solid line) and recirculation (dashed line) mode.

dimensionless temperature obtained without recirculation is always higher than the one obtained in the case of recirculation mode, thus suggesting that the over-temperature issue is more significant in this case. In addition, the set-point is reached faster during the fresh-air mode than during the recirculation mode.

The filtration efficiency of the vehicle can be defined using a black box approach, where the vehicle cabin is considered as a system with an unknown filtration capacity, while inlet (external) and outlet (internal) concentrations are known:

$$\eta = 1 - \frac{C_{int}}{C_{ext}} \tag{2}$$

Figure 9 shows a comparison between the two cases. The PM filtration efficiency with recirculation mode is almost double than the one with the fresh-air mode. It is also noticeable that the filtration efficiency does never reach the ideal value of $\eta = 1$, suggesting that infiltration rate not equal to zero occur even if the vehicle is parked.

An alternative method to get insights about IAQ of a vehicle cabin relies on a time integrated inside/outside approach [3]. The associated index, named Cabin Air Quality Index (CAQI) is defined as follows:

$$CAQI = \frac{\int_{t_i}^{t_f} C_{int}(t)\,dt}{\int_{t_i}^{t_f} C_{ext}(t)\,dt} \tag{3}$$

Results based on this index for PM2.5 and TVOC are given in Figures 10 and 11.

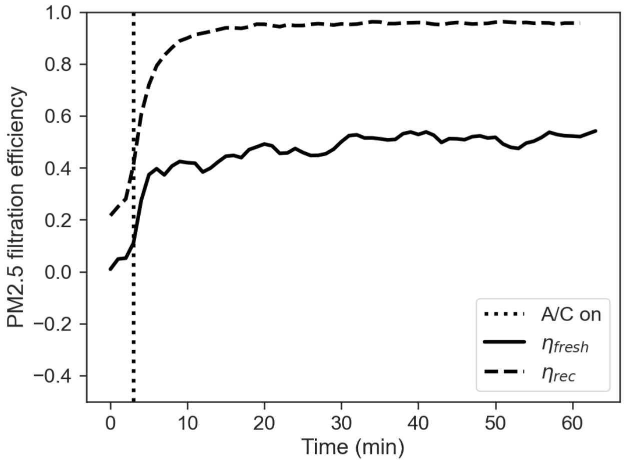

Figure 9. Filtration efficiency, comparison between fresh-air (solid line) and recirculation (dashed line) mode.

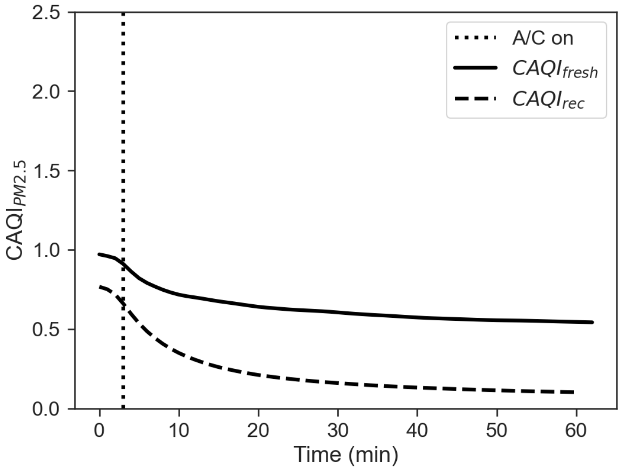

Figure 10. CAQI for PM2.5.

Figure 12 shows the comparison between the cumulative energy consumption in the two cases of recirculation on and off, calculated as the approximate cumulative integral of $P_{tot} = P_{aux} + P_{AC} + P_{Htr}$ via the trapezoidal method in order to integrate numeric data rather than a functional expression:

$$E = \int_{t_i}^{t_f} P_{tot}(t)\,dt \approx \frac{t_f - t_i}{2N} \sum_{n=1}^{N} (P_{tot}(t_n) + P_{tot}(t_{n+1})) \tag{4}$$

where t_i is the start time, t_f the final time and N+1 the number of samples available (equally spaced). The total energy consumption obtained in the recirculation mode on is about 3/4 of the value obtained for the fresh-air mode. This result can explain what is shown in figures 4(d) and 7(d). These figures show that the power usage from the

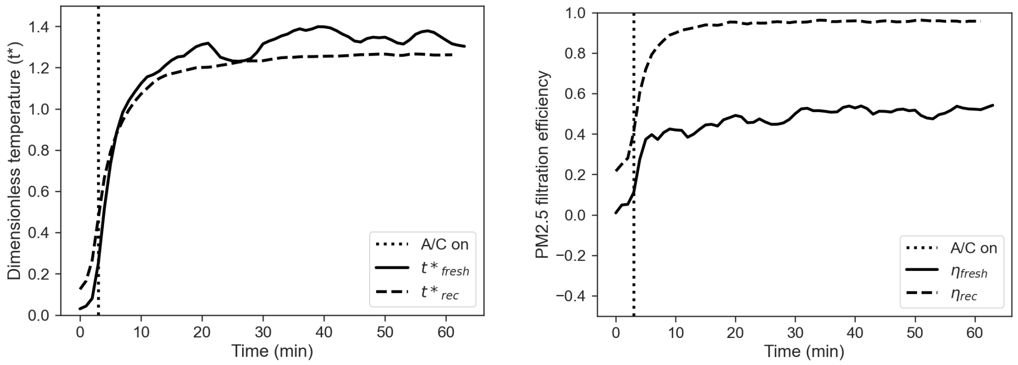

(a) **(b)**

Figure 4. (a) Dimensionless temperature profiles, comparison between fresh-air (solid line) and recirculation (dashed line) mode. (b) Filtration efficiency, comparison between fresh-air (solid line) and recirculation mode (dashed line).

where C_{int} and C_{ext} are the internal and external concentrations, respectively. Figure 4(b) shows a comparison between the PM filtration efficiency obtained in the two regimes. The figure shows that PM filtration efficiency with recirculation mode is almost double than the one obtained with the fresh-air mode. It is also noticeable that the filtration efficiency does never reach the ideal value of $\eta = 1$, suggesting that infiltration rate not equal to zero occur even if the vehicle is parked.

An alternative method to get insights about IAQ of a vehicle cabin relies on a time integrated inside/outside approach proposed in [13]. The associated index, named Cabin Air Quality Index (CAQI) is defined as follows:

$$CAQI = \frac{\int_{t_i}^{t_f} C_{int}(t)\, dt}{\int_{t_i}^{t_f} C_{ext}(t)\, dt} \tag{3}$$

where C_{int} is the internal concentration , C_{ext} is the external concentration, t_i is the start time and t_f is the stop time. Results based on this index for PM2.5 and TVOC are given in Figure 5. The figure shows that the CAQI indexes for PM2.5 and VOC obtained for

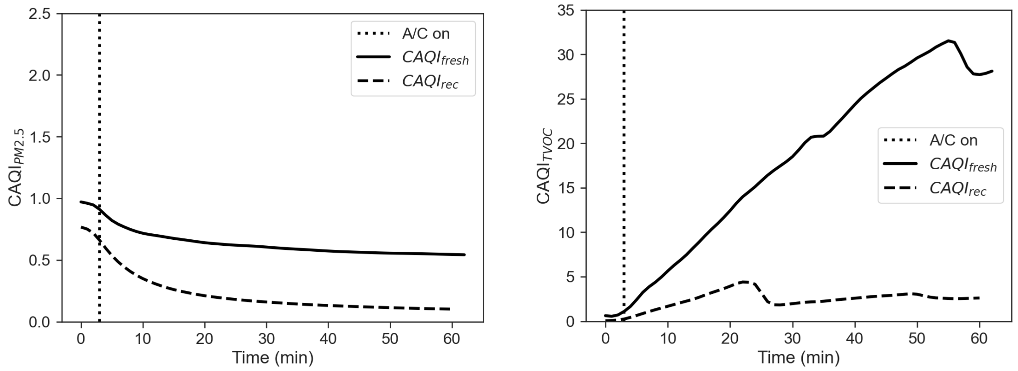

(a) **(b)**

Figure 5. (a) CAQI for PM2.5 and (b) CAQI for TVOC.

the fresh-air mode are much greater than the one obtained for the recirculation mode. Figure 6 shows the comparison between the cumulative energy consumption in the two cases of recirculation on and off, calculated as the approximate cumulative integral of

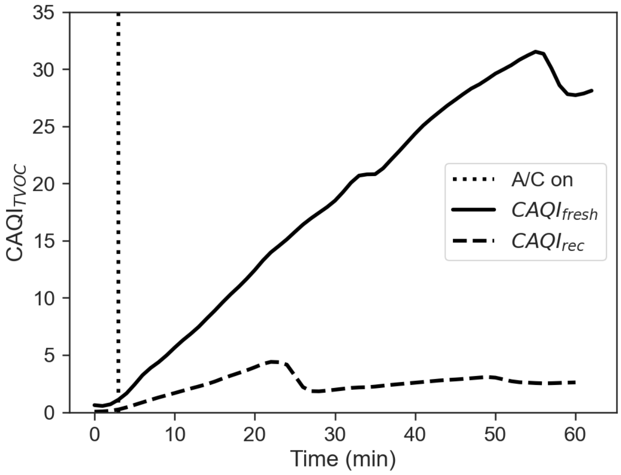

Figure 11. CAQI for TVOC.

HVAC system is similar for the two modes in the first minutes of operation. However, when the effects of recirculation become prevalent, the values of the HVAC power usage related to the two modes differ. Infact, while P_{tot} peaks at more than 4 kW in the first minutes of operation in both modes, it varies significantly towards the end of the test.

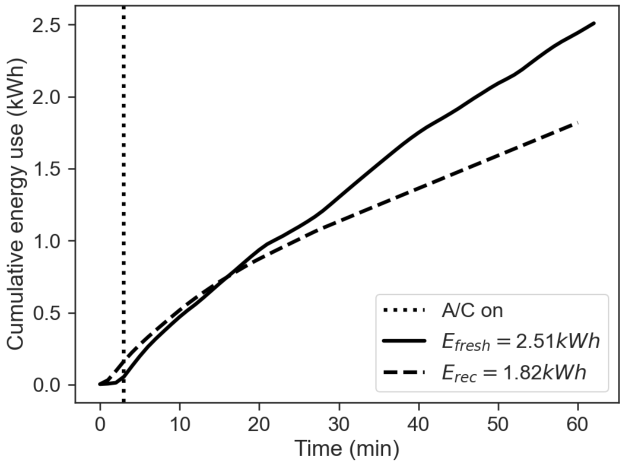

Figure 12. Cumulative energy use, comparison between fresh-air (solid line) and recirculation (dashed line) mode.

3.2. *Cooling operation*

Two different test conditions have been investigated starting from a state of equilibrium with the external environment, obtained maintaining all systems off and all doors opened for 15 minutes. Once the equilibrium was reached, the proper test was performed while maintaining the A/C on for 1 hour, the set-point temperature at its minimum of 16 °C, the fan speed at its maximum, all the windows and all the doors closed. During the first test, the recirculation system was off (that means that the air ventilation system was in fresh-air configuration), while during the second test the recirculation system was on.

3.2.1. Recirculation mode off

All the experiments confirm that the cooling system is not capable of reaching a quasi-steady state condition in about 60 min, textiti.e. the temperature reached by the air inside the cabin is far from the set-point temperature value. Figure 13 shows the

$P_{tot} = P_{aux} + P_{AC} + P_{Htr}$ via the trapezoidal method, in order to integrate numeric data rather than a functional expression:

$$E = \int_{t_i}^{t_f} P_{tot}(t)\,dt \approx \frac{t_f - t_i}{2N} \sum_{n=1}^{N} \left(P_{tot}(t_n) + P_{tot}(t_{n+1})\right) \quad (4)$$

where t_i is the start time, t_f the final time and N+1 the number of samples available (equally spaced). The total energy consumption obtained in the recirculation mode is about 3/4 of the value obtained for in fresh-air mode. This result can explain what is shown in figures 2(d) and 3(d). These figures show that the power usage from the HVAC system is similar for the two modes in the first minutes of operation. However, when the effects of recirculation become prevalent, the values of the HVAC power usage related to the two modes differ considerably. In fact, while P_{tot} peaks at more than 4 kW in the first minutes of operation in both modes, it varies significantly towards the end of the test.

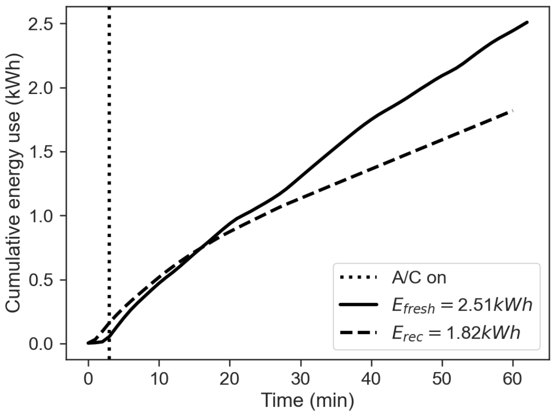

Figure 6. Cumulative energy use, comparison between fresh-air (solid line) and recirculation (dashed line) mode.

3.2. *Measurements during cooling operation*

Two different test conditions have been investigated starting from a state of equilibrium with the external environment, obtained maintaining all systems off and all doors opened for 15 minutes. Once the equilibrium was reached, the proper test was performed while maintaining the A/C on for 1 hour, the set-point temperature at its minimum of 16 °C, the fan speed at its maximum (position 7), all windows and all doors closed. During the first test, the recirculation system was off (that means that the air ventilation system was in fresh-air configuration), while during the second test the recirculation system was on.

3.2.1. Fresh-air configuration

All the experiments confirm that the cooling system is not capable of reaching a quasi-steady state condition in about 60 min, *i.e.* the temperature reached by the air inside the cabin is far from the set-point temperature value.

Figure 7(a) shows the temperature measured inside and outside the cabin keeping the fresh-air mode. Like for the winter operation, the air temperature inside the cabin is calculated as the mean value of the 18 temperature readings for each timestamp.

Figure 7(b) shows TVOC measured inside and outside the cabin in fresh-air mode. The figure reports that the TVOC concentration is higher than that of the external air at the beginning, but decreases while the HVAC system operates. The lowest concentration is reached, despite the fluctuation on the outside. This behaviour can be explained with a drop of temperature inside the cabin combined with fresh-air mixing, thus reducing the emission from the internal sources.

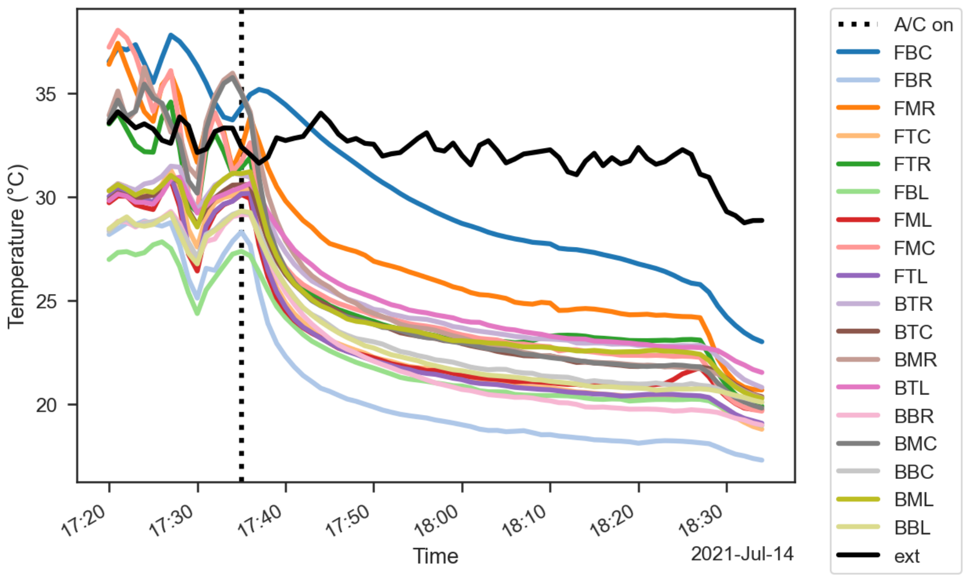

Figure 13. Cooling temperature profiles, fresh-air mode.

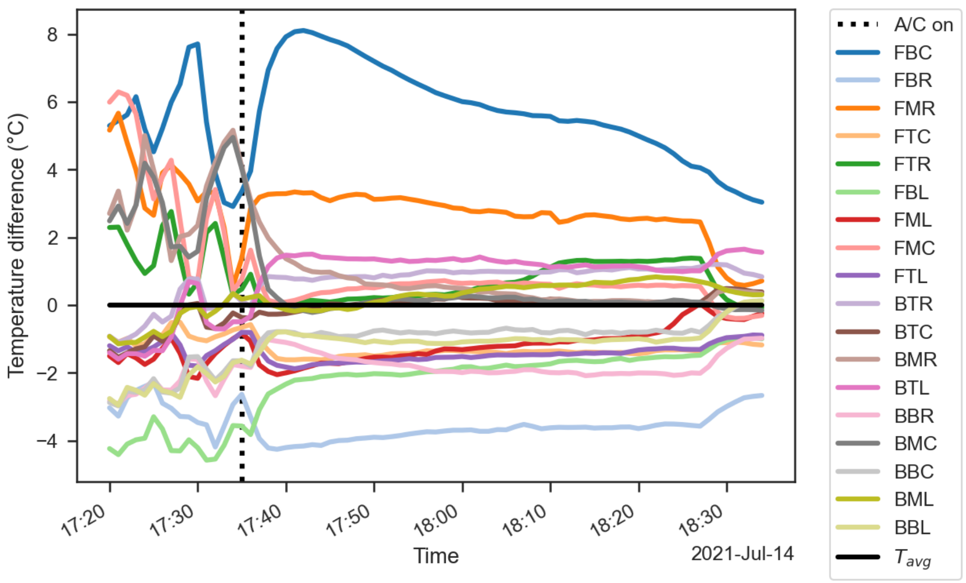

Figure 14. Cooling temperature difference profiles, fresh-air mode.

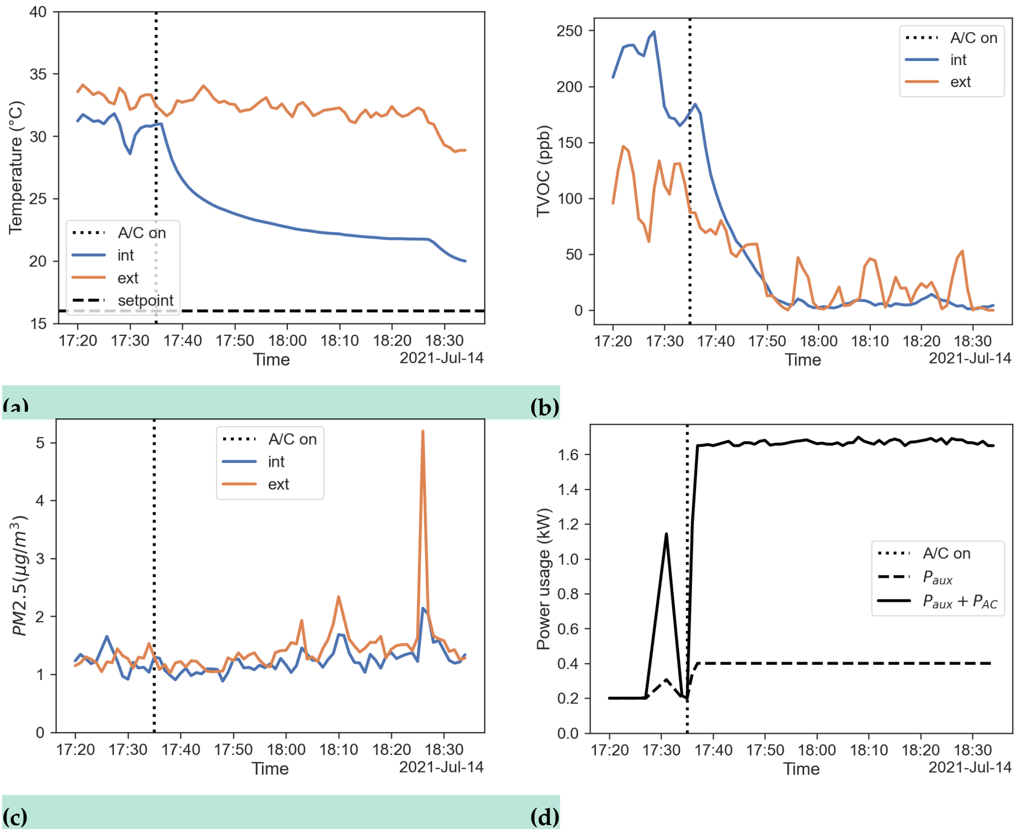

Figure 7. Results regarding the fresh-air mode. (a) Temperature inside (blue) and outside (red) the cabin; (b) TVOC concentration inside (blue) and outside (red) the cabin; (c) PM2.5 concentration inside (blue) and outside (red) the cabin; (d) power usage of the HVAC system.

Figure 7(c)reports plots of the PM2.5 concentration measured inside and outside the cabin in fresh-air configuration. This result appears to be in contrast with the results shown by [10], *i.e.* the cabin filter is not able to lower the PM concentration inside the car with a steady state filtration efficiency $\eta = 0$ to 0.2. Here we need to consider that the value of PM2.5 concentrations measured were extremely low and under the sensor precision for that particle size range ($\pm 10\,\mu g/m^3$).

Figure 7(d) shows the power usage of the HVAC system. The figure shows the contributions of power used by auxiliary equipment and A/C system; with PTC heater power being indeed equal to zero in cooling operation.

3.2.2. Recirculation configuration

As for the previous case with recirculation activated, time to steady state is close to 20 min of operation, while the over-temperature issue is still significant. TVOC concentration shows a different trend. Despite external concentration peaks at the end of the test, the internal one remains quite low. As for PM values, they follow a completely different trend, it is pretty clear from Fig. 8(c) how the filtration performance is improved by the recirculation mode, even with absolute values well within the precision range as is the previous test. Figure 8(d) shows the power usage of the HVAC system, with the contributions of power used by auxiliary equipment and A/C system recorded by the OBD system.

3.2.3. Comparison between fresh-air and recirculation modes in summer

Experiments in cooling as well as heating mode have been performed in real parking conditions with no control on the environment outside the cabin, repeatability issues are worsen by the increased contribution of solar load in summer. In order to compare the

readings from all the 18 temperature sensors in the cabin for the case with fresh-air mode with the same naming scheme introduced in the previous section.

Temperature discrepancies in the cabin can approach 12 °C; warmer spots can be found at top and middle locations, while bottom zone is generally colder (Fig. 14). To a certain extent, this is in agreement with a stratification of cabin air during operation, despite the vents working at full power. Looking at "Light summer" Comfort Zones defined by [20], we can infer that occupants will be likely to accept warmer temperatures in lower body parts during summer, while this result goes in the opposite direction. Self heating and thermal inertia issue of the sensor positioned at FBC position are still evident.

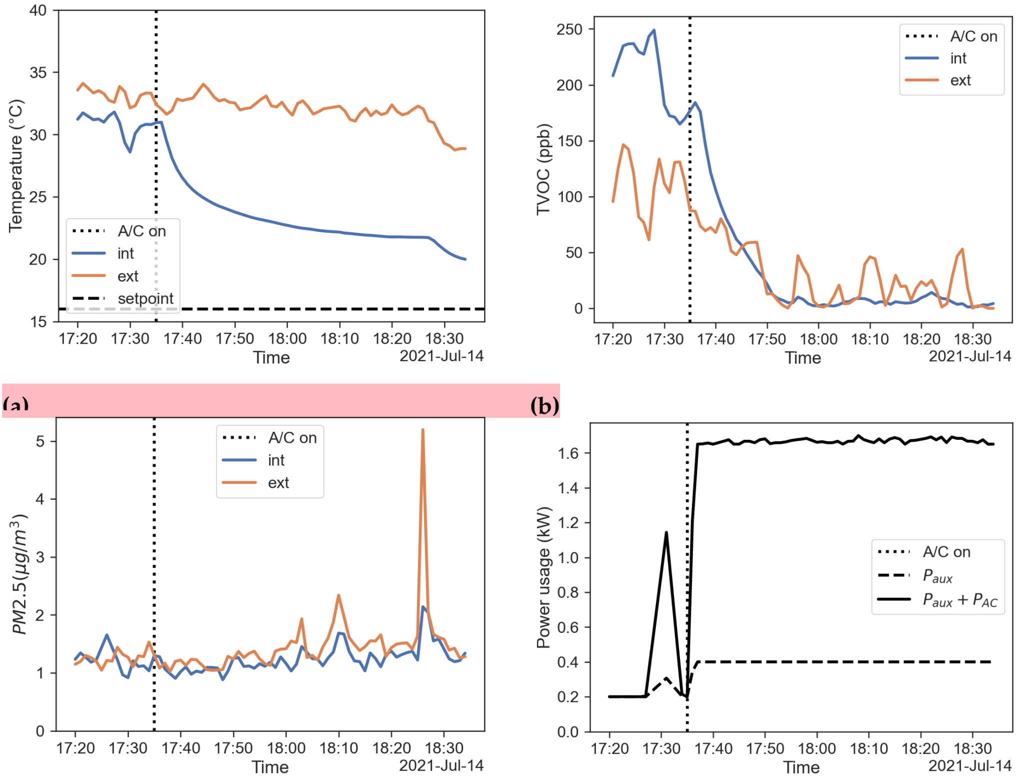

(a) (b)

(c) (d)

Figure 15. Results regarding the fresh-air mode. (a) Temperature inside (blue) and outside (red) the cabin; (b) TVOC concentration inside (blue) and outside (red) the cabin; (c) PM2.5 concentration inside (blue) and outside (red) the cabin; (d) power usage of the HVAC system.

Figure 15(a) shows the temperature measured inside and outside the cabin keeping the fresh-air mode. Like for the winter operation, the air temperature inside the cabin is calculated as the mean value of the 18 temperature readings for each timestamp.

Figure 15(b) shows TVOC measured inside and outside the cabin during the fresh-air mode. The figure reports that the TVOC concentration is higher than that of the external air at the beginning, but decreases while the HVAC system operates. The lowest concentration is reached, despite the fluctuation on the outside. This behaviour can be explained with a drop of temperature inside the cabin combined with fresh-air mixing.

Figure 15(c) plots the PM2.5 concentration measured inside and outside the cabin in fresh-air configuration. This result appears to be in contrast with the results shown by [21], *i.e.* the cabin filter is not able to lower the PM concentration inside the car with a steady state filtration efficiency $\eta = 0$ to 0.2. Here we need to consider that the value of PM2.5 concentrations measured were very low and under the sensor precision for that particle size range ($\pm 10\,\mu g/m^3$).

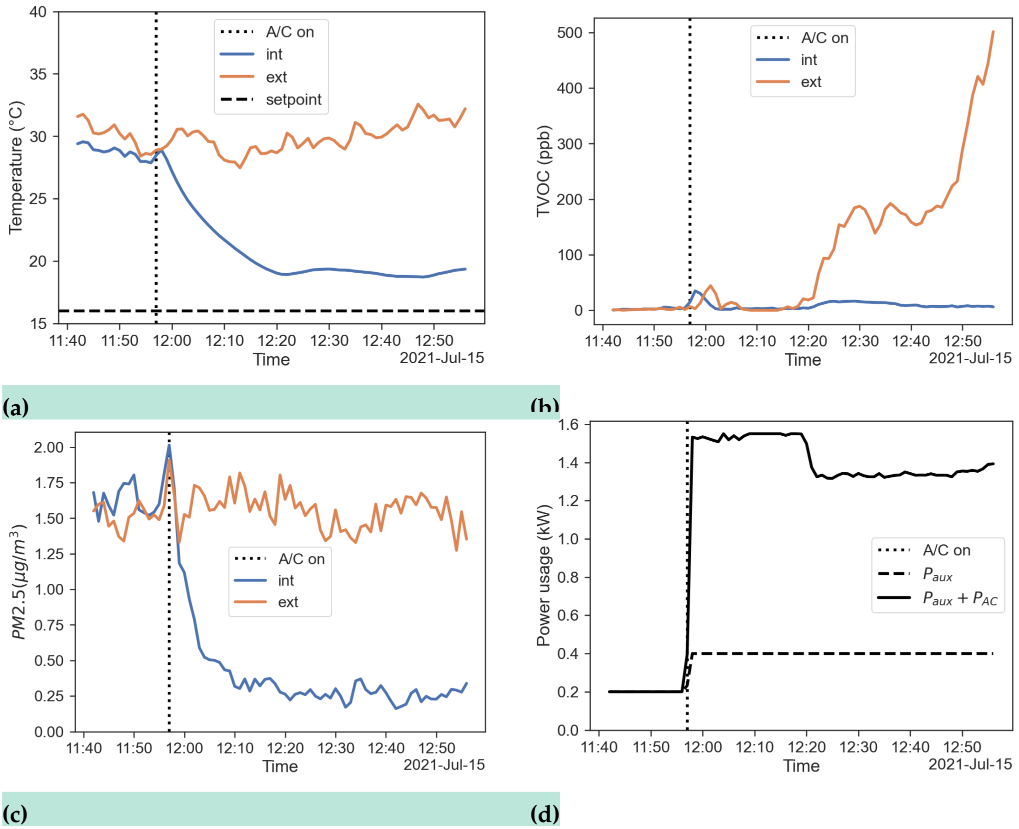

Figure 8. Results regarding the recirculation mode. (a) Temperature inside (blue) and outside (red) the cabin; (b) TVOC concentration inside (blue) and outside (red) the cabin; (c), PM2.5 concentration inside (blue) and outside (red) the cabin; (d) power usage of the HVAC system.

experiments, a temperature adimensionalisation is performed according to equation 1. Figure 9(a) shows a comparison between the dimensionless temperatures obtained for the two experiments. It is noticeable that the value of dimensionless temperature obtained with recirculation is always higher than the one obtained in the fresh-air case, thus suggesting that the cabin approaches better the set-pot in the first case. The filtration efficiency of the vehicle can be defined again using a black box approach, where the vehicle cabin is considered as a system with an unknown filtration capacity, while inlet (external) and outlet (internal) concentrations are known, according to equation 2. Figure 9(b) shows a comparison between the two cases. The PM filtration efficiency with recirculation mode is well over the one with the fresh-air mode. It is also noticeable that as for the winter case the filtration efficiency does never reach the ideal value of $\eta = 1$, but it is even lower indeed. This trend can be explained with η being function of particle size[10], but also of particle concentration itself. As shown for the winter case, another way to investigate cabin performance on airborne pollutants is provided by equation 3. Figure 10 reports the CAQI trend for PM2.5 and TVOC in fresh-air and recirculation, the latter being less prone to build up of pollutants during operation. Figure 11 shows the comparison between the cumulative energy consumption in the two cases of recirculation on and off, calculated as the approximate cumulative integral of $P_{tot} = P_{aux} + P_{AC}$ via the trapezoidal method as done in the winter case with equation 4. The total energy consumption obtained in the recirculation mode on is about $4/5$ of the value obtained for the fresh-air mode. This result can explain what is shown in figures 7(d) and 8(d). These figures show that the power usage from the HVAC system is similar for the two modes in the first minutes of operation. However, when the effects of recirculation become prevalent, the HVAC power usage related to this mode decreases after about 20 min, even though the difference is less prominent than in winter operation.

3.2.2. Recirculation mode

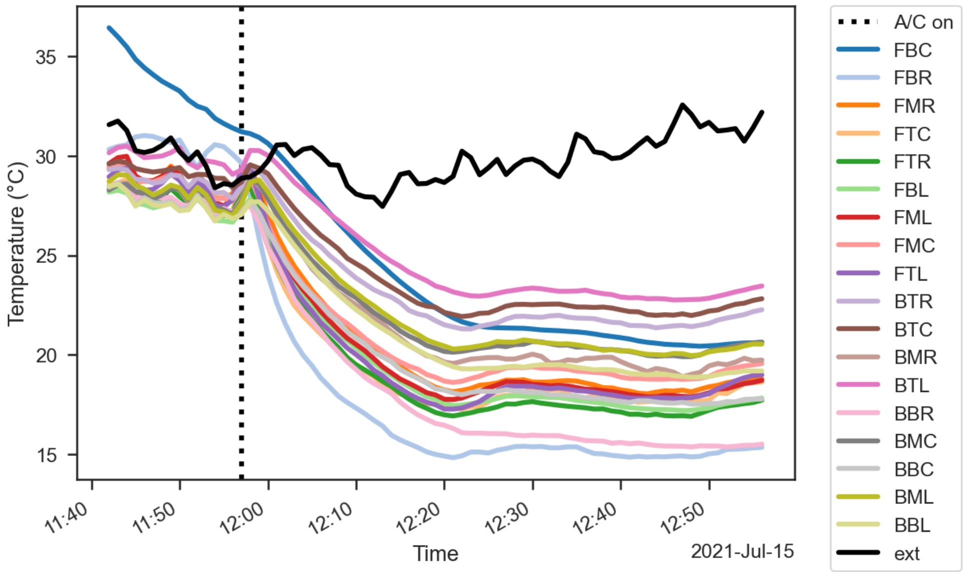

Figure 16. Cooling temperature profiles, recirculation mode.

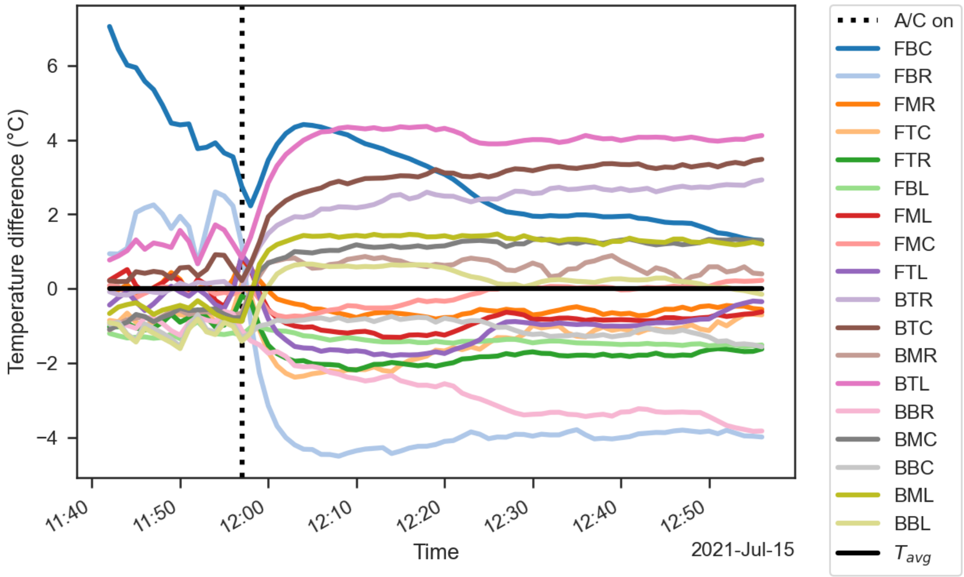

Figure 17. Cooling temperature difference profiles, recirculation mode.

Figure 16 shows the readings from all the 18 temperature sensors in the cabin for the case with recirculation mode. Air temperature stratification with respect to the vertical axis is again considerable, colder spots can be found at feet level of the back seats. The main difference with the fresh-air case is that bottom right location are well capable of reaching the set-point temperature.

As for the previous case with recirculation activated, time to steady state is close to 20 min of operation, while the over-temperature issue is still significant. TVOC concentration show a totally different trend. Despite external concentration peaks at the end of the test, the internal one remains quite low. As for PM values, they follow a completely different trend, it is pretty clear from Fig. 18(c) how the filtration performance is improved by the recirculation mode, even with absolute values well within the precision range as is the previous test.

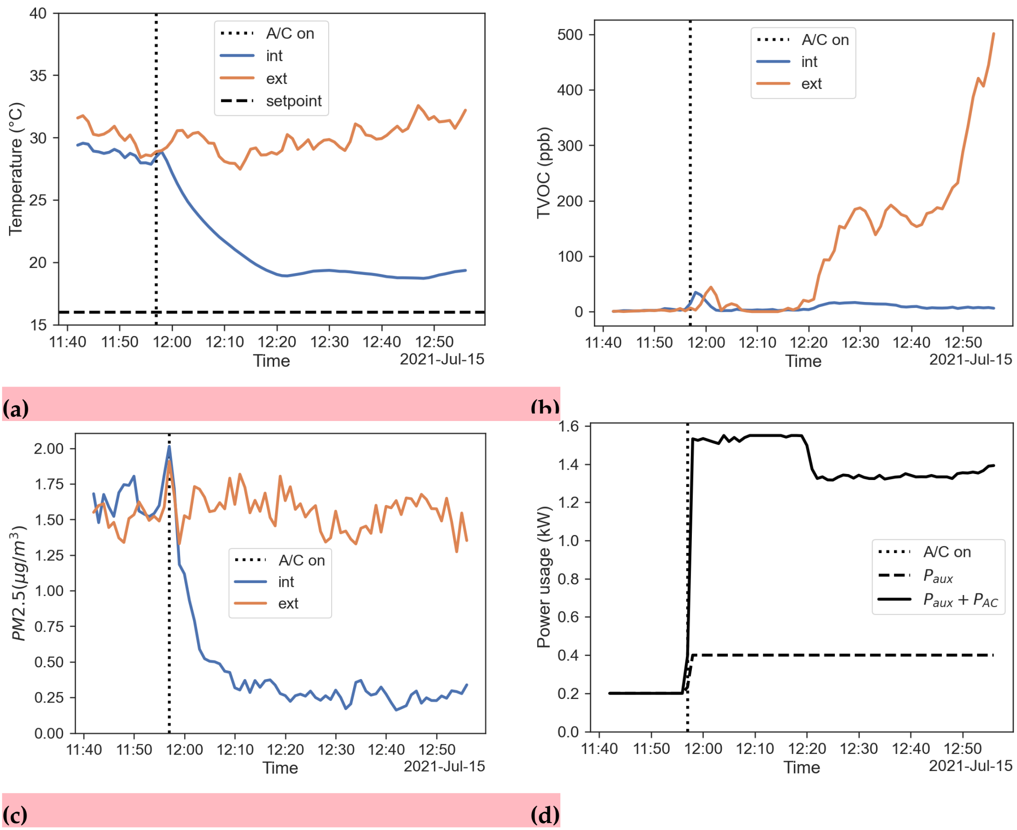

Figure 18. Results regarding the recirculation mode. (a) Temperature inside (blue) and outside (red) the cabin; (b) TVOC concentration inside (blue) and outside (red) the cabin; (c), PM2.5 concentration inside (blue) and outside (red) the cabin; (d) power usage of the HVAC system.

3.2.3. Comparison between fresh-air and recirculation modes in summer

Experiments in cooling as well as heating mode have been performed in real parking conditions with no control on the environment outside the cabin, repeatability issues are worsen by the increased contribution of solar load in summer. In order to compare the experiments, a temperature adimensionalisation is performed according to equation 1. Figure 19 shows a comparison between the dimensionless temperatures obtained for the two experiments. It is noticeable that the value of dimensionless temperature

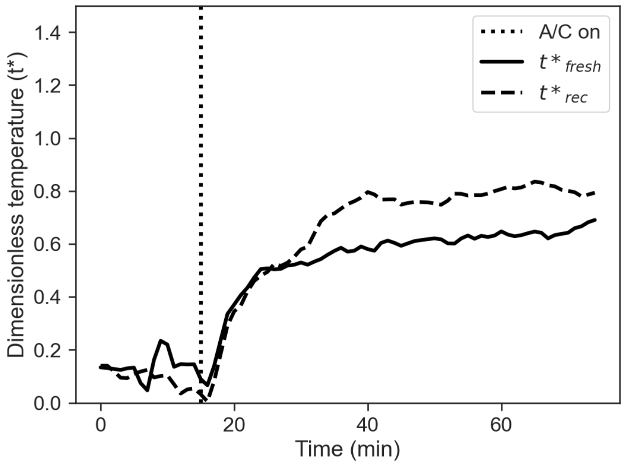

Figure 19. Dimensionless temperature profiles, comparison between fresh-air (solid line) and recirculation (dashed line) mode.

obtained with recirculation is always higher than the one obtained in the fresh-air case, thus suggesting that the cabin approaches better the set-pot in the first case.

The filtration efficiency of the vehicle can be defined again using a black box approach, where the vehicle cabin is considered as a system with an unknown filtration capacity, while inlet (external) and outlet (internal) concentrations are known, according to equation 2. Figure 20 shows a comparison between the two cases. The PM filtration efficiency with recirculation mode is well over the one with the fresh-air mode. It is also noticeable that as for the winter case the filtration efficiency does never reach the ideal value of $\eta = 1$, but it is even lower indeed. This trend can be explained with η being function of particle size[21], but also of particle concentration itself.

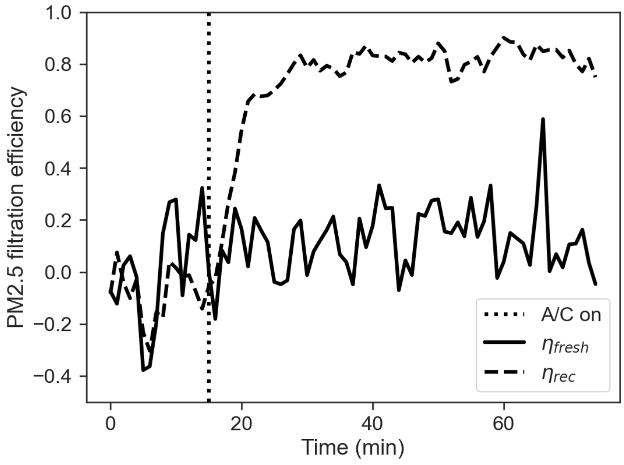

Figure 20. Filtration efficiency, comparison between fresh-air mode (solid line) and recirculation (dashed line) mode.

As shown for the winter case, another way to investigate cabin performance on airborne pollutants is provided by equation 3. Figures 21 and 22 report the CAQI trend

for PM2.5 and TVOC in fresh-air and recirculation, the latter being less prone to build up of pollutants during operation.

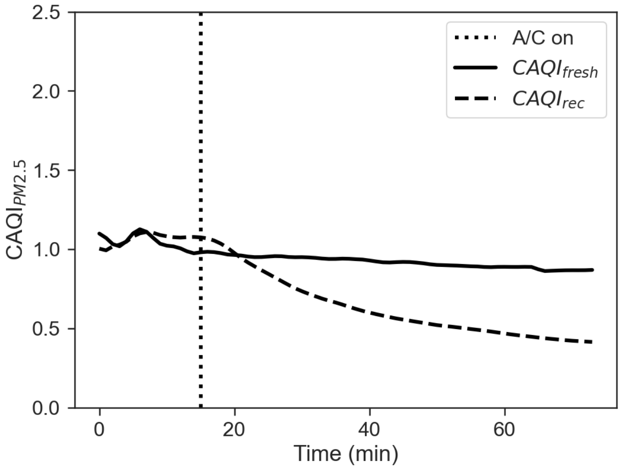

Figure 21. CAQI for PM2.5.

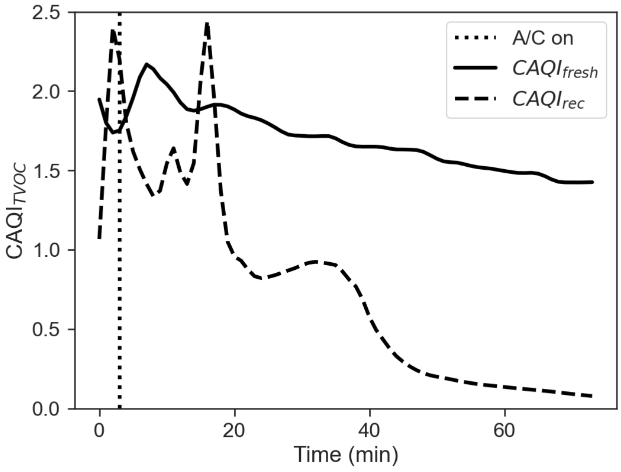

Figure 22. CAQI for TVOC.

Figure 23 shows the comparison between the cumulative energy consumption in the two cases of recirculation on and off, calculated as the approximate cumulative integral of $P_{tot} = P_{aux} + P_{AC}$ via the trapezoidal method as done in the winter case with equation 4. The total energy consumption obtained in the recirculation mode on is about $4/5$ of the value obtained for the fresh-air mode. This result can explain what is shown in figures 15(d) and 18(d). This figures show that the power usage from the HVAC system is similar for the two modes in the first minutes of operation. However, when the effects of recirculation become prevalent, the HVAC power usage related to this mode becomes lower after about 20 min, even though the difference is less prominent then in the winter operation.

3.3. *Effect of filter conditions on filtration performance*

In this section we report some results regarding the filtration performances of the Leaf cabin filter (Figure 24). In detail we performed two tests in summer operating conditions manipulating the filter. First, we performed the experiment without the filter. Then, a second experiment was performed after installing a new brand filter. Figure 24(a) shows that the presence of the filter has an effect of filtration efficiency, lowering its value from 80 % to 60 %. The figure shows that even without a filter the recirculation

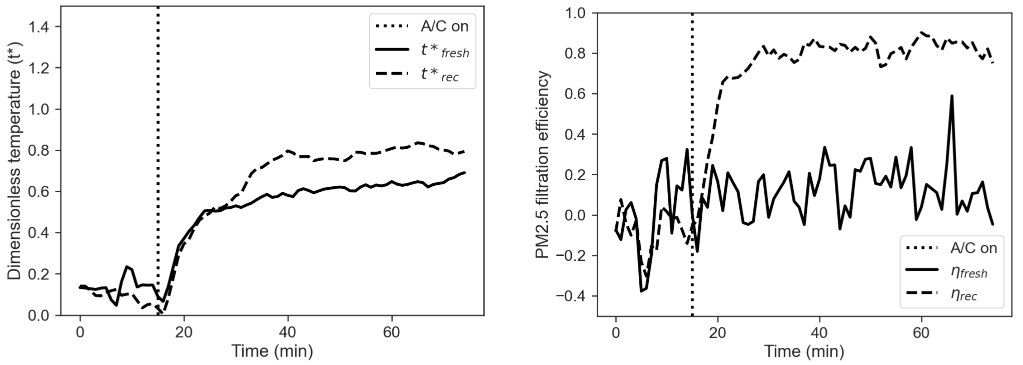

(a) **(b)**

Figure 9. (a) Dimensionless temperature profiles, comparison between fresh-air (solid line) and recirculation (dashed line) mode. (b) Filtration efficiency, comparison between fresh-air (solid line) and recirculation mode (dashed line).

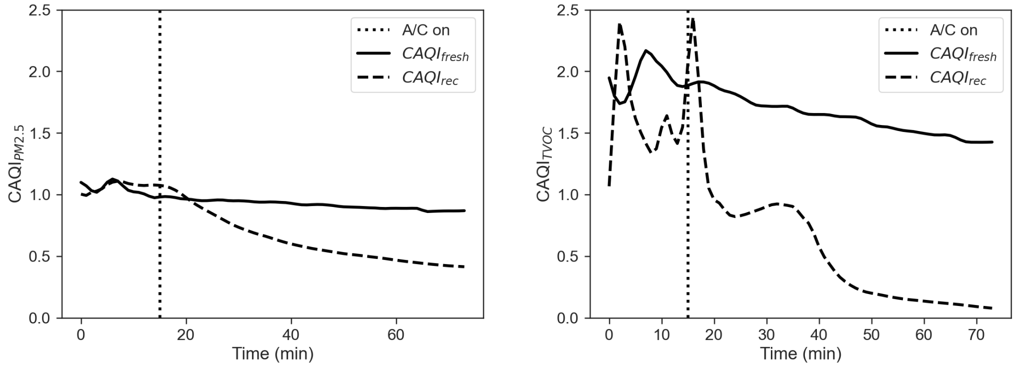

(a) **(b)**

Figure 10. (a) CAQI for PM2.5 and (b) CAQI for TVOC.

3.3. Effect of filter conditions on filtration performance

In this section we report some results regarding the filtration performances of the Leaf cabin filter (Figure 12). In detail we performed two tests in summer operating conditions manipulating the filter. First, we performed the experiment without the filter. Then, a second experiment was performed after installing a brand-new filter. Figure 12(a) shows that the presence of the filter has an effect of filtration efficiency, lowering its value from 80 % to 60 %. The figure shows that even without a filter the recirculation mode provides a sort of filtration. This result suggests that part of the filtration is made by the filter and part is given by other devices in the HVAC system, *i.e.* a fraction of the pollutants is captured by the evaporator fins, or by the ducts between the cabin and the evaporator. Curves for fresh-air mode show filtration efficiency around zero for both the cases (no filter and with a filter), with more fluctuations for the case without a filter. Then, the presence of the filter does not improve the air quality within the cabin both by using a filter and by not using it.

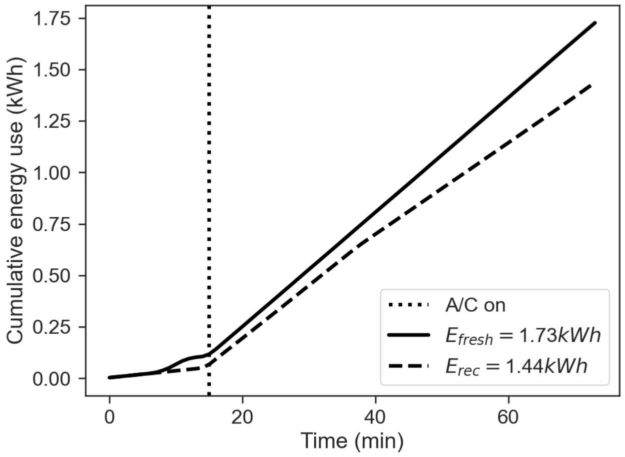

Figure 23. Cumulative energy use, comparison between fresh-air mode (solid line) and recirculation (dashed line) mode.

mode provides a sort of filtration. This result suggests that part of the filtration is made by the filter and part is given by other devices in the HVAC system, *i.e.* a fraction of the pollutants is captured by the evaporator fins, or by the ducts between the cabin and the evaporator. Curves for fresh-air mode show filtration efficiency around zero for both the cases (no filter and with a filter), with more fluctuations for the case without a filter. Then, the presence of the filter does not improve the air quality within the cabin both by using a filter and by not using it.

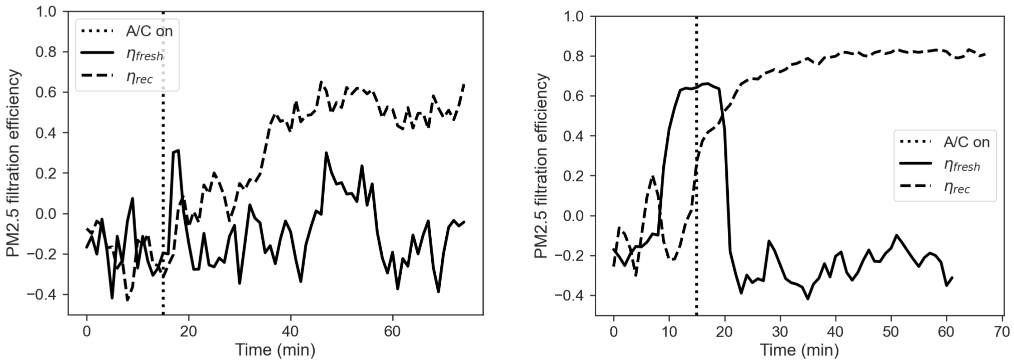

(a) **(b)**

Figure 24. Filtration efficiency, comparison between fresh-air mode (solid line) and recirculation (dashed line) mode, with 24(a) no cabin filter installed and 24(b) with brand new cabin filter installed.

4. Discussion

In this paper, a characterisation of the air quality within the cabin of a battery electric vehicle (BEV) has been shown. The temperature, PM and VOC concentrations have been measured by means of a low-cost Arduino-based system of sensors. Comparisons between the air quality obtained in the cabin during different configuration modes of the air-ventilation system have been carried out.

The main differences outlined are related to the fresh-air and recirculation configurations. The temperature distributions have been monitored and analysed along with the energy consumption of the HVAC system. The results show that, while PMs are filtered, VOCs concentrations tend to increase during operation in recirculation mode. At the same time, the HVAC energy consumption in recirculation mode after 1 h of operation is about 72 % and 82 % of the energy consumption observed in fresh-air mode for heating

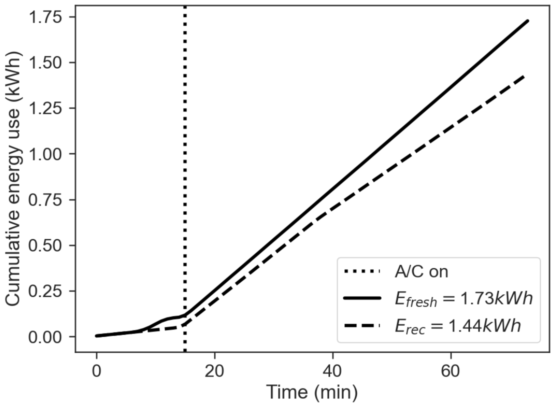

Figure 11. Cumulative energy use, comparison between fresh-air mode (solid line) and recirculation (dashed line) mode.

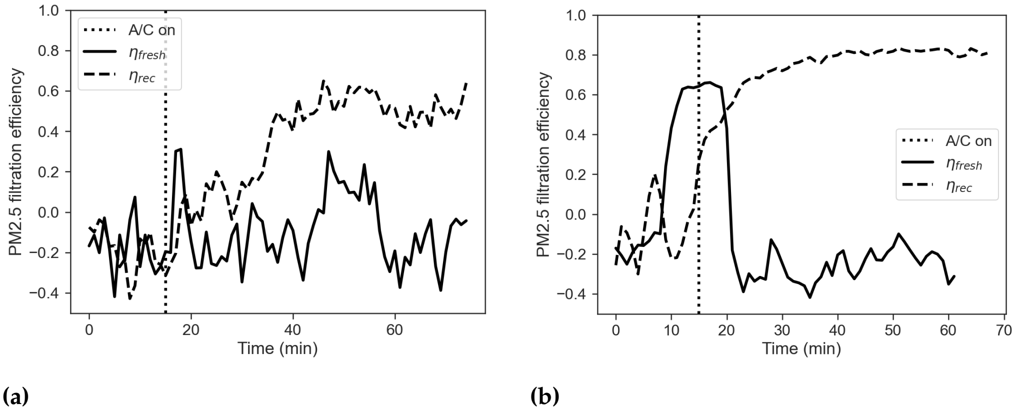

Figure 12. Filtration efficiency, comparison between fresh-air mode (solid line) and recirculation (dashed line) mode, with 12(a) no cabin filter installed and 12(b) with brand new cabin filter installed.

4. Conclusions

In this paper, a characterisation of the air quality within the cabin of a battery electric vehicle (BEV), together with real time measurements of HVAC system energy consumption has been presented. The temperature, PM, and VOC concentrations have been measured by means of a low-cost Arduino-based system of sensors. Comparisons between the air quality obtained in the cabin during different configuration modes of the air-ventilation system have been carried out.

The results show that, while PMs are filtered, VOCs concentrations increase during operation in recirculation mode. At the same time, the HVAC energy consumption in recirculation mode is about 70 % of the energy consumption measured in fresh-air mode during heating operation. In cooling operation, the HVAC energy consumption in recirculation mode is about 80 % of the energy consumption measured in fresh-air mode.

Recirculation mode is found to be the best choice for BEVs, both for reducing some pollutants concentrations and for saving energy. The use of a new filter can improve the filtration efficiency in recirculation mode.

The methodology presented in this paper, applied to a Nissan Leaf Acenta 40 kWh, can be easily extended to other vehicles. This approach is very important for BEVs as the parameters analysed are crucial for these vehicles. In fact, the air quality is strongly related to air-circulation modes, such as the fresh-air or recirculation modes. The recirculation mode should be chosen for energy saving in order to extend the BEV drive range, but a fresh-air mode is needed in some cases, to ensure low concentrations

and cooling operation respectively. Moreover, cabin over temperature issues are observed in both fresh-air and recirculation mode. This result, despite being consistent in terms temperature different, has an opposite impact on system performance depending on the season considered; in the winter case a temperature higher than the set-point might be interpreted as a waste of energy and could be avoided, in the summer case a higher than set-point temperature might suggest that the HVAC system is undersized for cooling operation.

Temperature gradients inside the cabin pose also questions about the correct location of the measurement point. On one hand, discrepancies are significant and can approach 15 C; on the other hand, their uniform behaviour after the startup phase could suggest that a reduction of the measuring points could be attempted without compromising spatio-temporal resolution.

Thermal aspects are crucial for determining the energy consumed by the inbuilt climate control system outside, and outside air temperature is not the only parameter of interest; also solar irradiance and wind speed have an impact on cabin temperature. Future studies must address also these quantities to possibly obtain a proper model of the vehicle cabin.

Recirculation mode remains the best choice for BEVs both for reducing some pollutants concentrations and for saving energy. The use of a new filter can improve the filtration efficiency in recirculation mode, while does not make difference in fresh-air mode. These results need to be compared with the CO2 concentrations obtained in recirculation mode, in order to find the minimum energy consumption together with the best air quality within the cabin in a win-win approach. An optimal data-driven duty-cycle of recirculation activation and deactivation could be defined to optimize both comfort needs and energy consumption.

Author Contributions: Individual author contributions to the present research paper can be summarized as follows: Conceptualization, L.R., B.P. ,G.S. and P.G; methodology, L.R. and P.G.; software, P.G., L.R and D.A.; validation, B.P., G.S. and G.P.; formal analysis, L.R. and P.G.; investigation, L.R.; resources, P.G., B.P., G.S, and G.P.; data curation, L.R. and P.G.; writing—original draft preparation, L.R., P.G. and D.A.; writing—review and editing, B.P., G.S. and G.P.; visualization, L.R.; supervision, B.P. and G.S.; project administration, B.P.; funding acquisition, B.P. All authors have read and agreed to the published version of the manuscript.

Funding: This work has received funding from the LiBER project, funded by Emilia-Romagna region (Italy), POR-FESR 2014-2020.

Acknowledgments: The Authors want to thank Maurizio Chendi for the precious help in the set-up of the experiments.

Conflicts of Interest: The authors declare no conflict of interest. The funders had no role in the design of the study; in the collection, analyses, or interpretation of data; in the writing of the manuscript, or in the decision to publish the results.

Abbreviations

The following abbreviations are used in this manuscript:

of pollutants within the cabin. Control systems should consider these results in order to manage the HVAC system operation in a win-win approach for BEVs.

Supplementary Materials: The following are available online at https://www.mdpi.com/1424-8220/1/1/0/s1, Figure S1:Heating temperature profiles, fresh-air mode, Figure S2:Heating temperature difference profiles, fresh-air mode, Figure S3:Heating temperature profiles, recirculation mode, Figure S4:Heating temperature difference profiles, recirculation mode, Figure S5:Cooling temperature profiles, fresh-air mode, Figure S6:Cooling temperature difference profiles, fresh-air mode, Figure S7:Cooling temperature profiles, recirculation mode, Figure S8:Cooling temperature difference profiles, recirculation mode.

Author Contributions: Individual author contributions to the present research paper can be summarized as follows: Conceptualization, L.R., B.P. ,G.S. and P.G; methodology, L.R. and P.G.; software, P.G., L.R and D.A.; validation, B.P., G.S. and G.P.; formal analysis, L.R. and P.G.; investigation, L.R.; resources, P.G., B.P., G.S, and G.P.; data curation, L.R. and P.G.; writing—original draft preparation, L.R., P.G. and D.A.; writing—review and editing, B.P., G.S. and G.P.; visualization, L.R.; supervision, B.P. and G.S.; project administration, B.P.; funding acquisition, B.P. All authors have read and agreed to the published version of the manuscript.

Funding: This work has received funding from the LiBER project, funded by Emilia-Romagna region (Italy), POR-FESR 2018-2020.

Acknowledgments: The Authors want to thank Maurizio Chendi for the precious help in the set-up of the experiments.

Conflicts of Interest: The authors declare no conflict of interest. The funders had no role in the design of the study; in the collection, analyses, or interpretation of data; in the writing of the manuscript, or in the decision to publish the results.

Abbreviations

The following abbreviations are used in this manuscript:

BEV	Battery Electric Vehicle
HVAC	Heating, Ventilation and Air Conditioning
A/C	Air Conditioning
PTC	Positive Temperature Coefficient
IAQ	Internal Air Quality
OBD	On-Board Diagnostic
MY	Model Year
I/O	Inside/Outside
NTC	Negative Temperature Coefficient
PM	Particulate Matter
VOC	Volatile Organic Compounds

References

1. Russi, L.; Guidorzi, P.; Pulvirenti, B.; Semprini, G.; Aguiari, D.; Pau, G. Air quality and comfort characterisation within an electric vehicle cabin. 2021 IEEE International Workshop on Metrology for Automotive (MetroAutomotive), 2021, pp. 169–174. doi:10.1109/MetroAutomotive50197.2021.9502853.
2. Optemus - Optemus | Optimised and Systematic Energy Management in Electric Vehicles. http://www.optemus.eu/.
3. ISO. *BS ISO/DIS 14505-4:2020. Ergonomics of the thermal environment – Evaluation of thermal environments in vehicles – Part 4: Determination of the equivalent temperature by means of a numerical manikin*; BSI Standards Publication, 2020.
4. Nilsson, H. Comfort Climate Evaluation with Thermal Manikin Methods and Computer Simulation Models. PhD thesis, Byggvetenskap, 2004.
5. Zhang, C.; Shen, K.; Yang, F.; Yuan, C. Multiphysics Modeling of Energy Intensity and Energy Efficiency of Electric Vehicle Operation. *Procedia CIRP* **2019**, *80*, 322–327. doi:10.1016/j.procir.2019.01.058.

BEV	Battery Electric Vehicle
HVAC	Heating, Ventilation and Air Conditioning
IAQ	Internal Air Quality
OBD	On-Board Diagnostic
NTC	Negative Temperature Coefficient
PM	Particulate Matter
VOC	Volatile Organic Compounds
FTL	Front Top Left
FTC	Front Top Center
FTR	Front Top Right
FML	Front Middle Left
FMC	Front Middle Center
FMR	Front Middle Right
FBL	Front Bottom Left
FBC	Front Bottom Center
FBR	Front Bottom Right
BTL	Back Top Left
BTC	Back Top Center
BTR	Back Top Right
BML	Back Middle Left
BMC	Back Middle Center
BMR	Back Middle Right
BBL	Back Bottom Left
BBC	Back Bottom Center
BBR	Back Bottom Right

References

1. Russi, L.; Guidorzi, P.; Pulvirenti, B.; Semprini, G.; Aguiari, D.; Pau, G. Air quality and comfort characterisation within an electric vehicle cabin. 2021 IEEE International Workshop on Metrology for Automotive (MetroAutomotive), 2021, pp. 169–174. doi:10.1109/MetroAutomotive50197.2021.9502853.
2. Atkinson, W.J.; Hill, W.R.; Mathur, G.D. The Impact of Increased Air Recirculation on Interior Cabin Air Quality. SAE Technical Paper. SAE International, 2017. doi:10.4271/2017-01-0169.
3. Pham, L.; Molden, N.; Boyle, S.; Johnson, K.; Jung, H. Development of a Standard Testing Method for Vehicle Cabin Air Quality Index. *SAE International Journal of Commercial Vehicles* **2019**, *12*. doi:10.4271/02-12-02-0012.
4. M. Gerboles.; L. Spinelle.; A. Borowiak. Measuring air pollution with low-cost sensors. https://ec.europa.eu/jrc/en/publication/brochures-leaflets/measuring-air-pollution-low-cost-sensors, accessed on 2021-03-05.
5. Karami, M.; McMorrow, G.V.; Wang, L. Continuous monitoring of indoor environmental quality using an Arduino-based data acquisition system. *Journal of Building Engineering* **2018**, *19*, 412–419. doi:10.1016/j.jobe.2018.05.014.
6. Kumar Sai, K.B.; Mukherjee, S.; Parveen Sultana, H. Low Cost IoT Based Air Quality Monitoring Setup Using Arduino and MQ Series Sensors With Dataset Analysis. *Procedia Computer Science* **2019**, *165*, 322–327. doi:10.1016/j.procs.2020.01.043.
7. Doyle, A.; Muneer, T. Energy consumption and modelling of the climate control system in the electric vehicle. *Energy Exploration & Exploitation* **2019**, *37*, 519–543. Publisher: SAGE Publications Ltd STM, doi:10.1177/0144598718806458.
8. Doyle, A.; Muneer, T.; Smith, I. A review of the thermal performance of electric vehicles. 2015 IEEE International Transportation Electrification Conference (ITEC), 2015, pp. 1–5. ISSN: null, doi:10.1109/ITEC-India.2015.7386922.
9. Koritsoglou, K.; Christou, V.; Ntritsos, G.; Tsoumanis, G.; Tsipouras, M.G.; Giannakeas, N.; Tzallas, A.T. Improving the Accuracy of Low-Cost Sensor Measurements for Freezer Automation. *Sensors* **2020**, *20*, 6389. Number: 21 Publisher: Multidisciplinary Digital Publishing Institute, doi:10.3390/s20216389.
10. ISO. *BS ISO 16000-37:2019. Indoor Air - Part 37: Measurement of $PM_{2.5}$ mass concentration*; BSI Standards Publication, 2019.

6. Organization, W.H., Ed. *WHO guidelines for indoor air quality: household fuel combustion*; World Health Organization: Geneva, Switzerland, 2014. OCLC: ocn927460179.
7. Organization, W.H., Ed. *Who guidelines for indoor air quality: selected pollutants*; WHO: Copenhagen, 2010. OCLC: ocn696099951.
8. Heseltine, E.; Rosen, J.; Organization, W.H., Eds. *WHO guidelines for indoor air quality: dampness and mould*; WHO: Copenhagen, 2009. OCLC: ocn429024432.
9. Settimo, G.; Manigrasso, M.; Avino, P. Indoor Air Quality: A Focus on the European Legislation and State-of-the-Art Research in Italy. *Atmosphere* **2020**, *11*, 370. Number: 4 Publisher: Multidisciplinary Digital Publishing Institute, doi:10.3390/atmos11040370.
10. Heo, K.J.; Noh, J.W.; Lee, B.U.; Kim, Y.; Jung, J.H. Comparison of filtration performance of commercially available automotive cabin air filters against various airborne pollutants. *Building and Environment* **2019**, *161*, 106272. doi:10.1016/j.buildenv.2019.106272.
11. Atkinson, W.J.; Hill, W.R.; Mathur, G.D. The Impact of Increased Air Recirculation on Interior Cabin Air Quality. SAE Technical Paper. SAE International, 2017. doi:10.4271/2017-01-0169.
12. Vartires, A.; Colda, I.; Toropoc, S.M. Research on thermal comfort and indoor air pollution in a passenger car. 2017 International Conference on ENERGY and ENVIRONMENT (CIEM), 2017, pp. 148–152. doi:10.1109/CIEM.2017.8120872.
13. Pham, L.; Molden, N.; Boyle, S.; Johnson, K.; Jung, H. Development of a Standard Testing Method for Vehicle Cabin Air Quality Index. *SAE International Journal of Commercial Vehicles* **2019**, *12*. doi:10.4271/02-12-02-0012.
14. Grady, M.L.; Jung, H.; chul Kim, Y.; Park, J.K.; Lee, B.C. Vehicle Cabin Air Quality with Fractional Air Recirculation. SAE 2013 World Congress & Exhibition, 2013, pp. 2013–01–1494. doi:10.4271/2013-01-1494.
15. Alger, J.A.; Buss, A.R.; Creasman, J.D.; Hoy, J.R. Automatic vehicle climate control based on predicted air quality. US US10226982B2, March, 2019.
16. NEWMAN, A. Automatic vehicle cabin air filtration system. US US10245924B2, April, 2019.
17. Li, C.; Brewer, E.; Pham, L.; Jung, H. Reducing Mobile Air Conditioner (MAC) Power Consumption Using Active Cabin-Air-Recirculation in A Plug-In Hybrid Electric Vehicle (PHEV). *World Electric Vehicle Journal* **2018**, *9*, 51. Number: 4 Publisher: Multidisciplinary Digital Publishing Institute, doi:10.3390/wevj9040051.
18. M. Gerboles.; L. Spinelle.; A. Borowiak. Measuring air pollution with low-cost sensors. https://ec.europa.eu/jrc/en/publication/brochures-leaflets/measuring-air-pollution-low-cost-sensors, accessed on 2021-03-05.
19. Rüffer, D.; Hoehne, F.; Bühler, J. New Digital Metal-Oxide (MOx) Sensor Platform. *Sensors (Basel, Switzerland)* **2018**, *18*. doi:10.3390/s18041052.
20. Yasuda, T.; Yonemura, S.; Tani, A. Comparison of the Characteristics of Small Commercial NDIR CO2 Sensor Models and Development of a Portable CO2 Measurement Device. *Sensors* **2012**, *12*, 3641–3655. Number: 3 Publisher: Molecular Diversity Preservation International, doi:10.3390/s120303641.
21. Tryner, J.; Mehaffy, J.; Miller-Lionberg, D.; Volckens, J. Effects of aerosol type and simulated aging on performance of low-cost PM sensors. *Journal of Aerosol Science* **2020**, *150*, 105654. doi:10.1016/j.jaerosci.2020.105654.
22. Kondaveeti, H.K.; Kumaravelu, N.K.; Vanambathina, S.D.; Mathe, S.E.; Vappangi, S. A systematic literature review on prototyping with Arduino: Applications, challenges, advantages, and limitations. *Computer Science Review* **2021**, *40*, 100364. doi:10.1016/j.cosrev.2021.100364.
23. Karami, M.; McMorrow, G.V.; Wang, L. Continuous monitoring of indoor environmental quality using an Arduino-based data acquisition system. *Journal of Building Engineering* **2018**, *19*, 412–419. doi:10.1016/j.jobe.2018.05.014.
24. Kumar Sai, K.B.; Mukherjee, S.; Parveen Sultana, H. Low Cost IoT Based Air Quality Monitoring Setup Using Arduino and MQ Series Sensors With Dataset Analysis. *Procedia Computer Science* **2019**, *165*, 322–327. doi:10.1016/j.procs.2020.01.043.
25. Doyle, A.; Muneer, T. Energy consumption and modelling of the climate control system in the electric vehicle. *Energy Exploration & Exploitation* **2019**, *37*, 519–543. Publisher: SAGE Publications Ltd STM, doi:10.1177/0144598718806458.
26. Doyle, A.; Muneer, T.; Smith, I. A review of the thermal performance of electric vehicles. 2015 IEEE International Transportation Electrification Conference (ITEC), 2015, pp. 1–5. ISSN: null, doi:10.1109/ITEC-India.2015.7386922.
27. Koritsoglou, K.; Christou, V.; Ntritsos, G.; Tsoumanis, G.; Tsipouras, M.G.; Giannakeas, N.; Tzallas, A.T. Improving the Accuracy of Low-Cost Sensor Measurements for Freezer

11. ISO. *BS ISO 12219-1:2012. Interior air of road vehicles — Part 1: Whole vehicle test chamber —Specification and method for the determination of volatile organic compounds in cabin interiors*; BSI Standards Publication, 2012.
12. Aguiari, D. Exploring Computing Continuum in IoT Systems: Sensing, Communicating and Processing at the Network Edge. PhD thesis, University of Cagliari - Sorbonne Université, 2021. Cap. 7.
13. Christ, R.D.; Wernli, R.L. Sensor Theory. In *The ROV Manual*; Elsevier, 2014; pp. 297–326. doi:10.1016/B978-0-08-098288-5.00012-9.
14. Guthrie, W.F. NIST/SEMATECH e-Handbook of Statistical Methods (NIST Handbook 151). https://www.itl.nist.gov/div898/handbook/, accessed on 2021-03-18. doi: 10.18434/M32189.
15. Guidelines for Reliable Long Line 1-Wire Networks. https://www.maximintegrated.com/en/design/technical-documents/tutorials/1/148.html, accessed on 2021-10-14.
16. Sensortec, B. BME280 Combined humidity and pressure sensor. *Bosch Sensortec* **2018**.
17. Li, J.; Li, H.; Ma, Y.; Wang, Y.; Abokifa, A.A.; Lu, C.; Biswas, P. Spatiotemporal distribution of indoor particulate matter concentration with a low-cost sensor network. *Building and Environment* **2018**, *127*, 138–147. doi:10.1016/j.buildenv.2017.11.001.
18. Tryner, J.; Mehaffy, J.; Miller-Lionberg, D.; Volckens, J. Effects of aerosol type and simulated aging on performance of low-cost PM sensors. *Journal of Aerosol Science* **2020**, *150*, 105654. doi:10.1016/j.jaerosci.2020.105654.
19. Rüffer, D.; Hoehne, F.; Bühler, J. New Digital Metal-Oxide (MOx) Sensor Platform. *Sensors (Basel, Switzerland)* **2018**, *18*. doi:10.3390/s18041052.
20. Nilsson, H. Comfort Climate Evaluation with Thermal Manikin Methods and Computer Simulation Models. PhD thesis, Byggvetenskap, 2004.
21. Heo, K.J.; Noh, J.W.; Lee, B.U.; Kim, Y.; Jung, J.H. Comparison of filtration performance of commercially available automotive cabin air filters against various airborne pollutants. *Building and Environment* **2019**, *161*, 106272. doi:10.1016/j.buildenv.2019.106272.
22. Kim, Y.c.; Park, J.K.; Lee, B.C.; Grady, M.L.; Jung, H. Vehicle Cabin Air Quality with Fractional Air Recirculation. SAE 2013 World Congress & Exhibition. SAE International, 2013. doi: https://doi.org/10.4271/2013-01-1494.
23. Li, C.; Brewer, E.; Pham, L.; Jung, H. Reducing Mobile Air Conditioner (MAC) Power Consumption Using Active Cabin-Air-Recirculation in A Plug-In Hybrid Electric Vehicle (PHEV). *World Electric Vehicle Journal* **2018**, *9*, 51. Number: 4 Publisher: Multidisciplinary Digital Publishing Institute, doi:10.3390/wevj9040051.
24. NEWMAN, A. Automatic vehicle cabin air filtration system. US US10245924B2, April, 2019.
25. Vartires, A.; Colda, I.; Toropoc, S.M. Research on thermal comfort and indoor air pollution in a passenger car. 2017 International Conference on ENERGY and ENVIRONMENT (CIEM), 2017, pp. 148–152. doi:10.1109/CIEM.2017.8120872.
26. Alger, J.A.; Buss, A.R.; Creasman, J.D.; Hoy, J.R. Automatic vehicle climate control based on predicted air quality. US US10226982B2, March, 2019.
27. Kondaveeti, H.K.; Kumaravelu, N.K.; Vanambathina, S.D.; Mathe, S.E.; Vappangi, S. A systematic literature review on prototyping with Arduino: Applications, challenges, advantages, and limitations. *Computer Science Review* **2021**, *40*, 100364. doi:10.1016/j.cosrev.2021.100364.
28. Yasuda, T.; Yonemura, S.; Tani, A. Comparison of the Characteristics of Small Commercial NDIR CO2 Sensor Models and Development of a Portable CO2 Measurement Device. *Sensors* **2012**, *12*, 3641–3655. Number: 3 Publisher: Molecular Diversity Preservation International, doi:10.3390/s120303641.
29. Organization, W.H., Ed. *Who guidelines for indoor air quality: selected pollutants*; WHO: Copenhagen, 2010. OCLC: ocn696099951.
30. Organization, W.H., Ed. *WHO guidelines for indoor air quality: household fuel combustion*; World Health Organization: Geneva, Switzerland, 2014. OCLC: ocn927460179.
31. Heseltine, E.; Rosen, J.; Organization, W.H., Eds. *WHO guidelines for indoor air quality: dampness and mould*; WHO: Copenhagen, 2009. OCLC: ocn429024432.
32. Settimo, G.; Manigrasso, M.; Avino, P. Indoor Air Quality: A Focus on the European Legislation and State-of-the-Art Research in Italy. *Atmosphere* **2020**, *11*, 370. Number: 4 Publisher: Multidisciplinary Digital Publishing Institute, doi:10.3390/atmos11040370.
33. Zhang, C.; Shen, K.; Yang, F.; Yuan, C. Multiphysics Modeling of Energy Intensity and Energy Efficiency of Electric Vehicle Operation. *Procedia CIRP* **2019**, *80*, 322–327. doi: 10.1016/j.procir.2019.01.058.

Automation. *Sensors* **2020**, *20*, 6389. Number: 21 Publisher: Multidisciplinary Digital Publishing Institute, doi:10.3390/s20216389.

28. ISO. *BS ISO 16000-37:2019. Indoor Air - Part 37: Measurement of $PM_{2,5}$ mass concentration*; BSI Standards Publication, 2019.
29. ISO. *BS ISO 12219-1:2012. Interior air of road vehicles — Part 1: Whole vehicle test chamber —Specification and method for the determination of volatile organic compounds in cabin interiors*; BSI Standards Publication, 2012.
30. Aguiari, D. Exploring Computing Continuum in IoT Systems: Sensing, Communicating and Processing at the Network Edge. PhD thesis, University of Cagliari - Sorbonne Université, 2021. Cap. 7.
31. Christ, R.D.; Wernli, R.L. Sensor Theory. In *The ROV Manual*; Elsevier, 2014; pp. 297–326. doi:10.1016/B978-0-08-098288-5.00012-9.
32. Guthrie, W.F. NIST/SEMATECH e-Handbook of Statistical Methods (NIST Handbook 151). https://www.itl.nist.gov/div898/handbook/, accessed on 2021-03-18. doi: 10.18434/M32189.
33. Guidelines for Reliable Long Line 1-Wire Networks. https://www.maximintegrated.com/en/design/technical-documents/tutorials/1/148.html, accessed on 2021-10-14.
34. Sensortec, B. BME280 Combined humidity and pressure sensor. *Bosch Sensortec* **2018**.
35. Li, J.; Li, H.; Ma, Y.; Wang, Y.; Abokifa, A.A.; Lu, C.; Biswas, P. Spatiotemporal distribution of indoor particulate matter concentration with a low-cost sensor network. *Building and Environment* **2018**, *127*, 138–147. doi:10.1016/j.buildenv.2017.11.001.
