# Supplementary material for: Air Quality and Comfort Characterisation within an Electric Vehicle Cabin in Heating and Cooling Operations
Source: Sensors (Basel). 2022 Jan 11;22(2):543. doi: 10.3390/s22020543 (PMC8778250; doi:10.3390/s22020543)
Supplement: Supplementary file 1 [file sensors-22-00543-s001.zip › sensors-1450505 -supp -for pub-final/cpstwwfqbhbzzcfgfrnmhcvfkjxbdtvz.pdf]

Supplementary Materials: Air Quality and Comfort Characterisation within an Electric Vehicle Cabin in Heating and Cooling Operations

Luigi Russi [1,‡*], Paolo Guidorzi [1], Beatrice Pulvirenti [1], Davide Aguiari [2], Giovanni Pau[21] and Giovanni Semprini [1]

1. High spatial resolution analysis of cabin air temperature profile

1.1. Heating operation

1.1.1. Fresh-air configuration

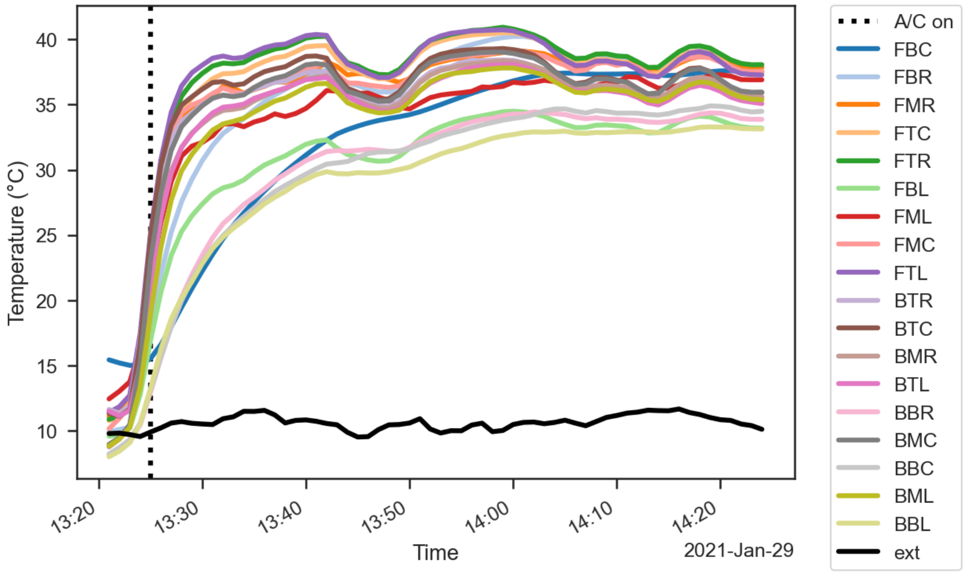

Figure S1. Heating temperature profiles, fresh-air mode.

Figure S1 shows the readings from all the 18 temperature sensors in the cabin for the case with fresh-air mode with a 3 letters naming scheme [1]:

1. The first letter tells us if we are in the front (F) or in the back (B) of the cabin.
2. The second letter refers to the location in vertical direction, namely bottom (B), middle (M) and top (T) plane.
3. The third letter refers to the location in the horizontal direction, namely left (L) or driver, center (C) or right (R) side.

Colder spots can be found at feet level of the back seats, moreover this is in disagreement with the studies of Nilsson on equivalent temperature of body segments [2]. In other words, analysing the results of the work that led to the development of the only available standard for comfort evaluation in vehicles (ISO-14505), we can infer that occupants will be likely to accept colder temperatures in upper body parts during winter, while here we are in the opposite situation. Data from position FBC have a different behaviour because it refers to the sensor installed inside the acquisition system box, thus suffering from thermal inertia issues.

Defining a temperature difference $\Delta t = t_i - t_{avg}$; where t_i is the temperature in one generic position of the grid, and t_{avg} the mean value of the 18 temperature readings for each timestamp; is possible to obtain Figure S2. It is worth noting that air temperature inside the cabin can reach discrepancies of more than 15 °C in the first minutes of operation, while the Δt values at the end of test is lower and the temperature distribution much more uniform.

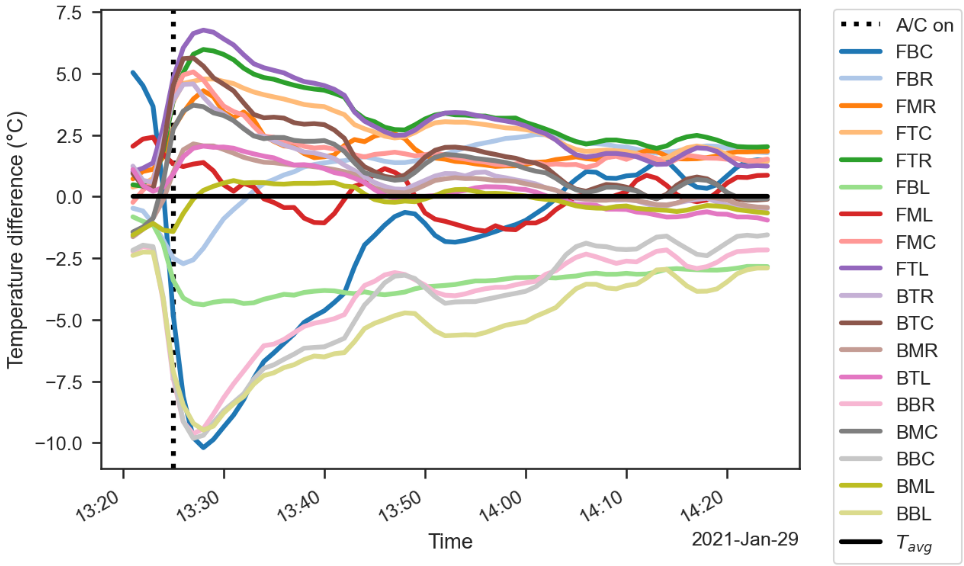

Figure S2. Heating temperature difference profiles, fresh-air mode.

1.1.2. Recirculation configuration

Figure S3 shows the readings from all the 18 temperature sensors in the cabin for the case with recirculation mode. Air temperature inside the cabin shows slightly higher discrepancies with respect to the previous case, colder spots can be found again at feet level of the back seats. Signal from the FBC sensor shows similar responsiveness issues as for the case without recirculation. Higher temperature gradients in the startup phase are evident from Figure S4, while a smoother profile is reached at the end of test.

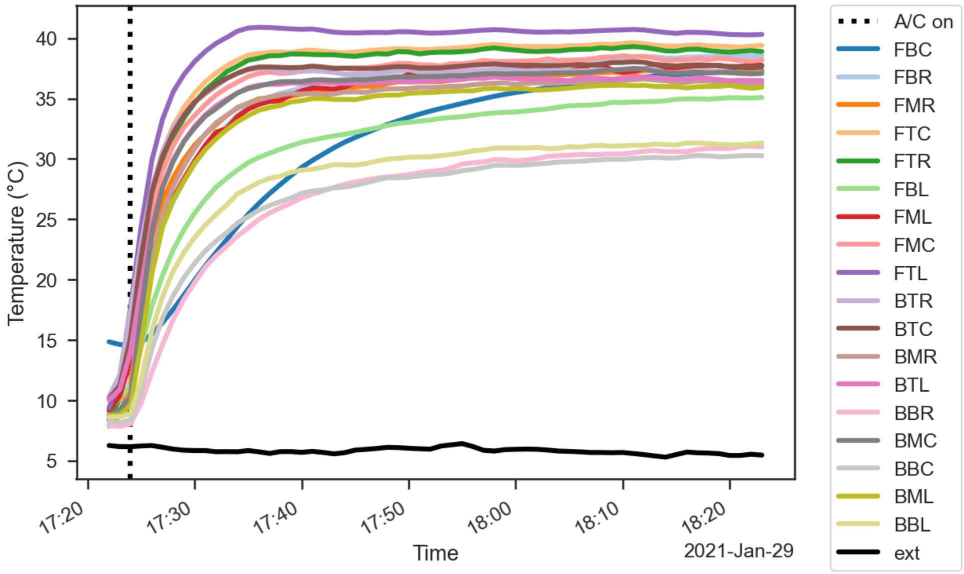

Figure S3. Heating temperature profiles, recirculation mode.

1.2. Cooling operation

1.2.1. Fresh-air configuration

All the experiments confirm that the cooling system is not capable of reaching a quasi-steady state condition in about 60 min, textiti.e. the temperature reached by the air inside the cabin is far from the set-point temperature value. Figure S5 shows the

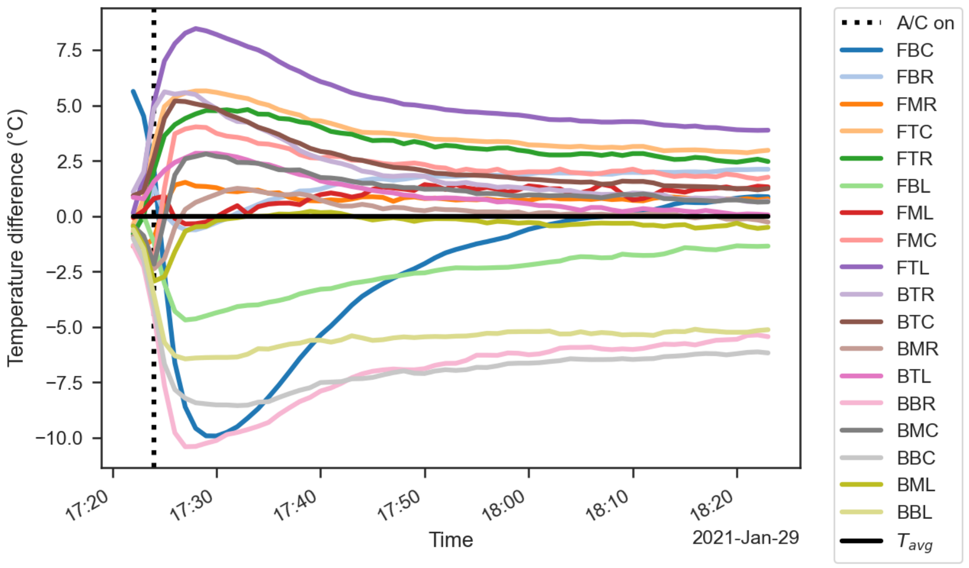

Figure S4. Heating temperature difference profiles, recirculation mode.

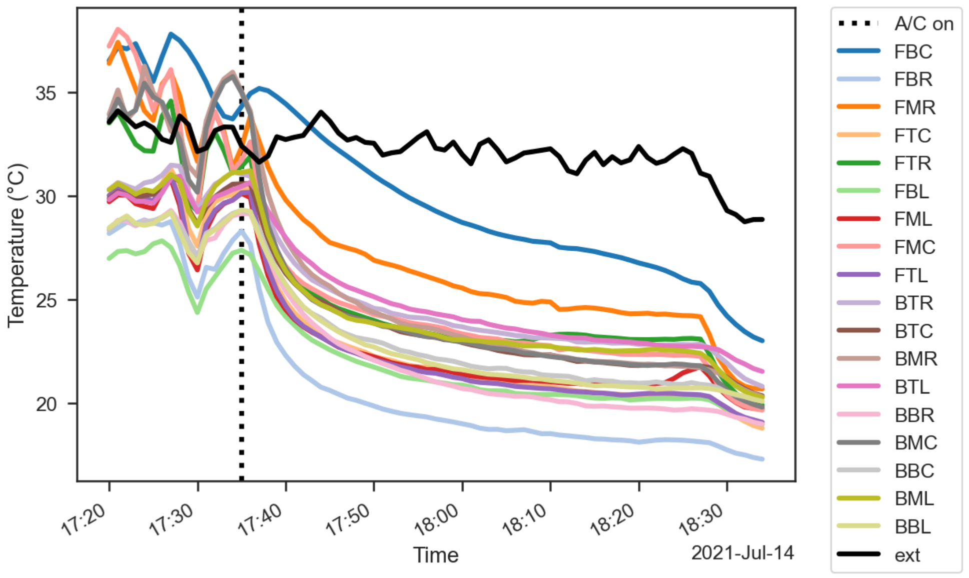

Figure S5. Cooling temperature profiles, fresh-air mode.

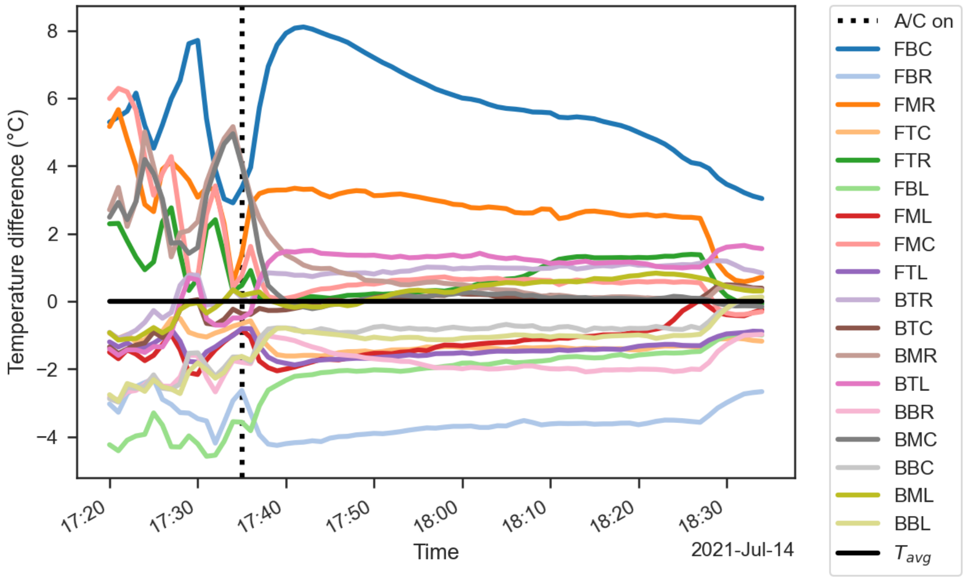

Figure S6. Cooling temperature difference profiles, fresh-air mode.

readings from all the 18 temperature sensors in the cabin for the case with fresh-air mode with the same naming scheme introduced in the previous section.

Temperature discrepancies in the cabin can approach 12 °C; warmer spots can be found at top and middle locations, while bottom zone is generally colder (Fig. S6). To a certain extent, this is in agreement with a stratification of cabin air during operation, despite the vents working at full power. Looking at "Light summer" Comfort Zones defined by [2], we can infer that occupants will be likely to accept warmer temperatures in lower body parts during summer, while this result goes in the opposite direction. Self heating and thermal inertia issue of the sensor positioned at FBC position are still evident.

1.2.2. Recirculation configuration

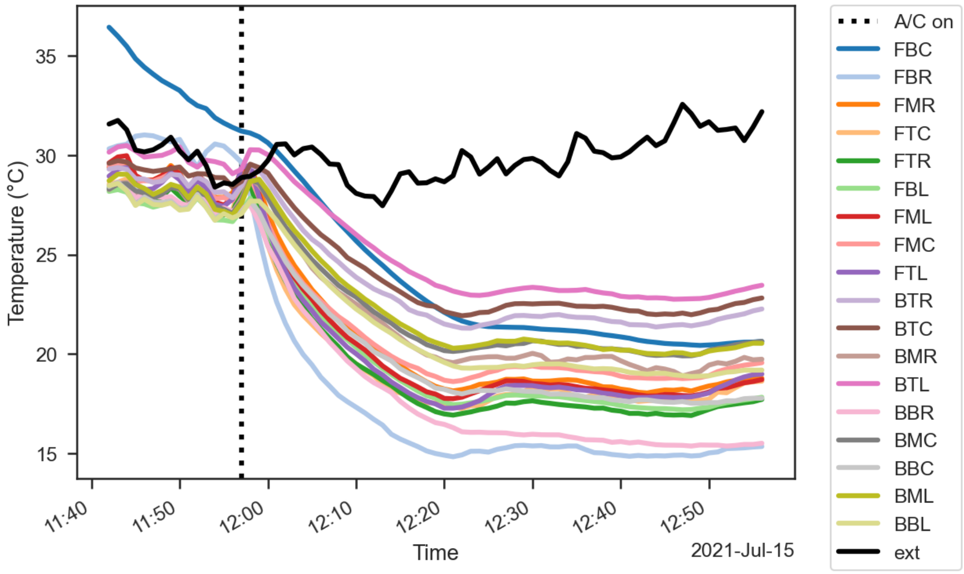

Figure S7. Cooling temperature profiles, recirculation mode.

Figures S7 and S8 show the readings from all the 18 temperature sensors in the cabin for the case with recirculation mode. Air temperature stratification with respect to

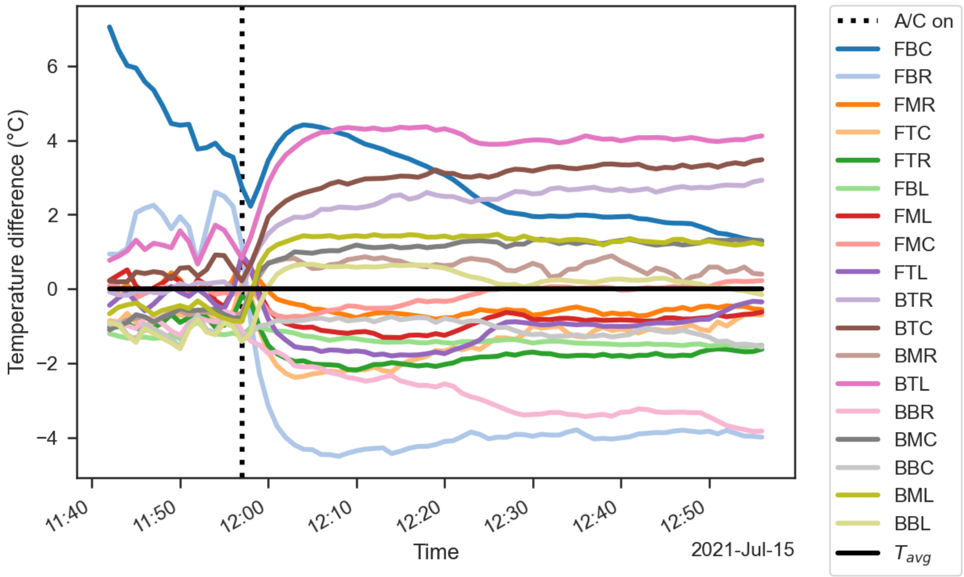

Figure S8. Cooling temperature difference profiles, recirculation mode.

the vertical axis is again considerable, colder spots can be found at feet level of the back seats. The main difference with the fresh-air case is that bottom right location are well capable of reaching the set-point temperature .

Abbreviations

The following abbreviations are used in this document:

FTL	Front Top Left
FTC	Front Top Center
FTR	Front Top Right
FML	Front Middle Left
FMC	Front Middle Center
FMR	Front Middle Right
FBL	Front Bottom Left
FBC	Front Bottom Center
FBR	Front Bottom Right
BTL	Back Top Left
BTC	Back Top Center
BTR	Back Top Right
BML	Back Middle Left
BMC	Back Middle Center
BMR	Back Middle Right
BBL	Back Bottom Left
BBC	Back Bottom Center
BBR	Back Bottom Right

References

1. Russi, L.; Guidorzi, P.; Pulvirenti, B.; Semprini, G.; Aguiari, D.; Pau, G. Air quality and comfort characterisation within an electric vehicle cabin. 2021 IEEE International Workshop on Metrology for Automotive (MetroAutomotive), 2021, pp. 169–174. doi:10.1109/MetroAutomotive50197.2021.9502853.
2. Nilsson, H. Comfort Climate Evaluation with Thermal Manikin Methods and Computer Simulation Models. PhD thesis, Byggvetenskap, 2004.
