# Supplementary figures and images for: Air Quality and Comfort Characterisation within an Electric Vehicle Cabin in Heating and Cooling Operations
Source: Sensors (Basel). 2022 Jan 11;22(2):543. doi: 10.3390/s22020543 (PMC8778250; doi:10.3390/s22020543)

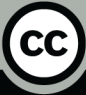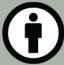

BY

Supplement: Supplementary file 1 [file sensors-22-00543-s001.zip › sensors-1450505 -supp -for pub-final/Definitions/logo-ccby-eps-converted-to.pdf]

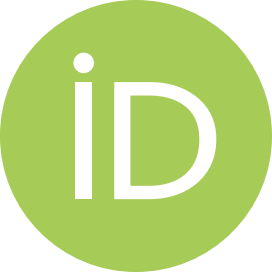

Supplement: Supplementary file 1 [file sensors-22-00543-s001.zip › sensors-1450505 -supp -for pub-final/Definitions/logo-orcid-eps-converted-to.pdf]

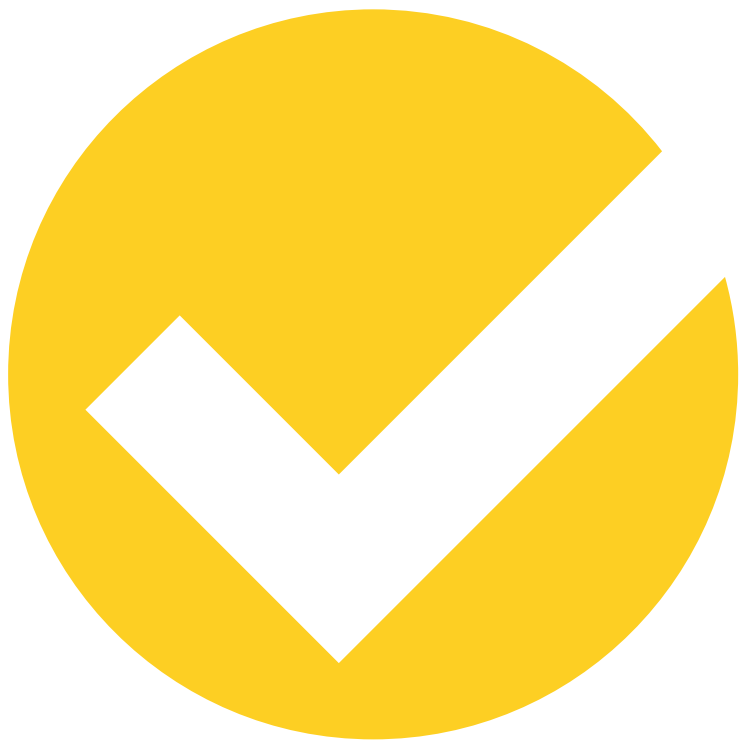

check for  
updates

Supplement: Supplementary file 1 [file sensors-22-00543-s001.zip › sensors-1450505 -supp -for pub-final/Definitions/logo-updates.pdf]

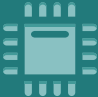

*sensors*

Supplement: Supplementary file 1 [file sensors-22-00543-s001.zip › sensors-1450505 -supp -for pub-final/Definitions/sensors-logo-eps-converted-to.pdf]

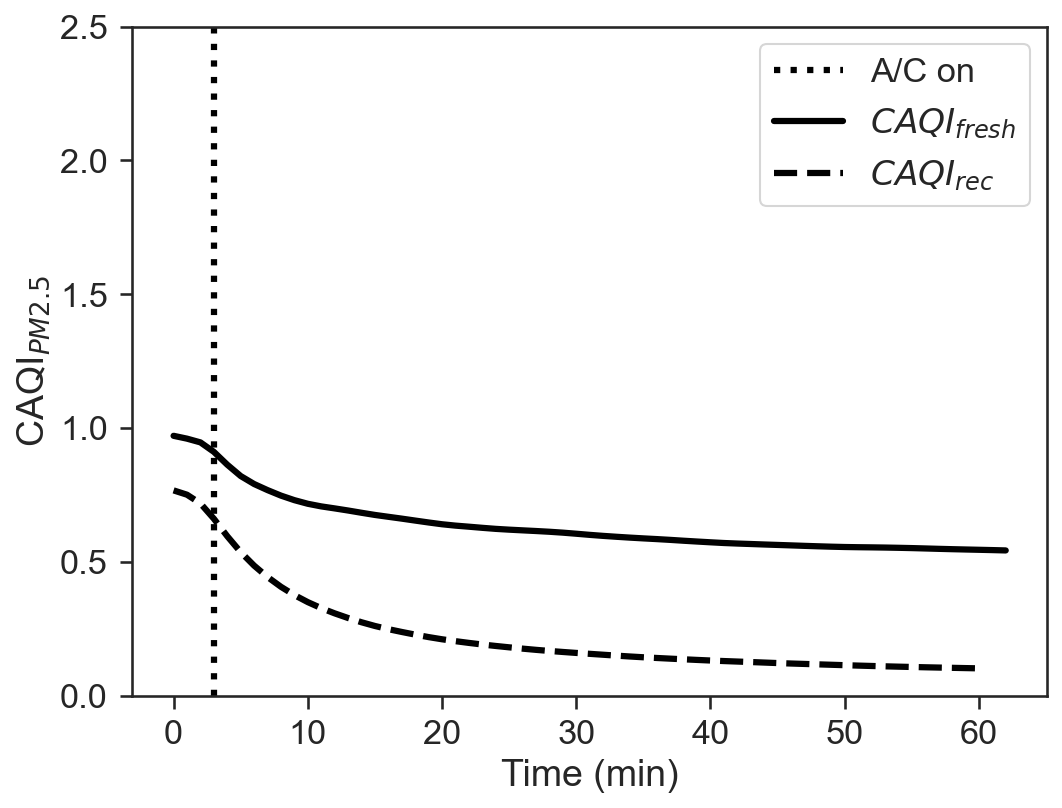

Supplement: Supplementary file 1 [file sensors-22-00543-s001.zip › sensors-1450505 -supp -for pub-final/img/caqi.png]

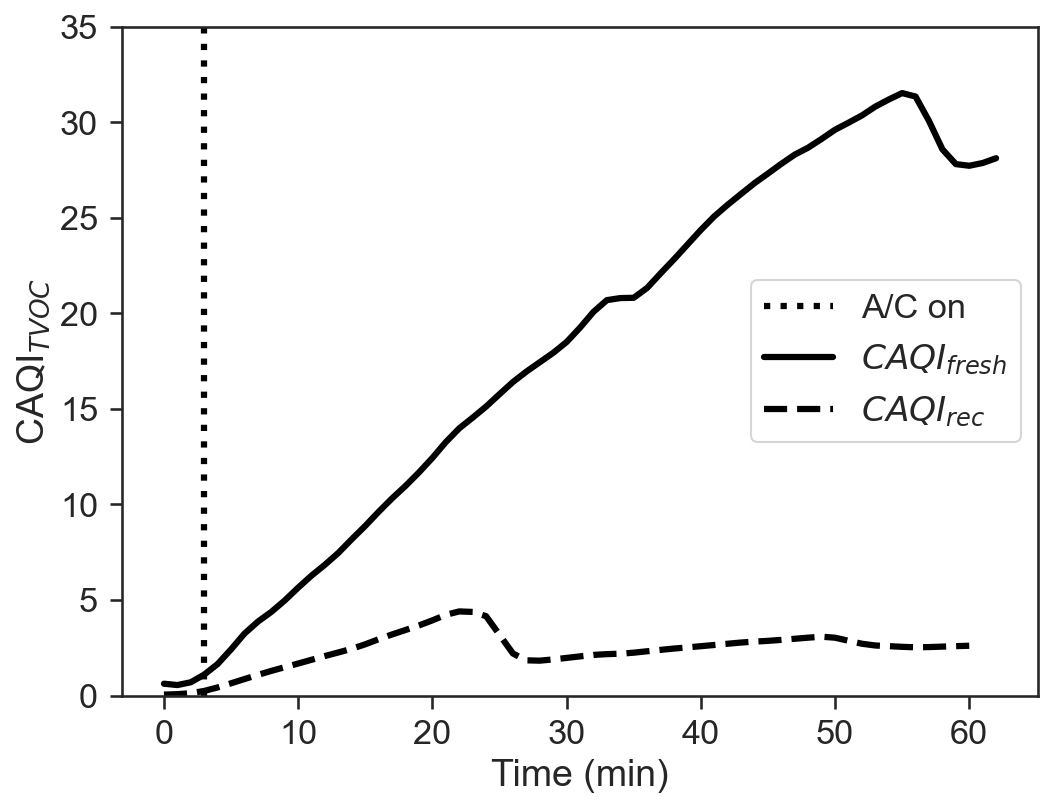

Supplement: Supplementary file 1 [file sensors-22-00543-s001.zip › sensors-1450505 -supp -for pub-final/img/caqi_voc.png]

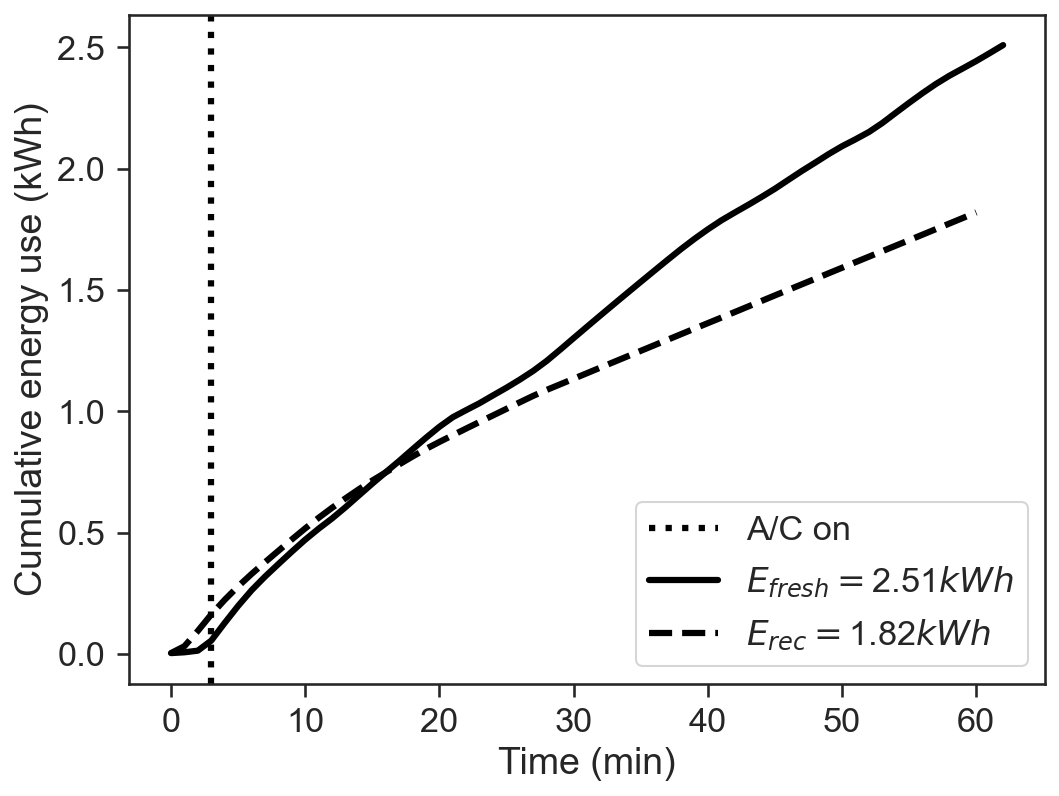

Supplement: Supplementary file 1 [file sensors-22-00543-s001.zip › sensors-1450505 -supp -for pub-final/img/E_ONOFFpng.png]

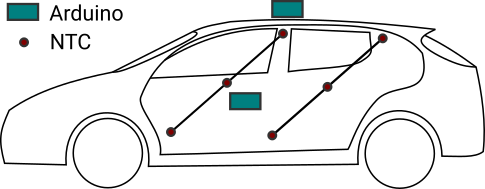

Supplement: Supplementary file 1 [file sensors-22-00543-s001.zip › sensors-1450505 -supp -for pub-final/img/leaf_scheme.png]

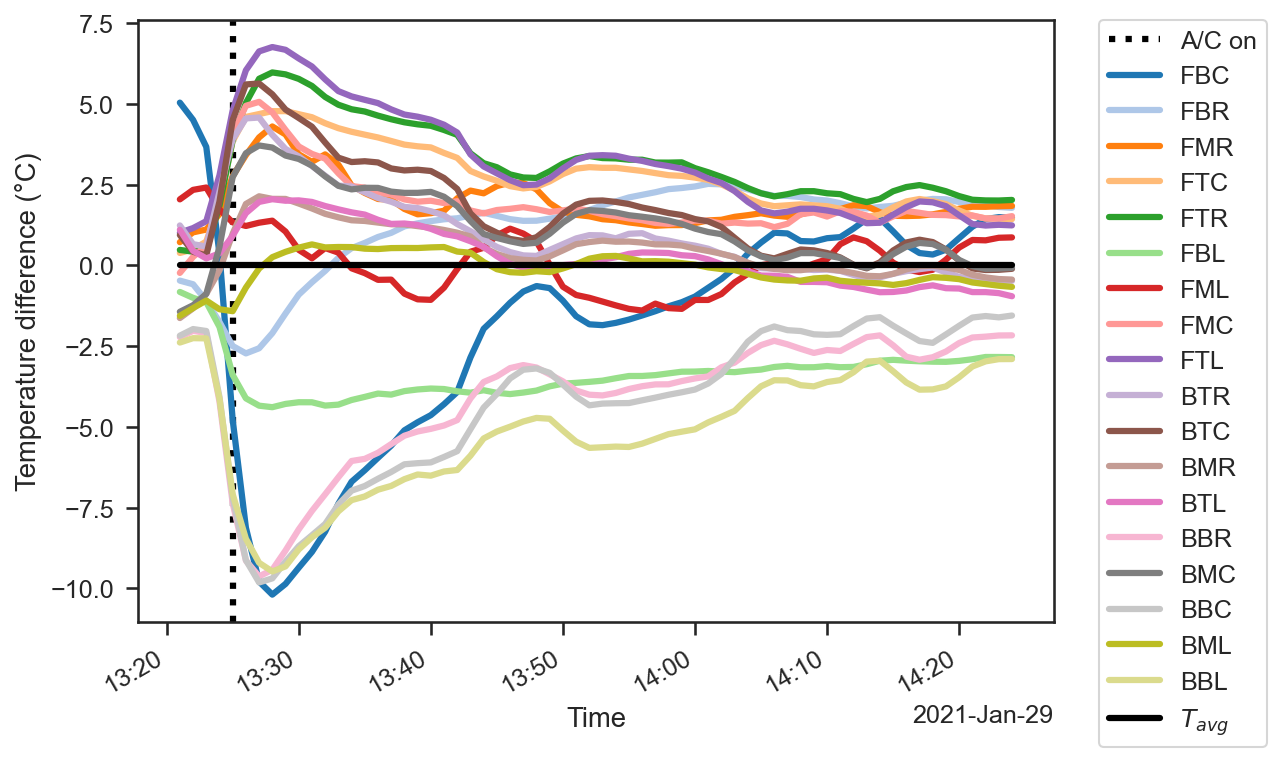

Supplement: Supplementary file 1 [file sensors-22-00543-s001.zip › sensors-1450505 -supp -for pub-final/img/multi_dT_recOFF.png]

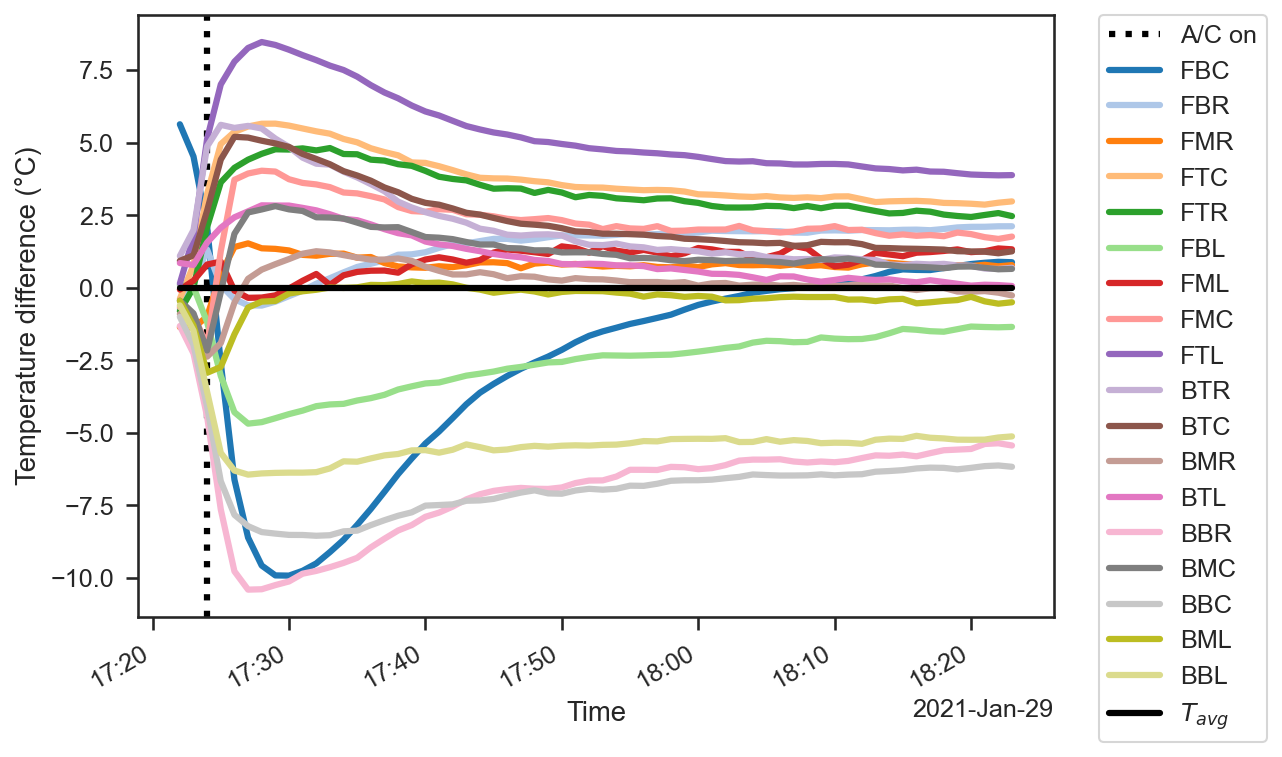

Supplement: Supplementary file 1 [file sensors-22-00543-s001.zip › sensors-1450505 -supp -for pub-final/img/multi_dT_recON.png]

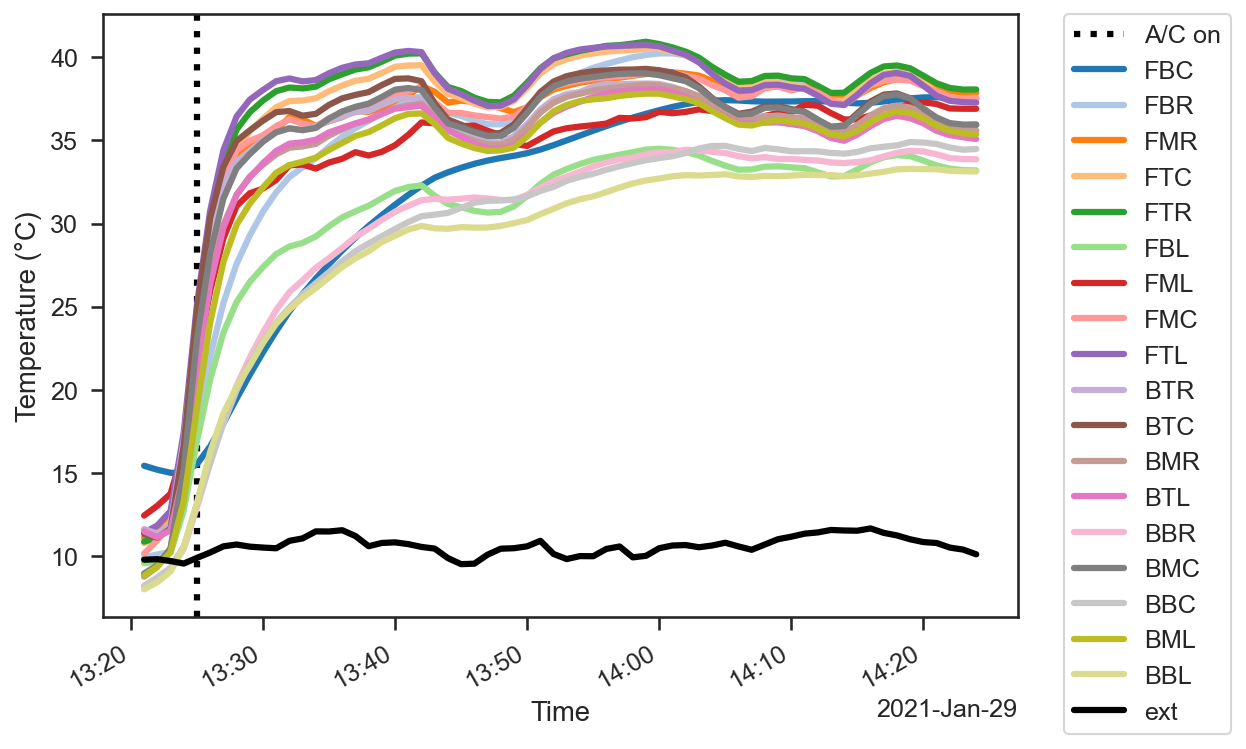

Supplement: Supplementary file 1 [file sensors-22-00543-s001.zip › sensors-1450505 -supp -for pub-final/img/multi_T_recOFF.png]

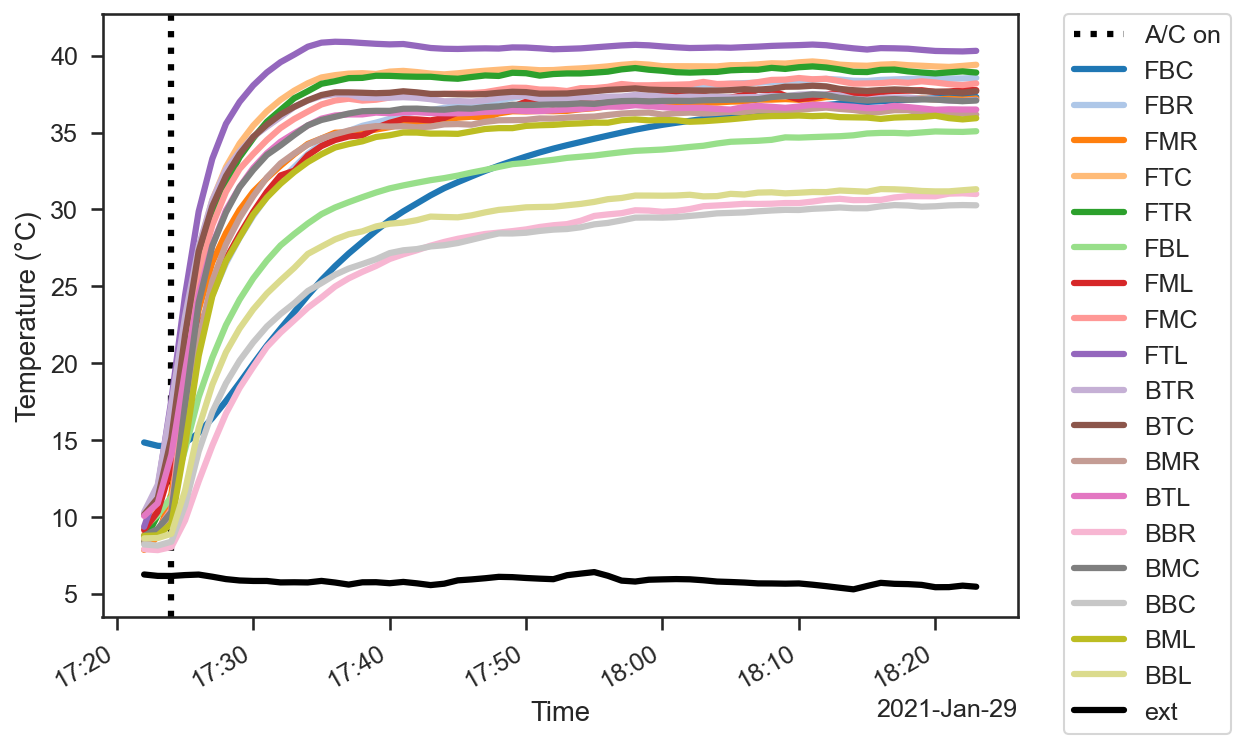

Supplement: Supplementary file 1 [file sensors-22-00543-s001.zip › sensors-1450505 -supp -for pub-final/img/multi_T_recON.png]

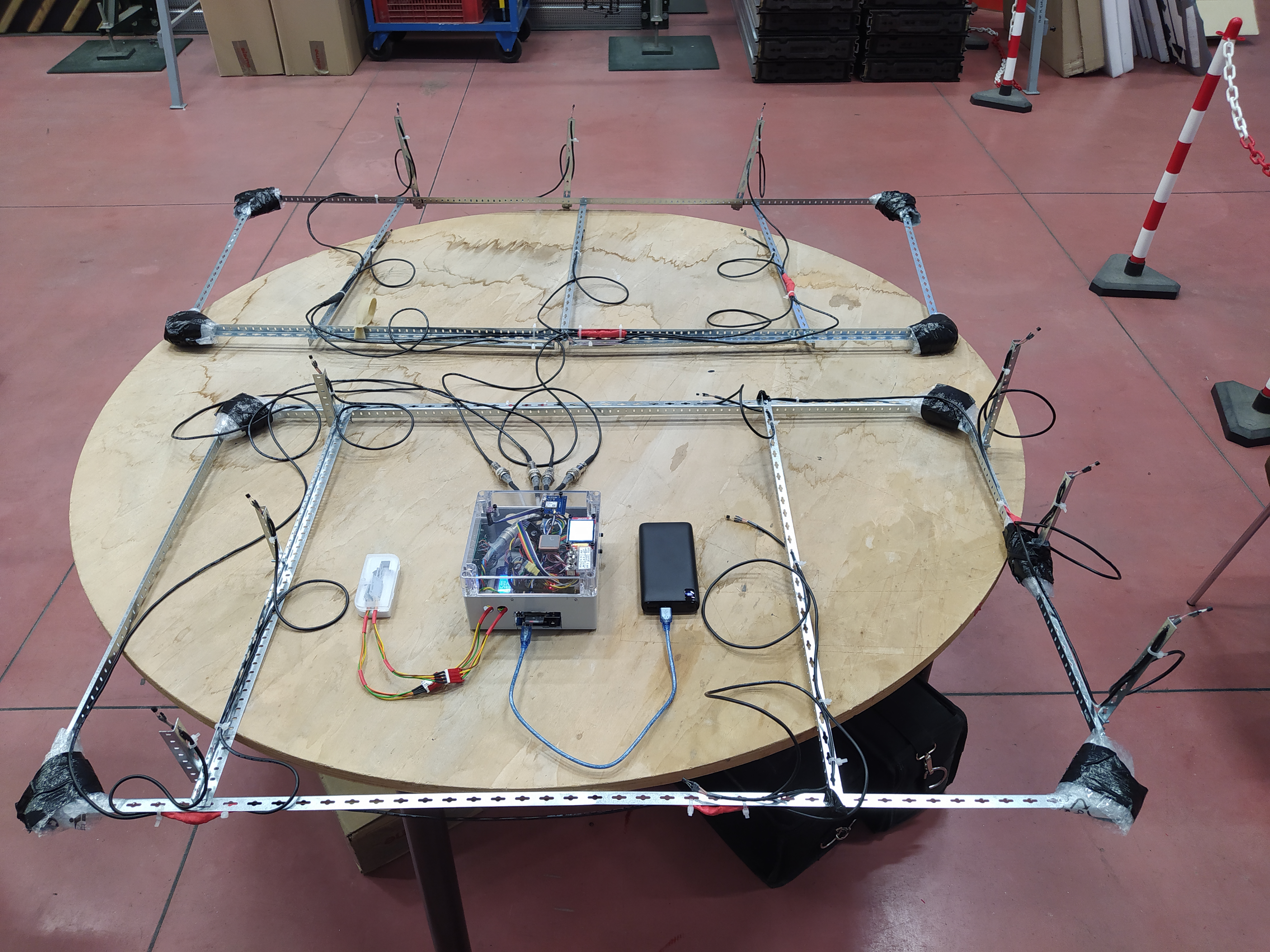

Supplement: Supplementary file 1 [file sensors-22-00543-s001.zip › sensors-1450505 -supp -for pub-final/img/nose_int_grids .jpg]

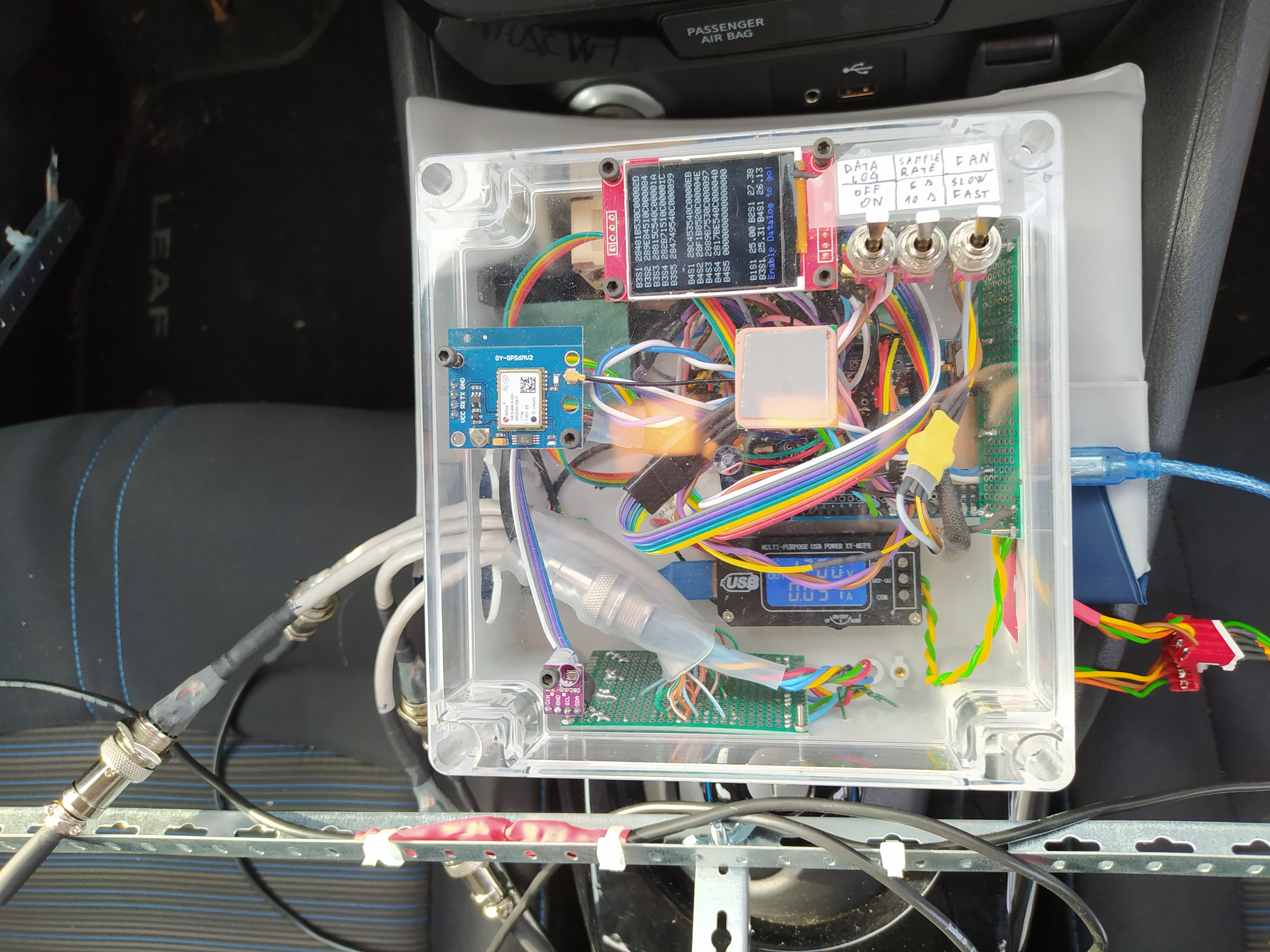

Supplement: Supplementary file 1 [file sensors-22-00543-s001.zip › sensors-1450505 -supp -for pub-final/img/nose_int_only.jpg]

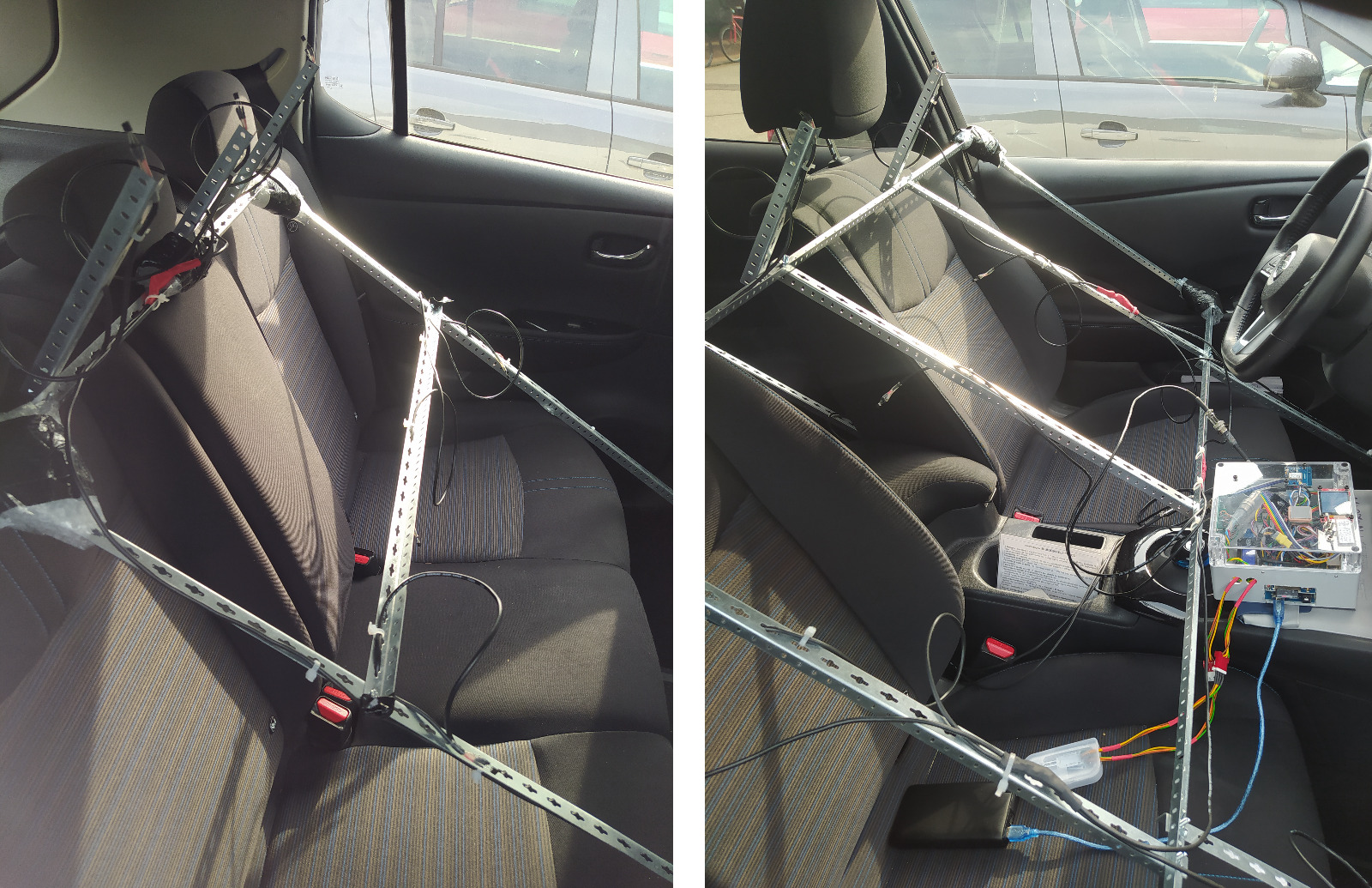

Supplement: Supplementary file 1 [file sensors-22-00543-s001.zip › sensors-1450505 -supp -for pub-final/img/nose_int_placed.jpeg]

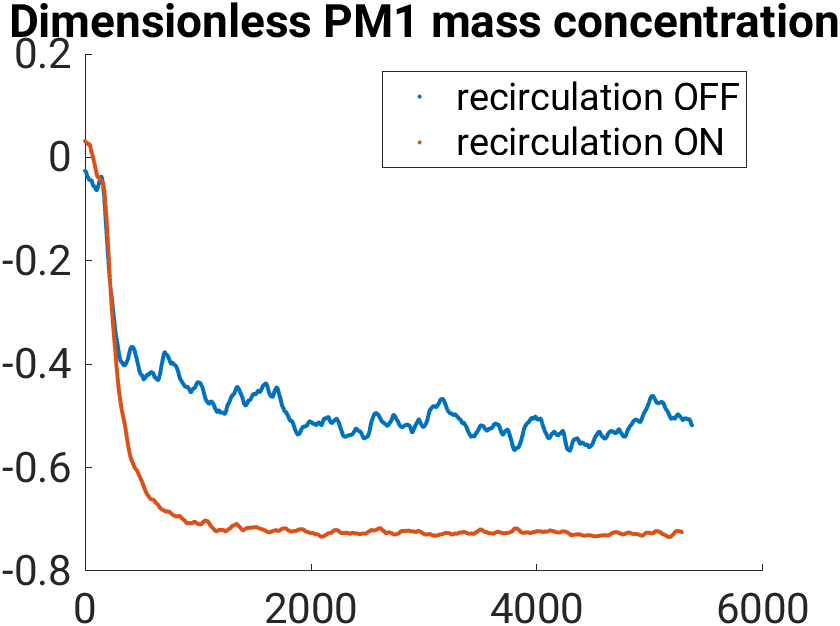

Supplement: Supplementary file 1 [file sensors-22-00543-s001.zip › sensors-1450505 -supp -for pub-final/img/PM1_adim.png]

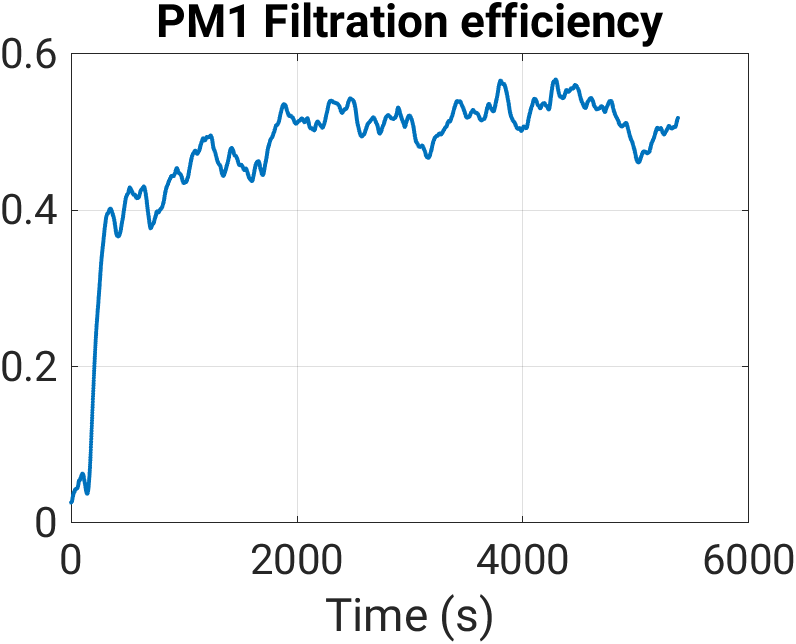

Supplement: Supplementary file 1 [file sensors-22-00543-s001.zip › sensors-1450505 -supp -for pub-final/img/PM1_eta_recOFF.png]

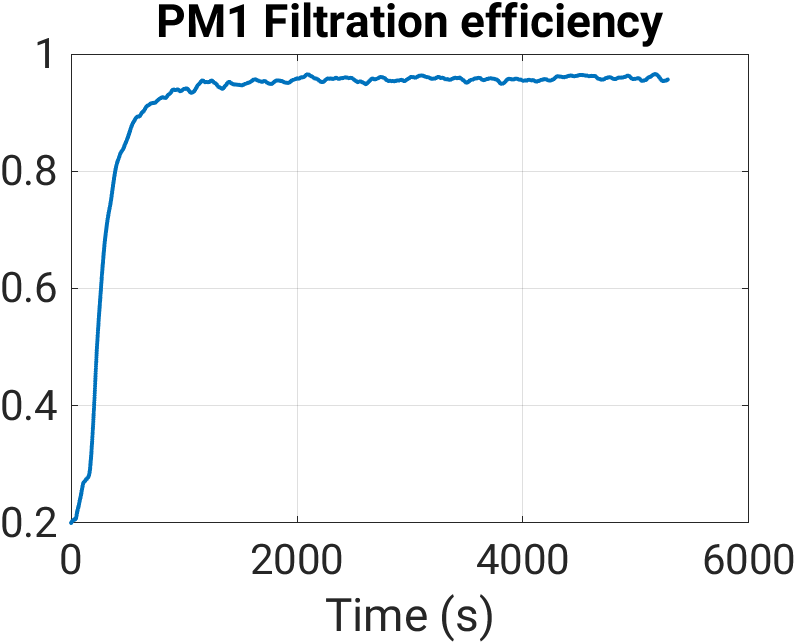

Supplement: Supplementary file 1 [file sensors-22-00543-s001.zip › sensors-1450505 -supp -for pub-final/img/PM1_eta_recON.png]

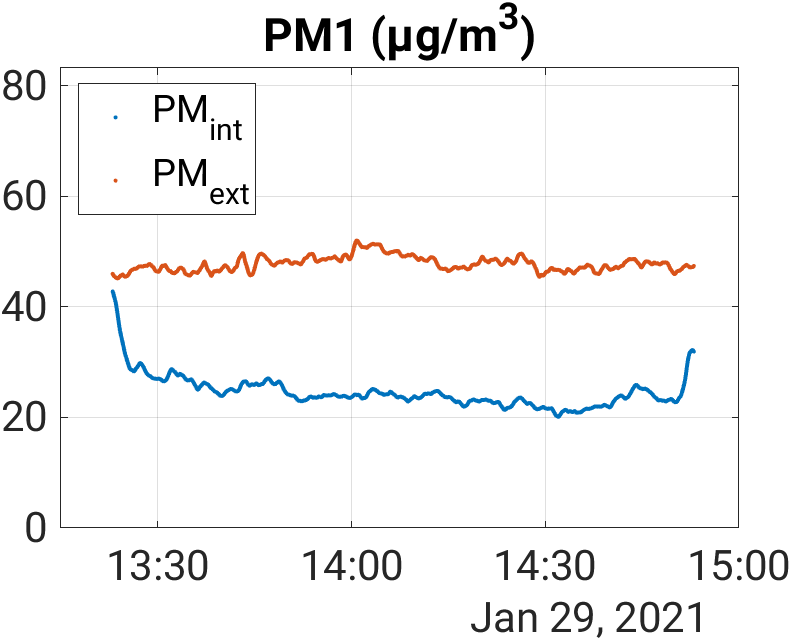

Supplement: Supplementary file 1 [file sensors-22-00543-s001.zip › sensors-1450505 -supp -for pub-final/img/PM1_recOFF.png]

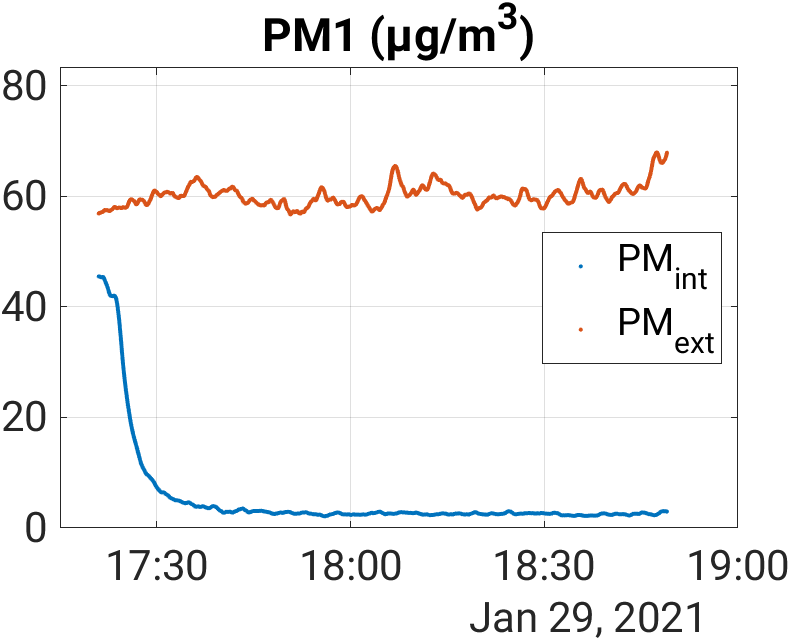

Supplement: Supplementary file 1 [file sensors-22-00543-s001.zip › sensors-1450505 -supp -for pub-final/img/PM1_recON.png]

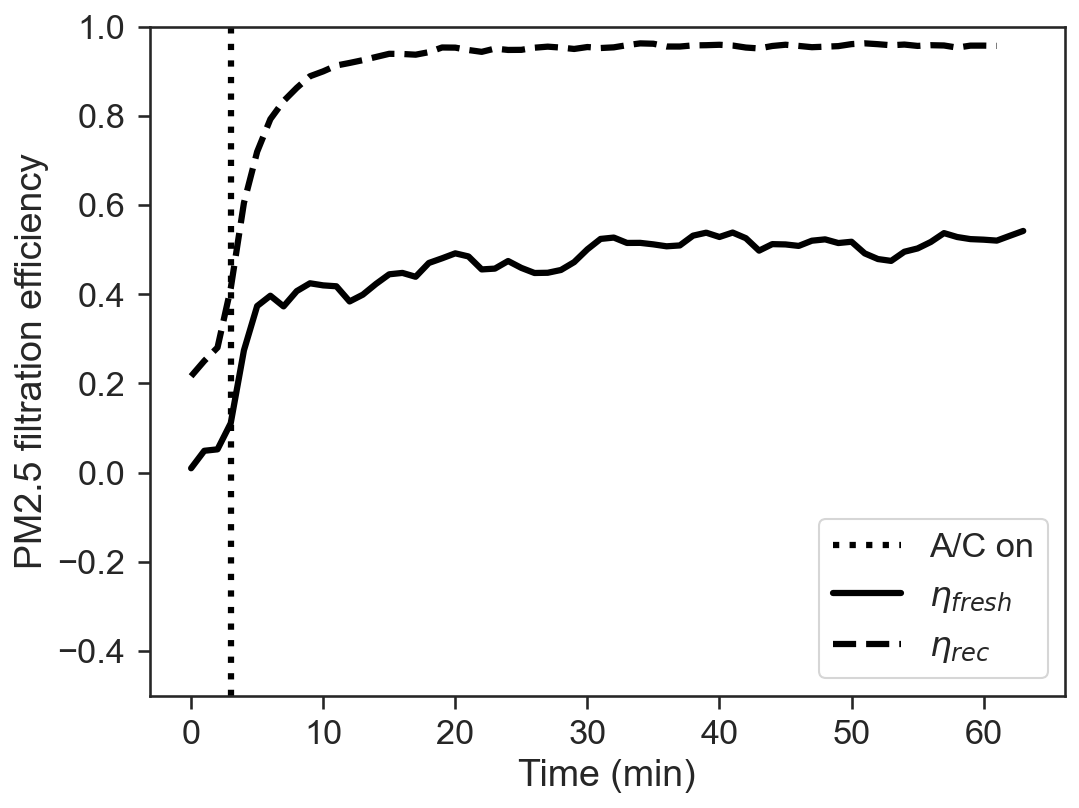

Supplement: Supplementary file 1 [file sensors-22-00543-s001.zip › sensors-1450505 -supp -for pub-final/img/PM25_eta_ONOFF.png]

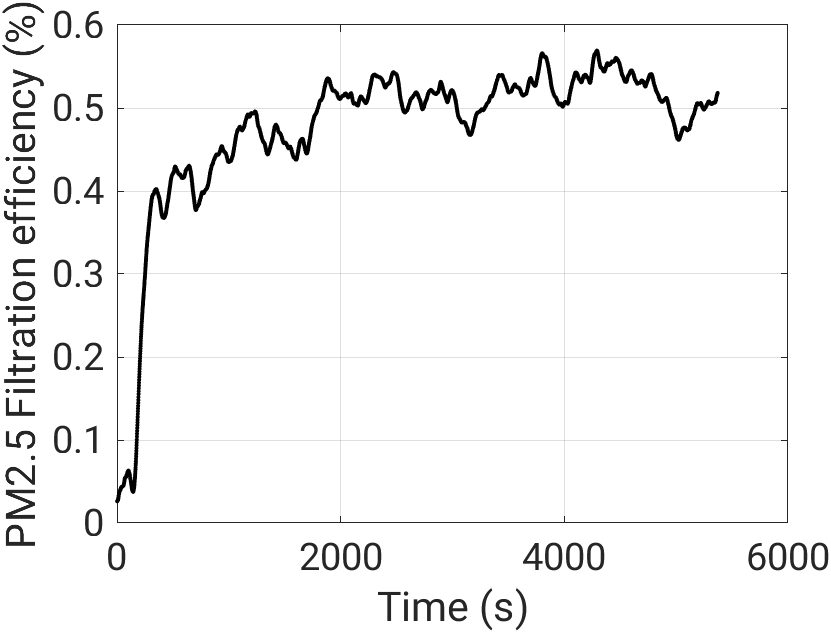

Supplement: Supplementary file 1 [file sensors-22-00543-s001.zip › sensors-1450505 -supp -for pub-final/img/PM25_eta_recOFF.png]

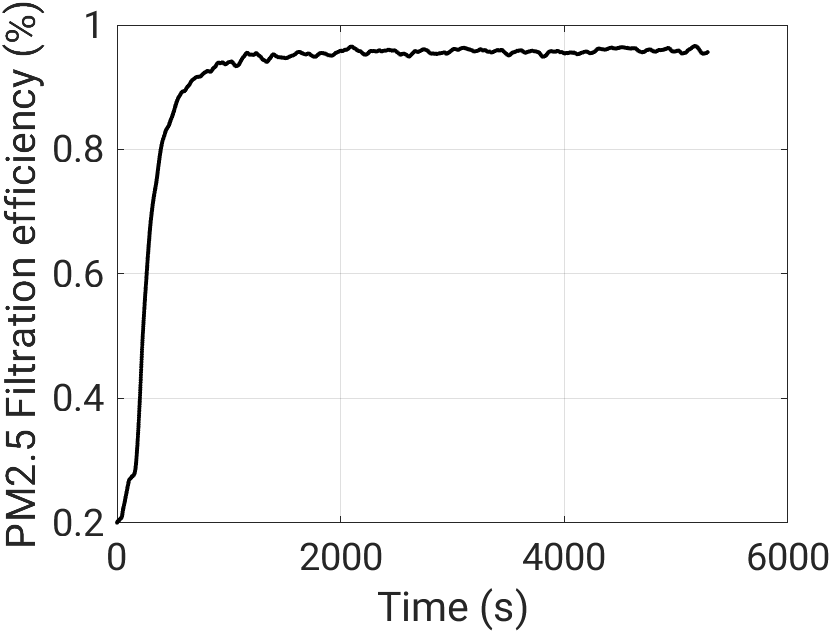

Supplement: Supplementary file 1 [file sensors-22-00543-s001.zip › sensors-1450505 -supp -for pub-final/img/PM25_eta_recON.png]

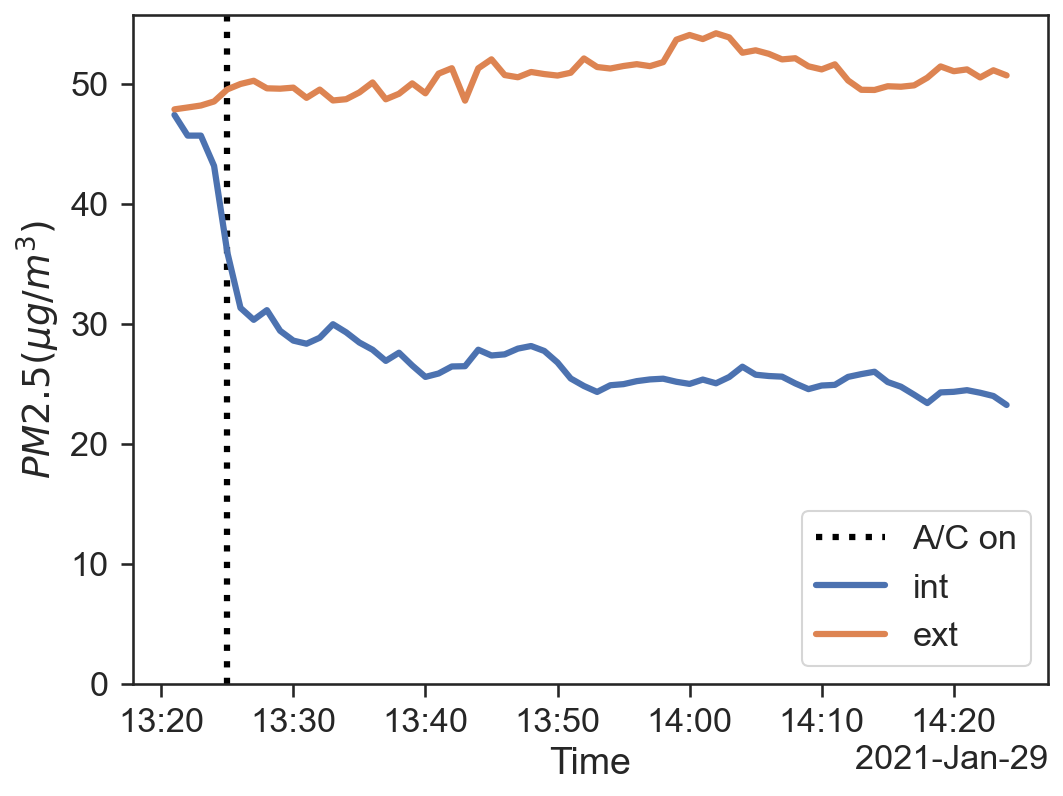

Supplement: Supplementary file 1 [file sensors-22-00543-s001.zip › sensors-1450505 -supp -for pub-final/img/PM25_recOFF.png]

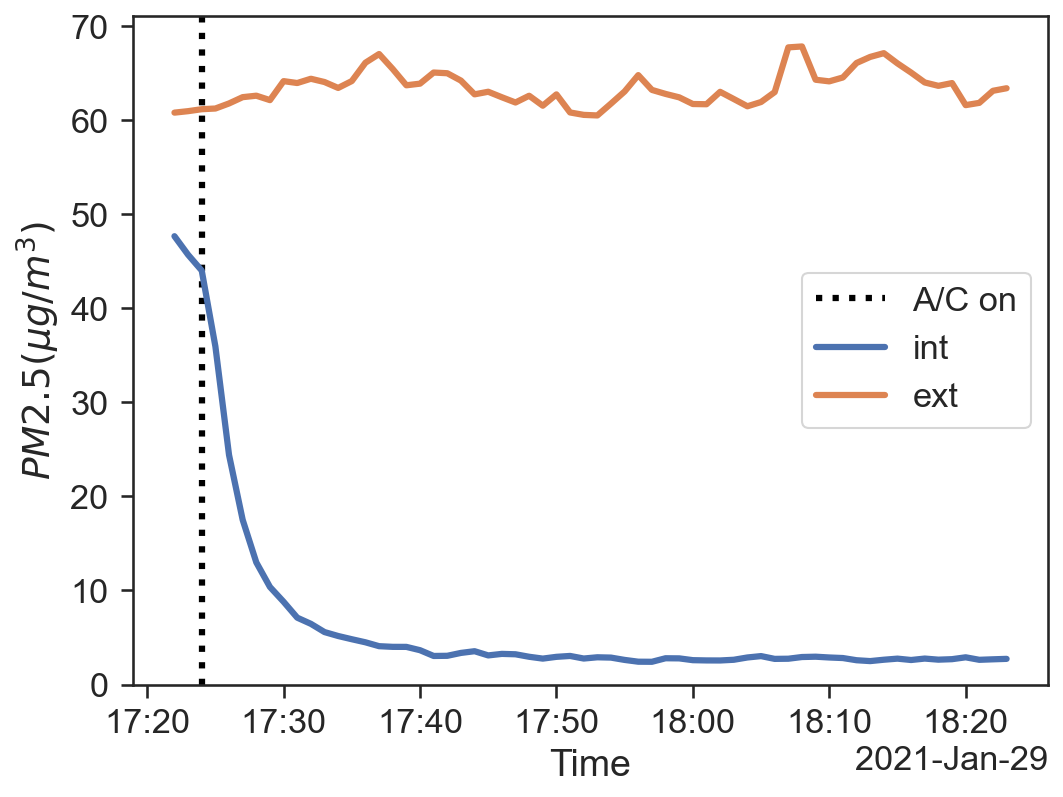

Supplement: Supplementary file 1 [file sensors-22-00543-s001.zip › sensors-1450505 -supp -for pub-final/img/PM25_recON.png]

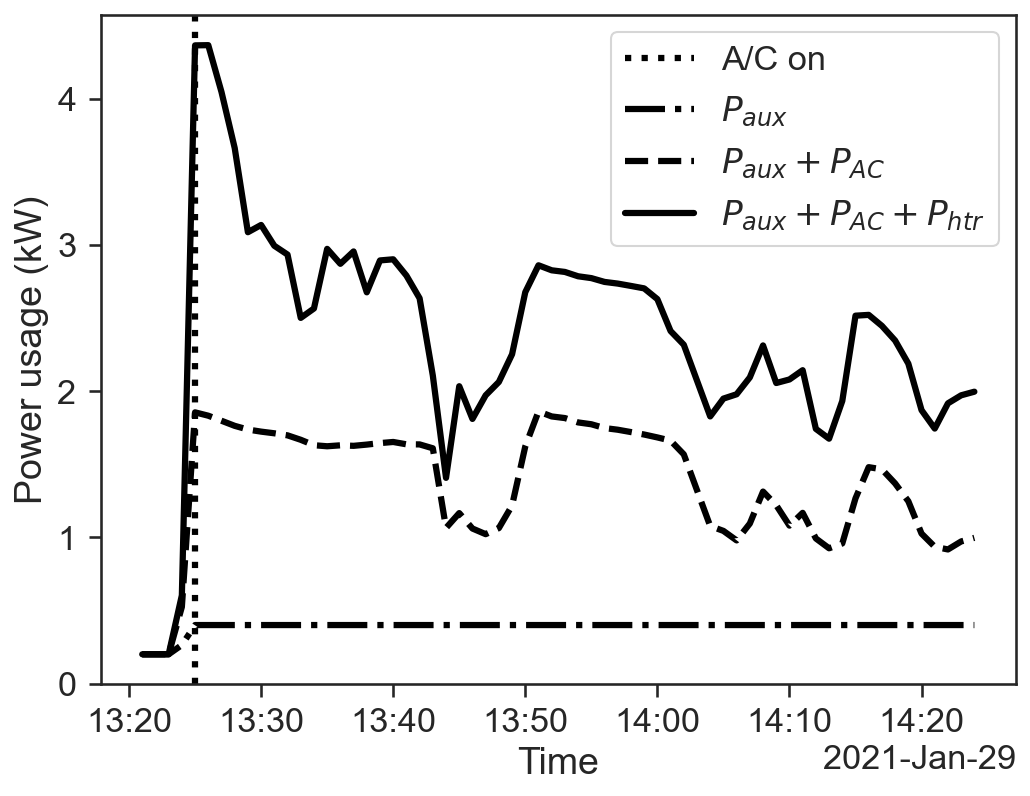

Supplement: Supplementary file 1 [file sensors-22-00543-s001.zip › sensors-1450505 -supp -for pub-final/img/P_recOFF.png]

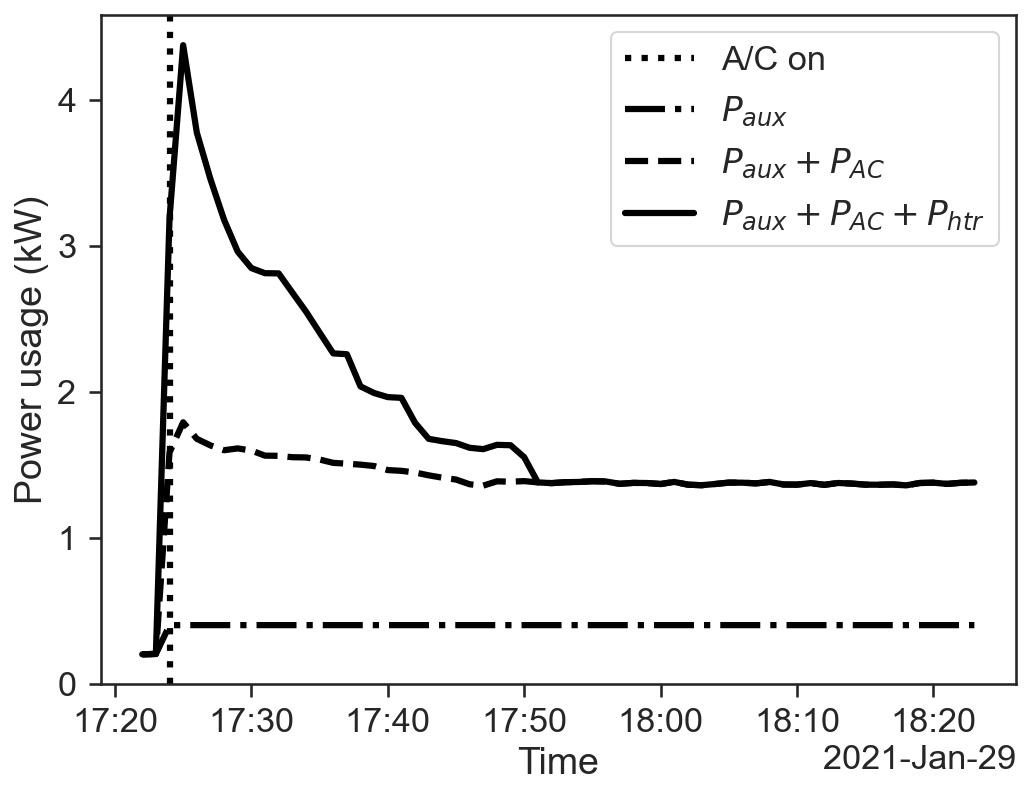

Supplement: Supplementary file 1 [file sensors-22-00543-s001.zip › sensors-1450505 -supp -for pub-final/img/P_recON.png]

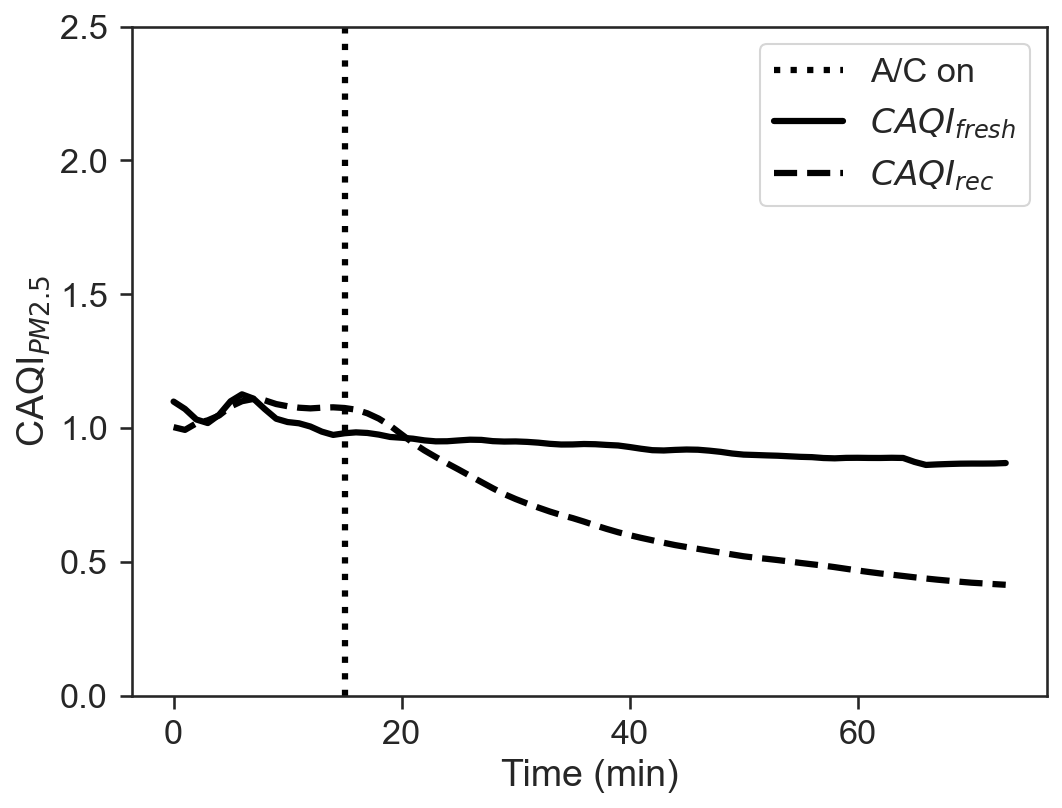

Supplement: Supplementary file 1 [file sensors-22-00543-s001.zip › sensors-1450505 -supp -for pub-final/img/summer/caqi.png]

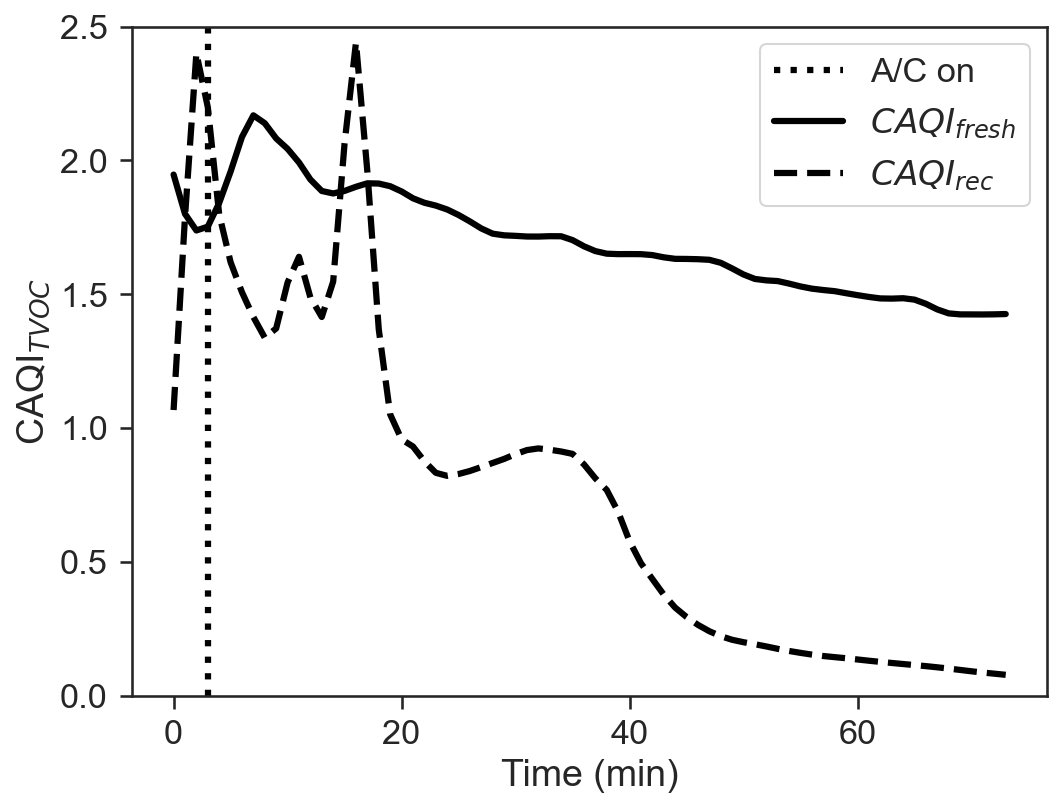

Supplement: Supplementary file 1 [file sensors-22-00543-s001.zip › sensors-1450505 -supp -for pub-final/img/summer/caqi_voc.png]

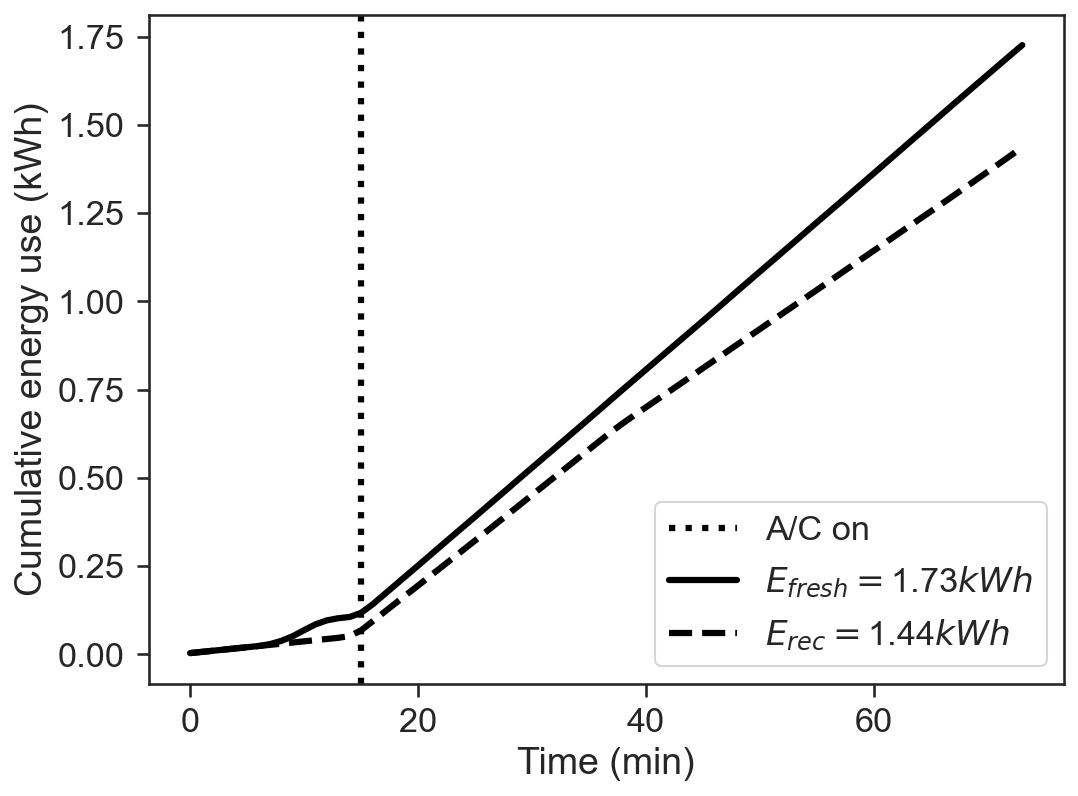

Supplement: Supplementary file 1 [file sensors-22-00543-s001.zip › sensors-1450505 -supp -for pub-final/img/summer/E_ONOFFpng.png]

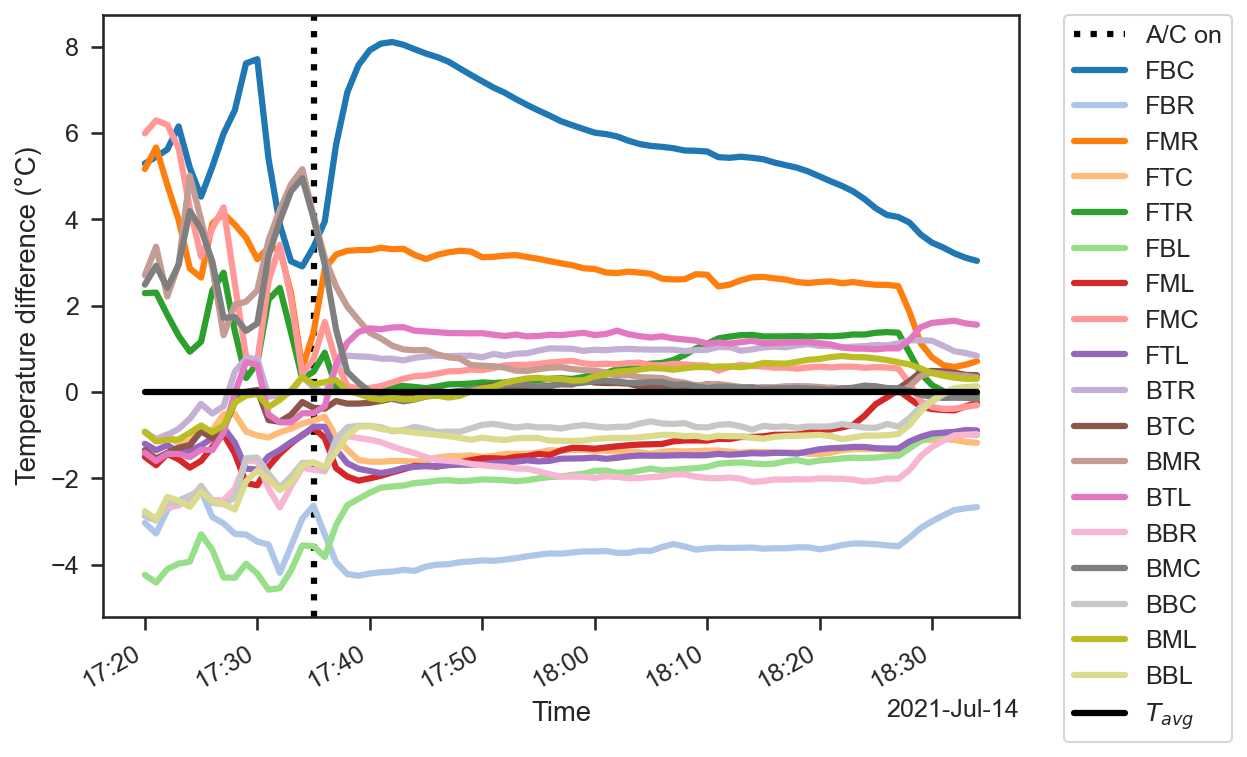

Supplement: Supplementary file 1 [file sensors-22-00543-s001.zip › sensors-1450505 -supp -for pub-final/img/summer/multi_dT_recOFF.png]

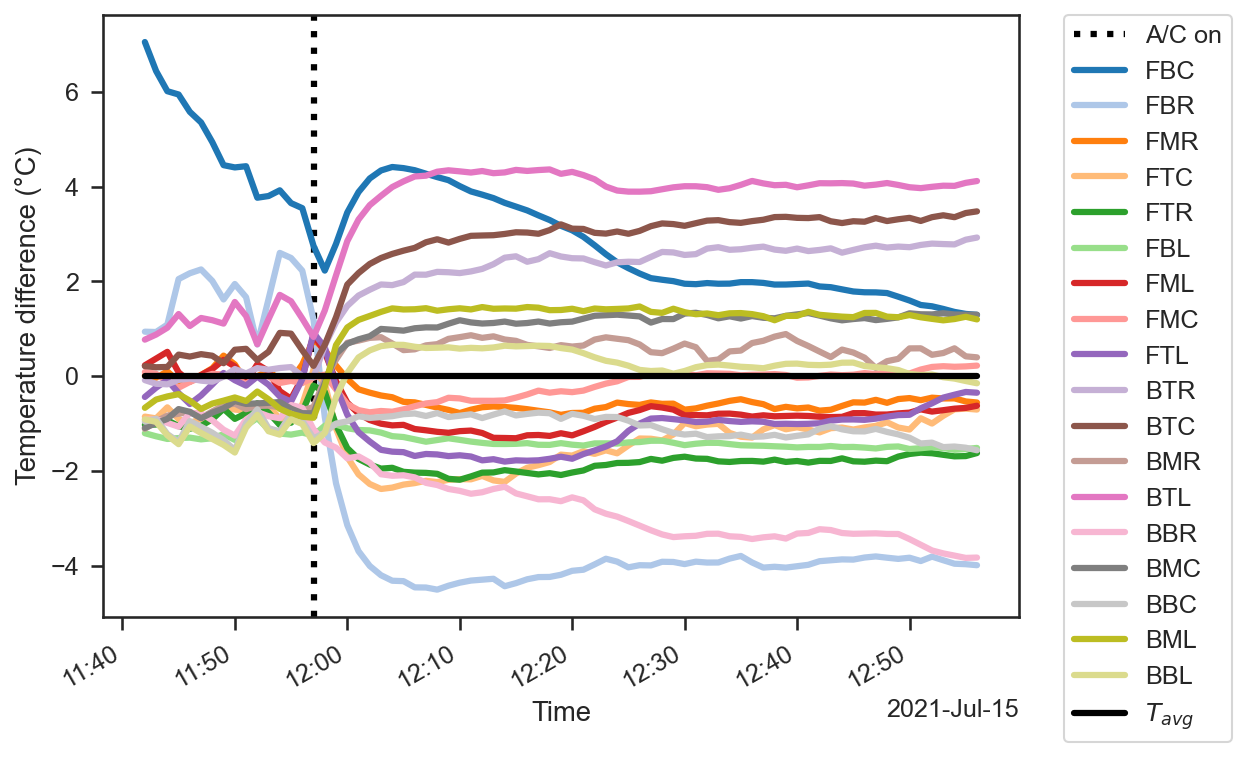

Supplement: Supplementary file 1 [file sensors-22-00543-s001.zip › sensors-1450505 -supp -for pub-final/img/summer/multi_dT_recON.png]

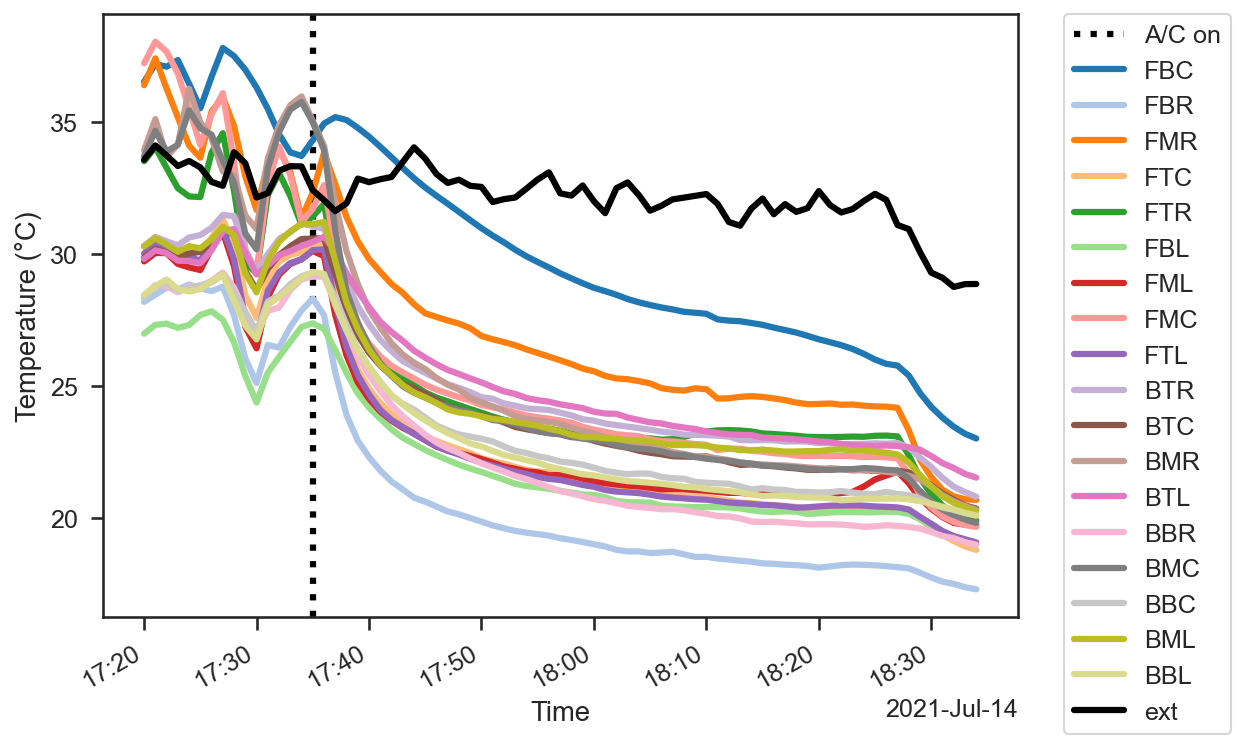

Supplement: Supplementary file 1 [file sensors-22-00543-s001.zip › sensors-1450505 -supp -for pub-final/img/summer/multi_T_recOFF.png]

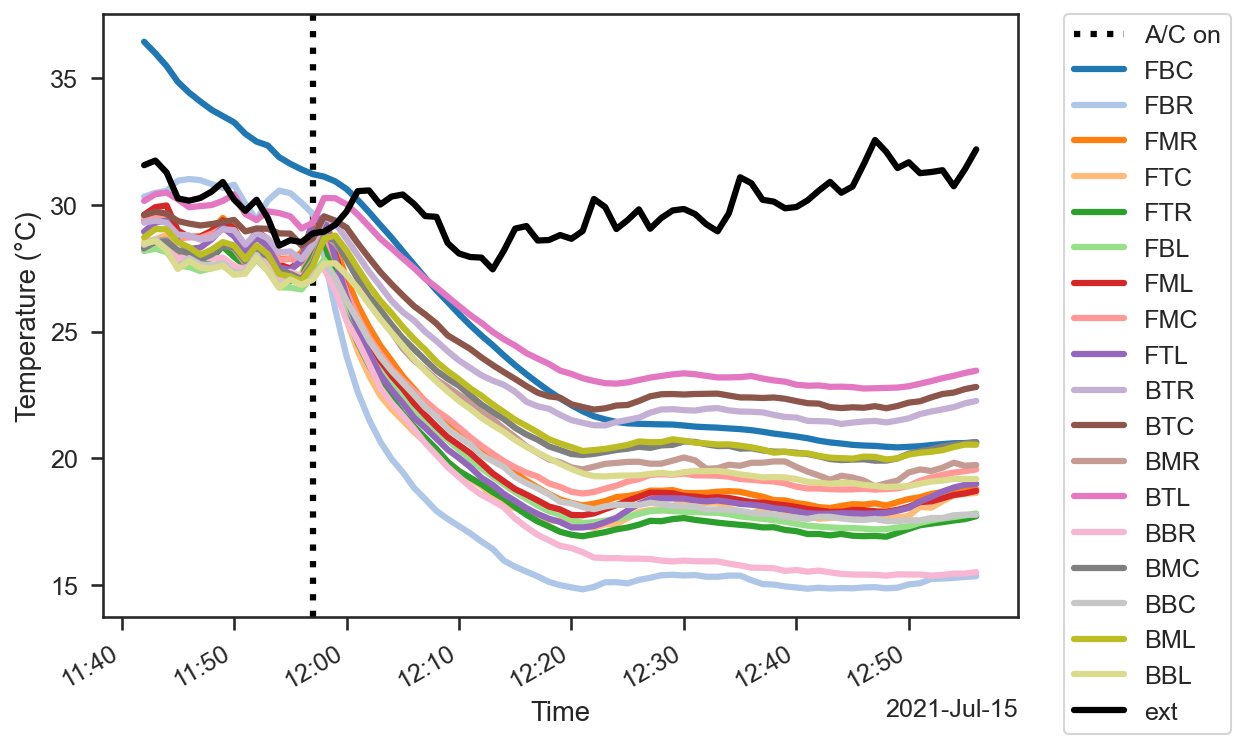

Supplement: Supplementary file 1 [file sensors-22-00543-s001.zip › sensors-1450505 -supp -for pub-final/img/summer/multi_T_recON.png]

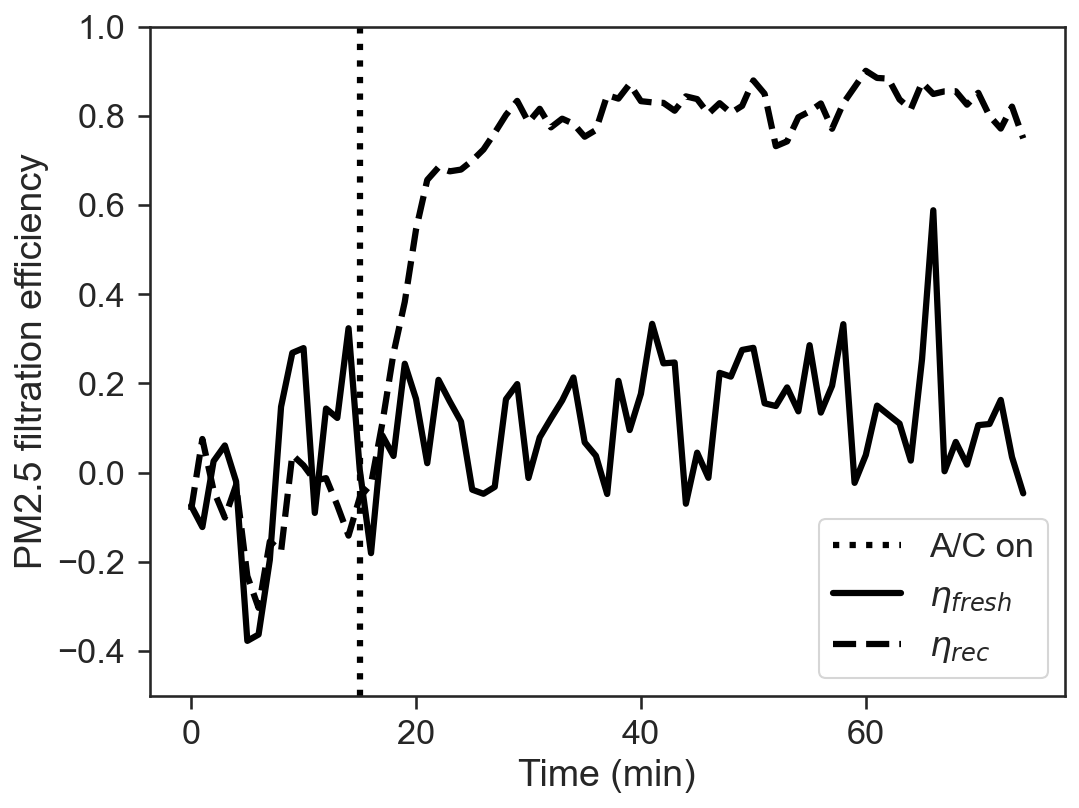

Supplement: Supplementary file 1 [file sensors-22-00543-s001.zip › sensors-1450505 -supp -for pub-final/img/summer/PM25_eta_ONOFF.png]

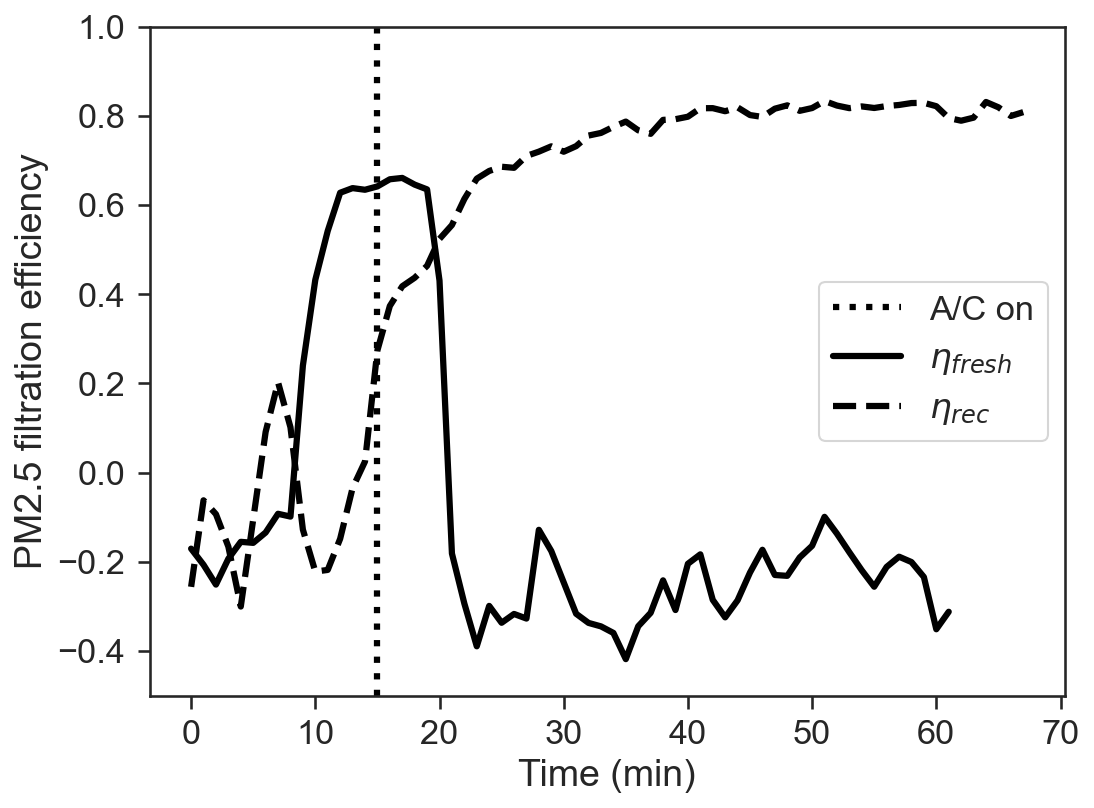

Supplement: Supplementary file 1 [file sensors-22-00543-s001.zip › sensors-1450505 -supp -for pub-final/img/summer/PM25_eta_ONOFF_new.png]

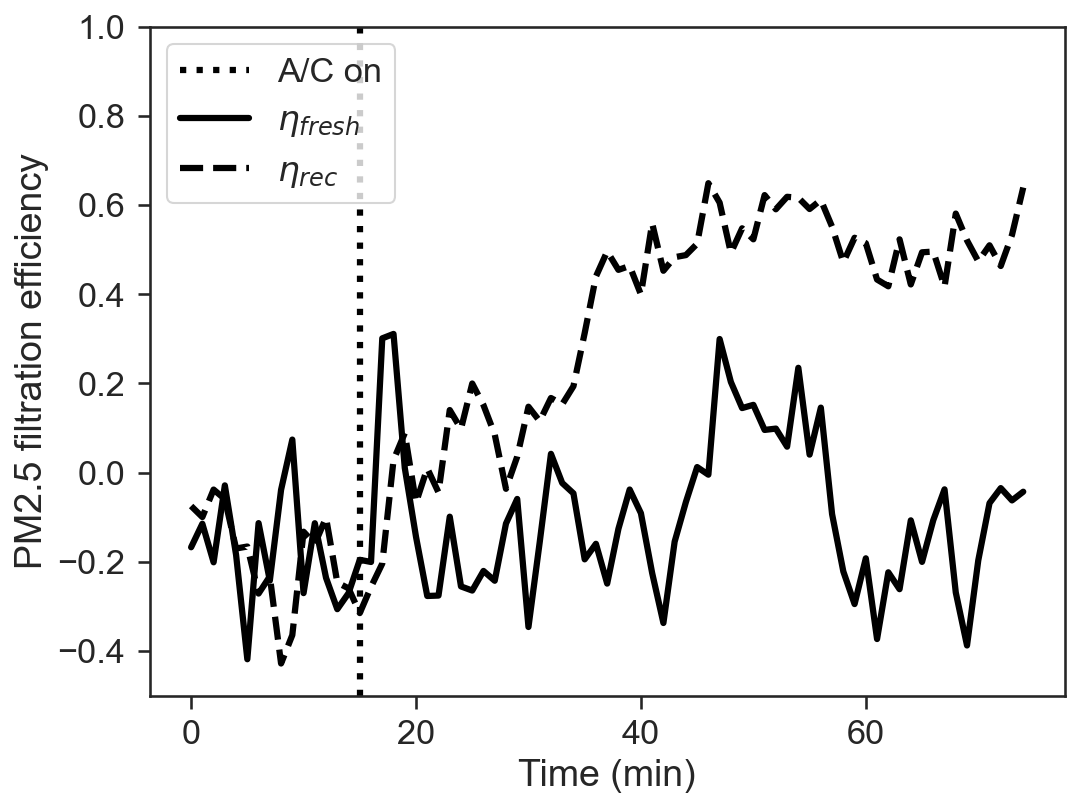

Supplement: Supplementary file 1 [file sensors-22-00543-s001.zip › sensors-1450505 -supp -for pub-final/img/summer/PM25_eta_ONOFF_no.png]

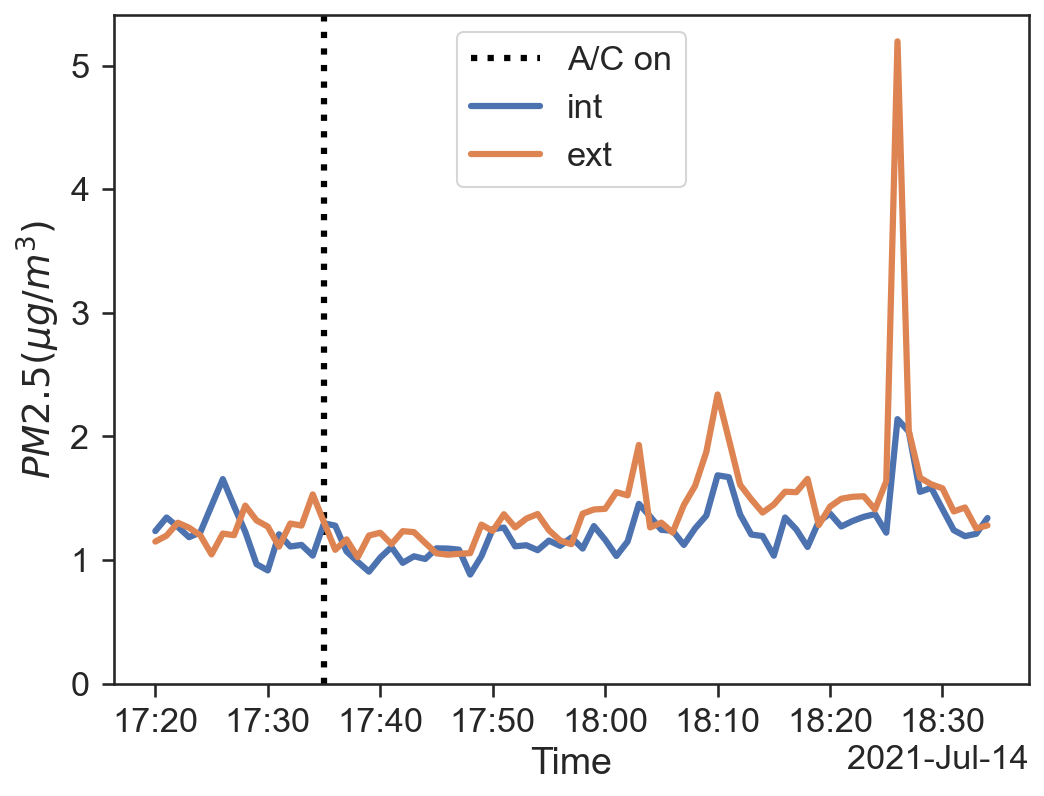

Supplement: Supplementary file 1 [file sensors-22-00543-s001.zip › sensors-1450505 -supp -for pub-final/img/summer/PM25_recOFF.png]

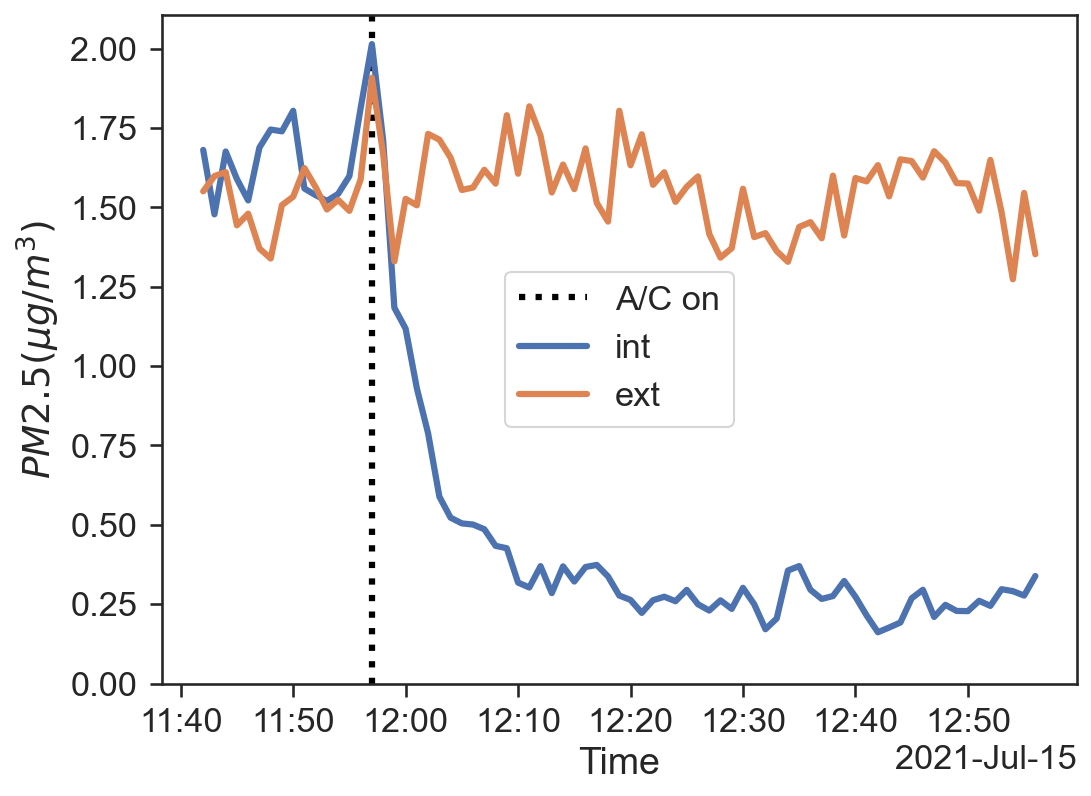

Supplement: Supplementary file 1 [file sensors-22-00543-s001.zip › sensors-1450505 -supp -for pub-final/img/summer/PM25_recON.png]

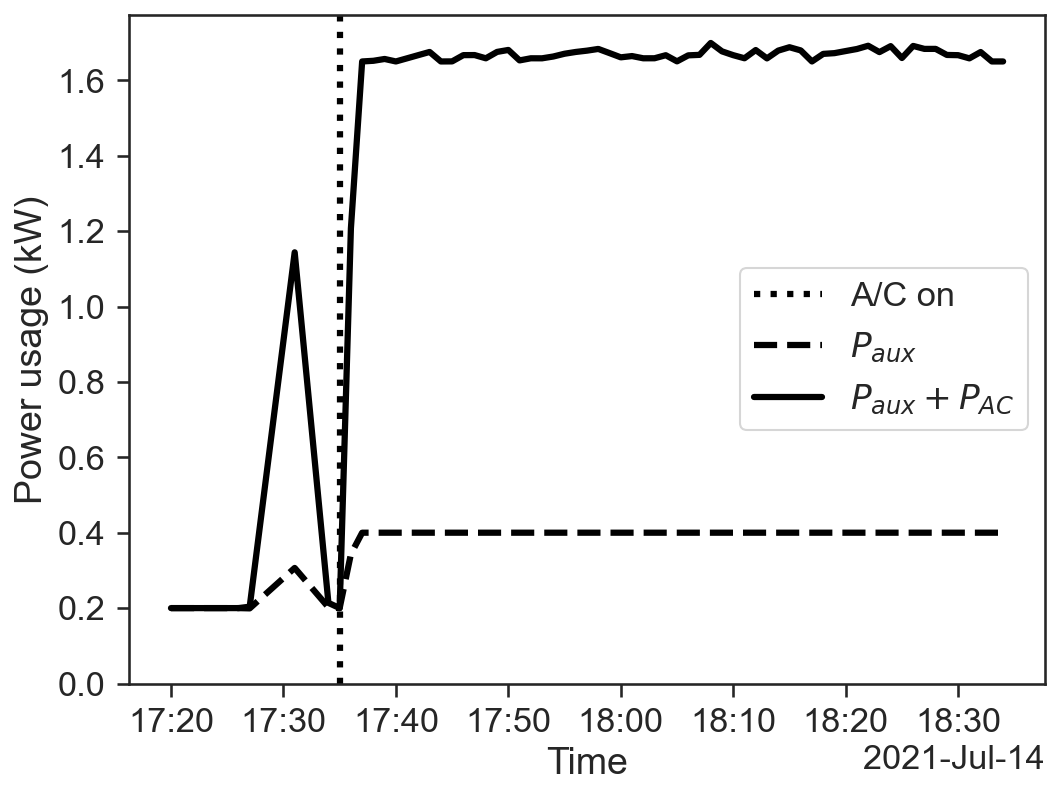

Supplement: Supplementary file 1 [file sensors-22-00543-s001.zip › sensors-1450505 -supp -for pub-final/img/summer/P_recOFF.png]

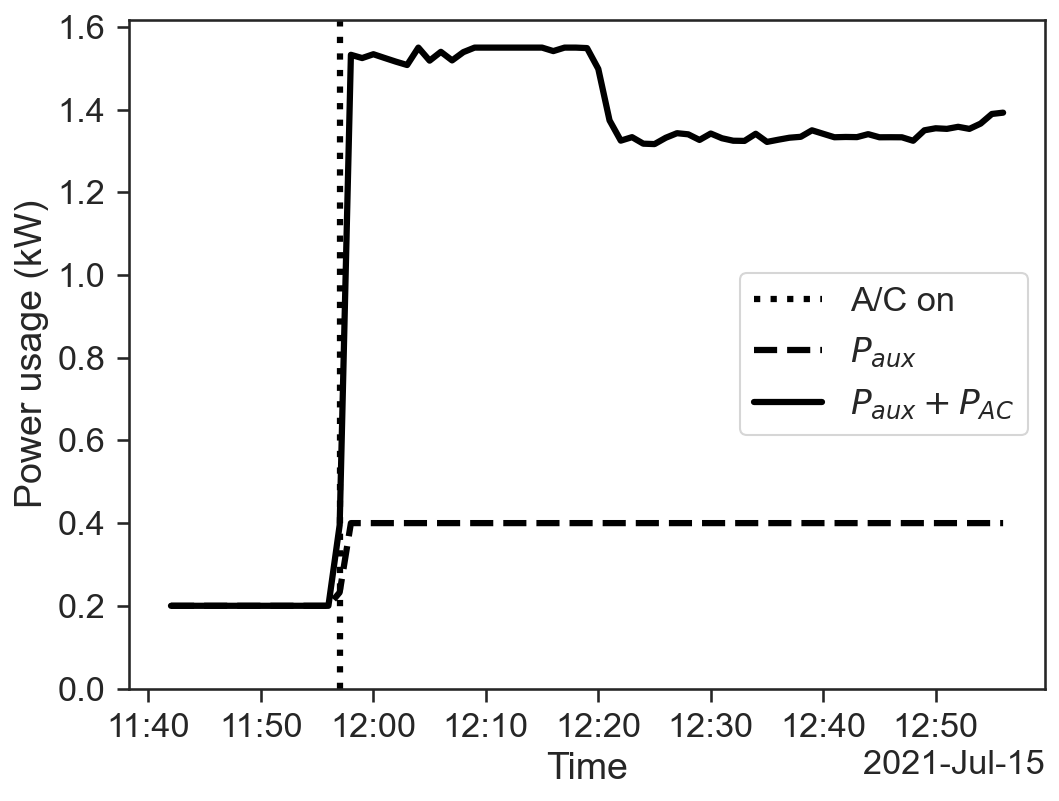

Supplement: Supplementary file 1 [file sensors-22-00543-s001.zip › sensors-1450505 -supp -for pub-final/img/summer/P_recON.png]

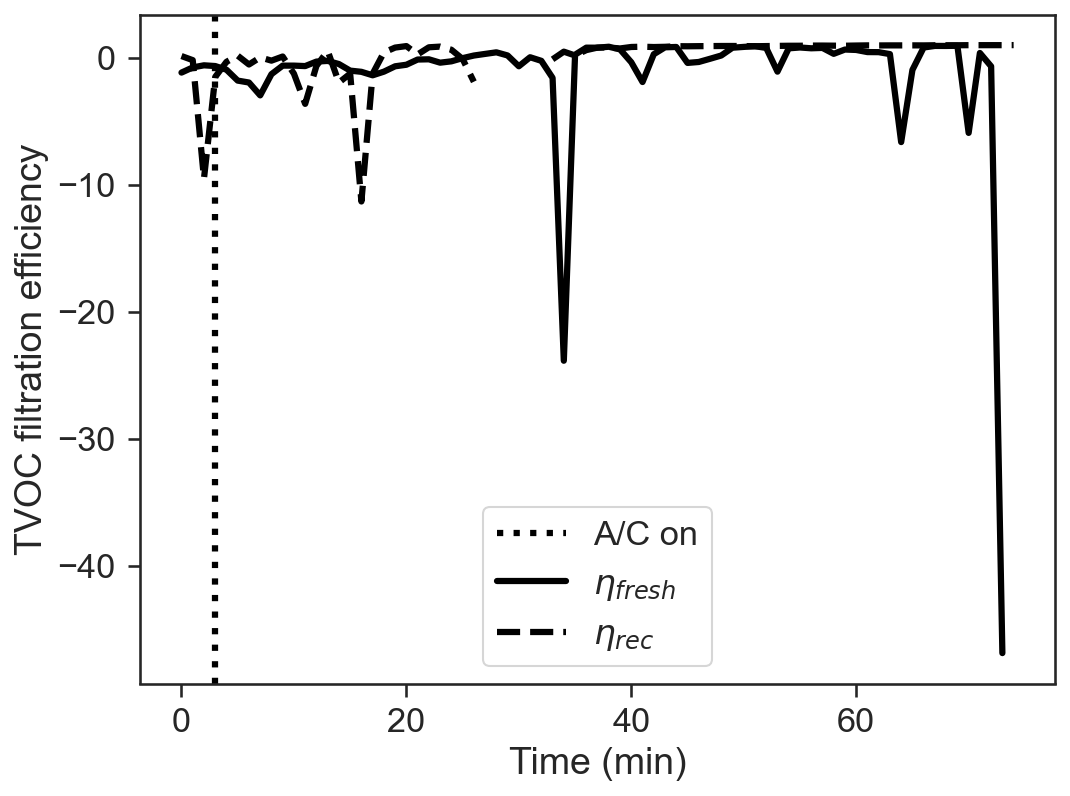

Supplement: Supplementary file 1 [file sensors-22-00543-s001.zip › sensors-1450505 -supp -for pub-final/img/summer/TVOC_eta_ONOFF.png]

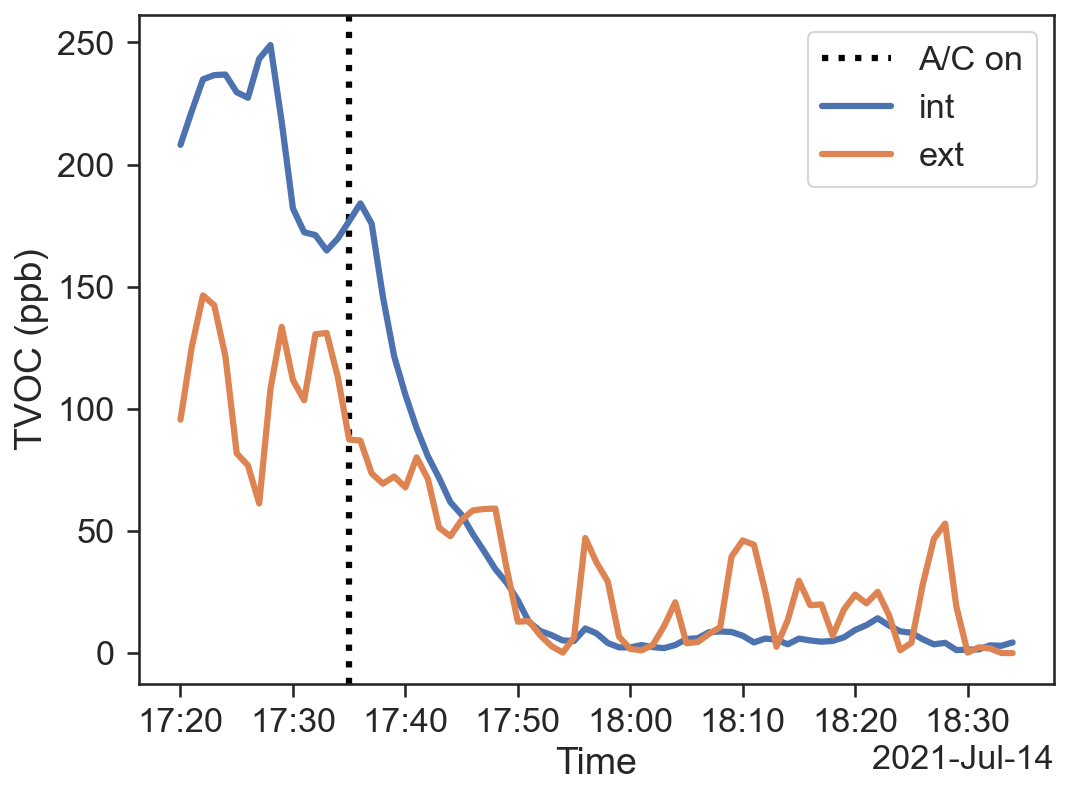

Supplement: Supplementary file 1 [file sensors-22-00543-s001.zip › sensors-1450505 -supp -for pub-final/img/summer/TVOC_recOFF.png]

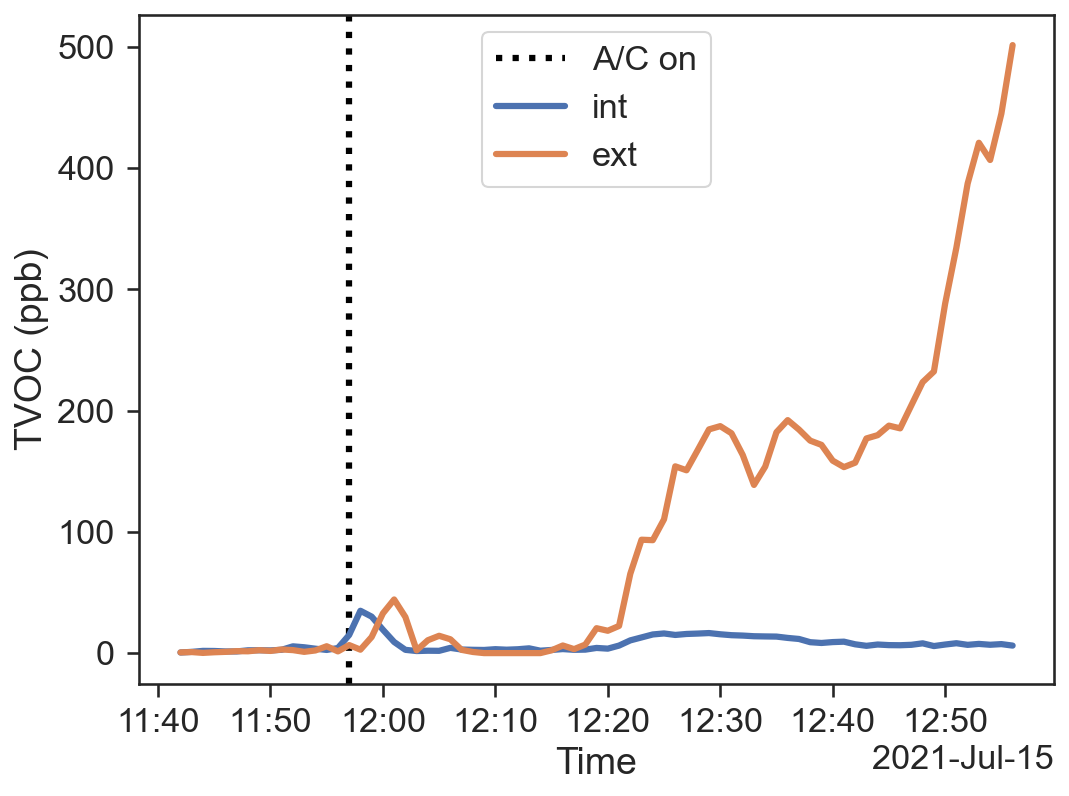

Supplement: Supplementary file 1 [file sensors-22-00543-s001.zip › sensors-1450505 -supp -for pub-final/img/summer/TVOC_recON.png]

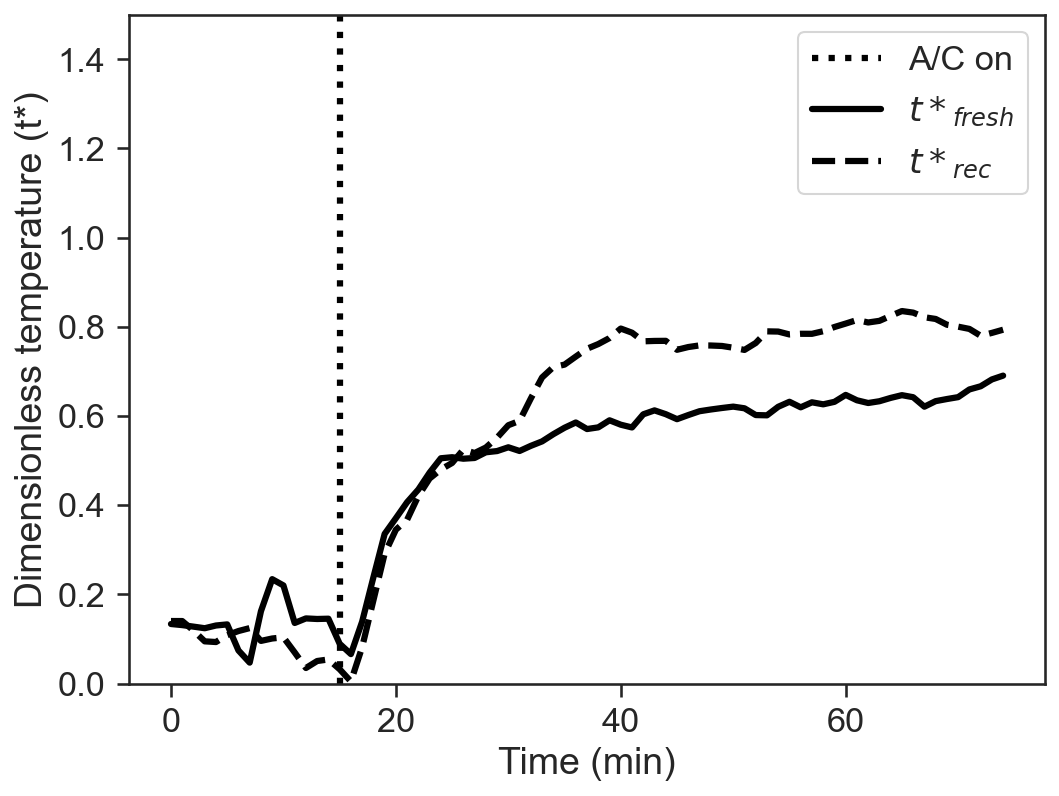

Supplement: Supplementary file 1 [file sensors-22-00543-s001.zip › sensors-1450505 -supp -for pub-final/img/summer/T_adim.png]

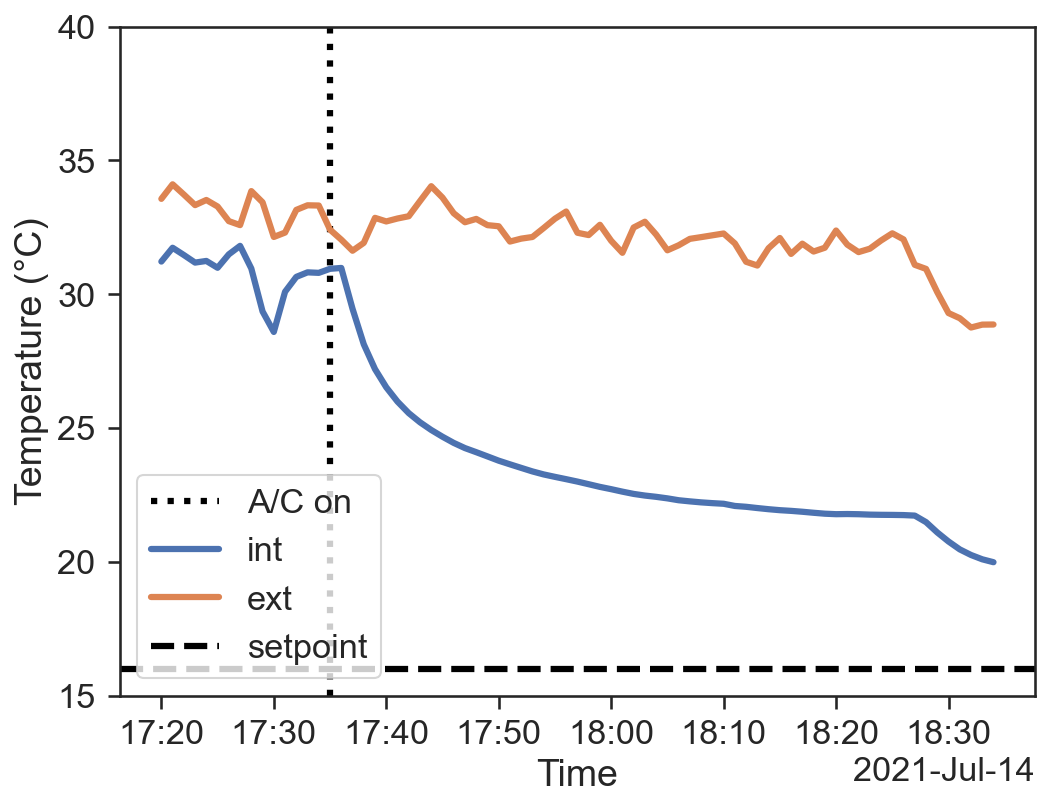

Supplement: Supplementary file 1 [file sensors-22-00543-s001.zip › sensors-1450505 -supp -for pub-final/img/summer/T_recOFF.png]

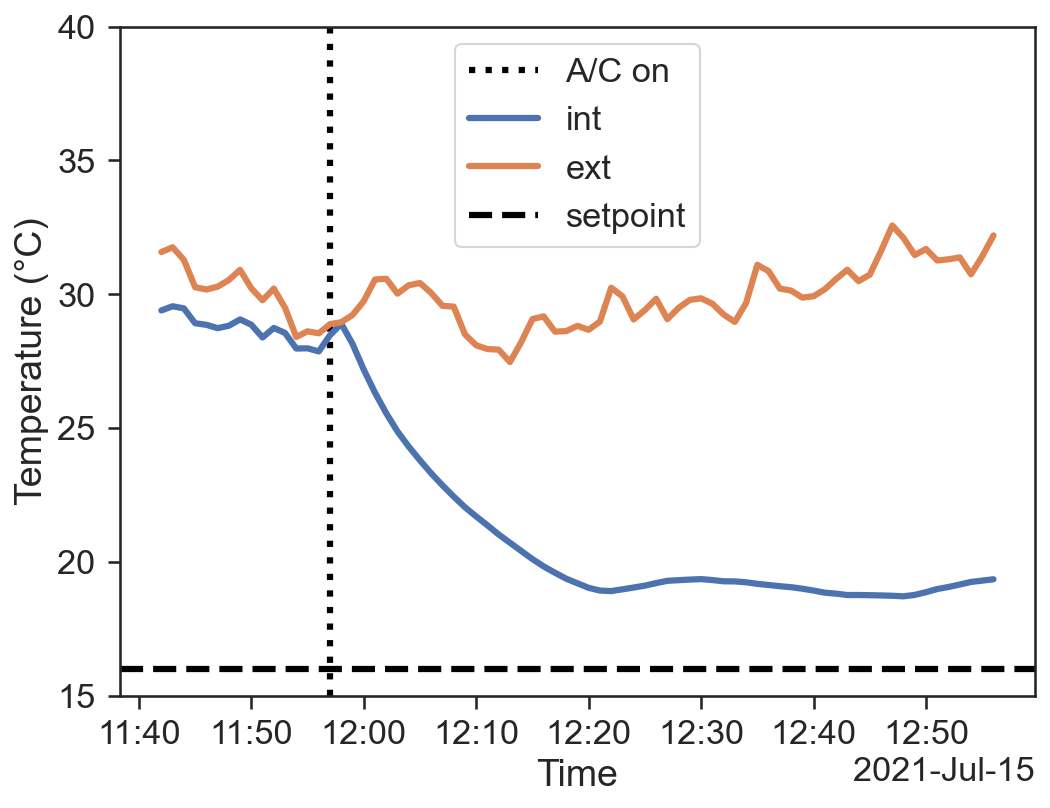

Supplement: Supplementary file 1 [file sensors-22-00543-s001.zip › sensors-1450505 -supp -for pub-final/img/summer/T_recON.png]

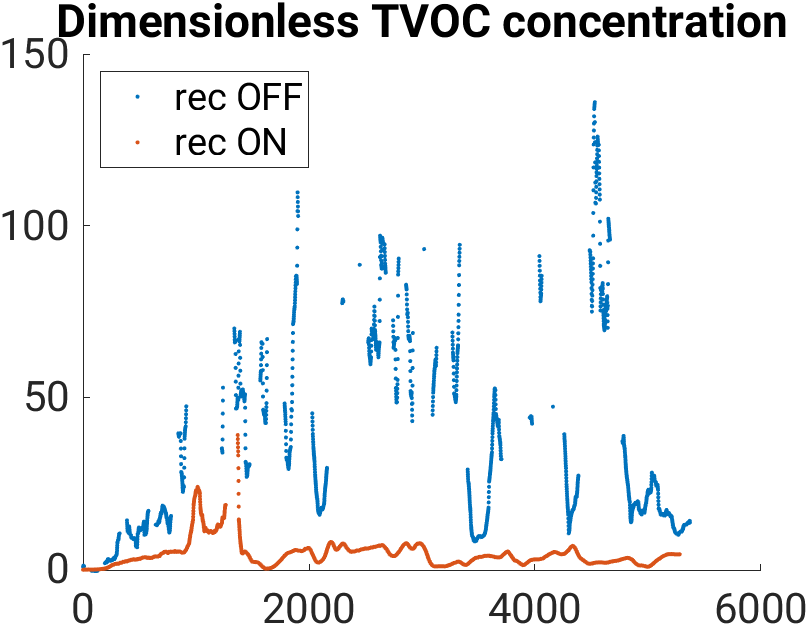

Supplement: Supplementary file 1 [file sensors-22-00543-s001.zip › sensors-1450505 -supp -for pub-final/img/TVOC_adim.png]

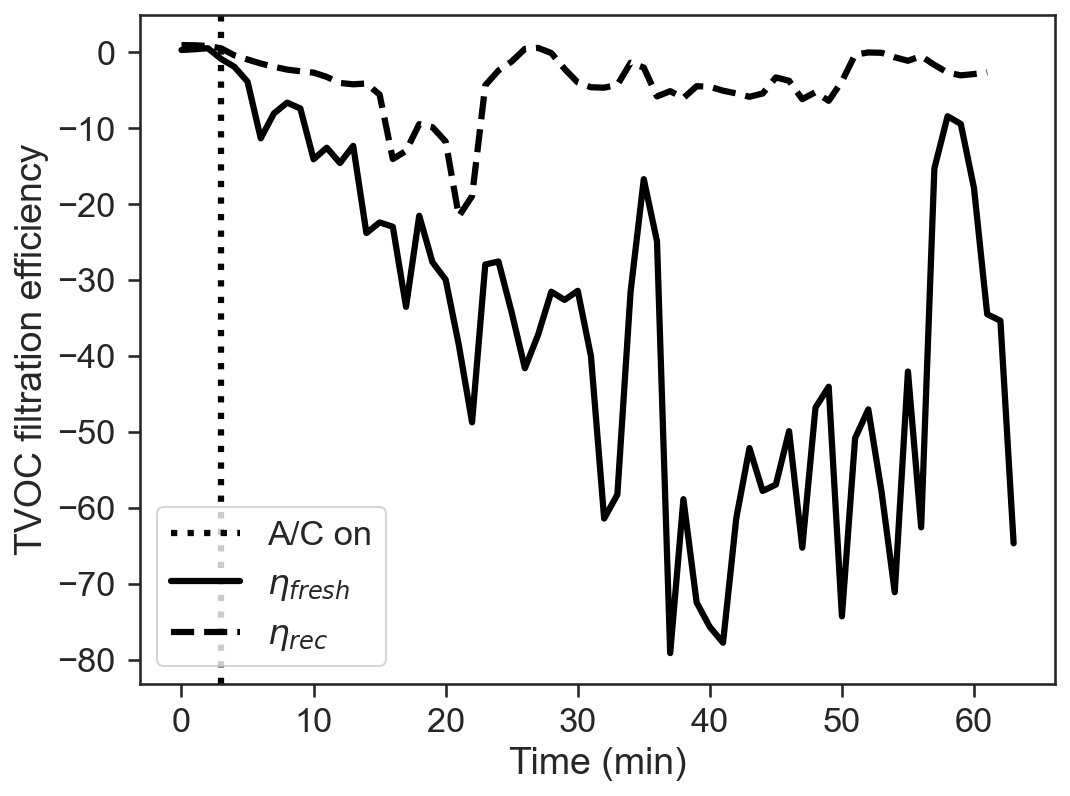

Supplement: Supplementary file 1 [file sensors-22-00543-s001.zip › sensors-1450505 -supp -for pub-final/img/TVOC_eta_ONOFF.png]

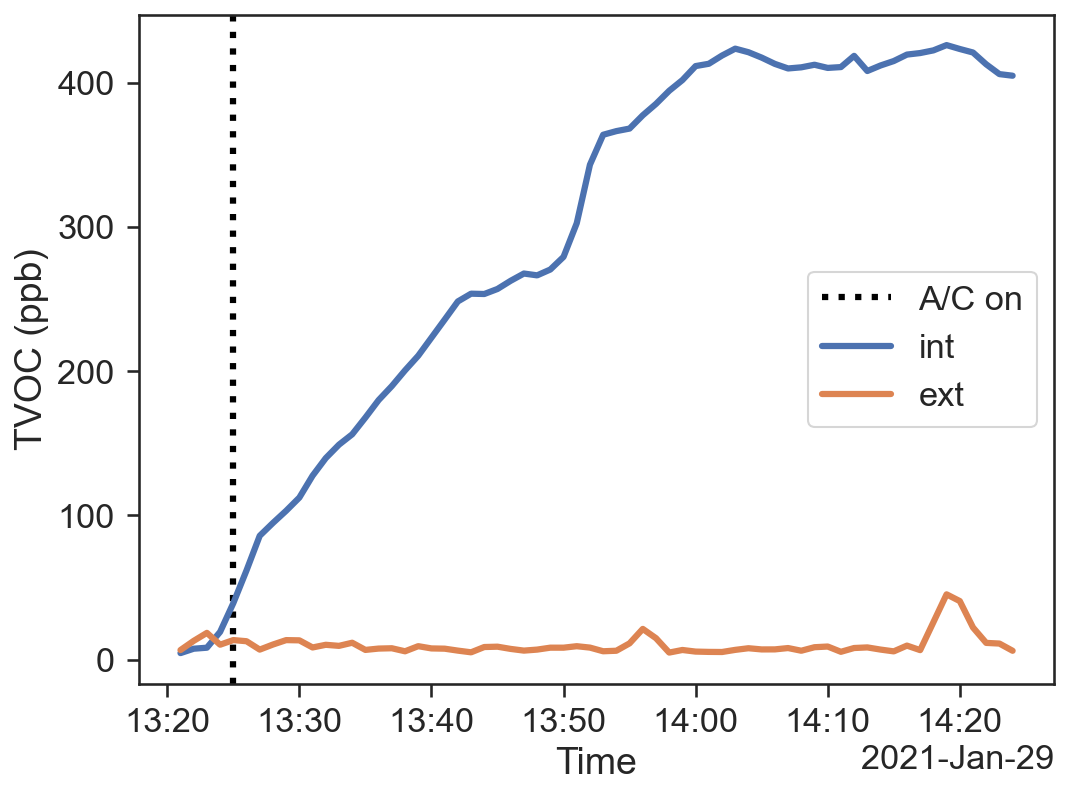

Supplement: Supplementary file 1 [file sensors-22-00543-s001.zip › sensors-1450505 -supp -for pub-final/img/TVOC_recOFF.png]

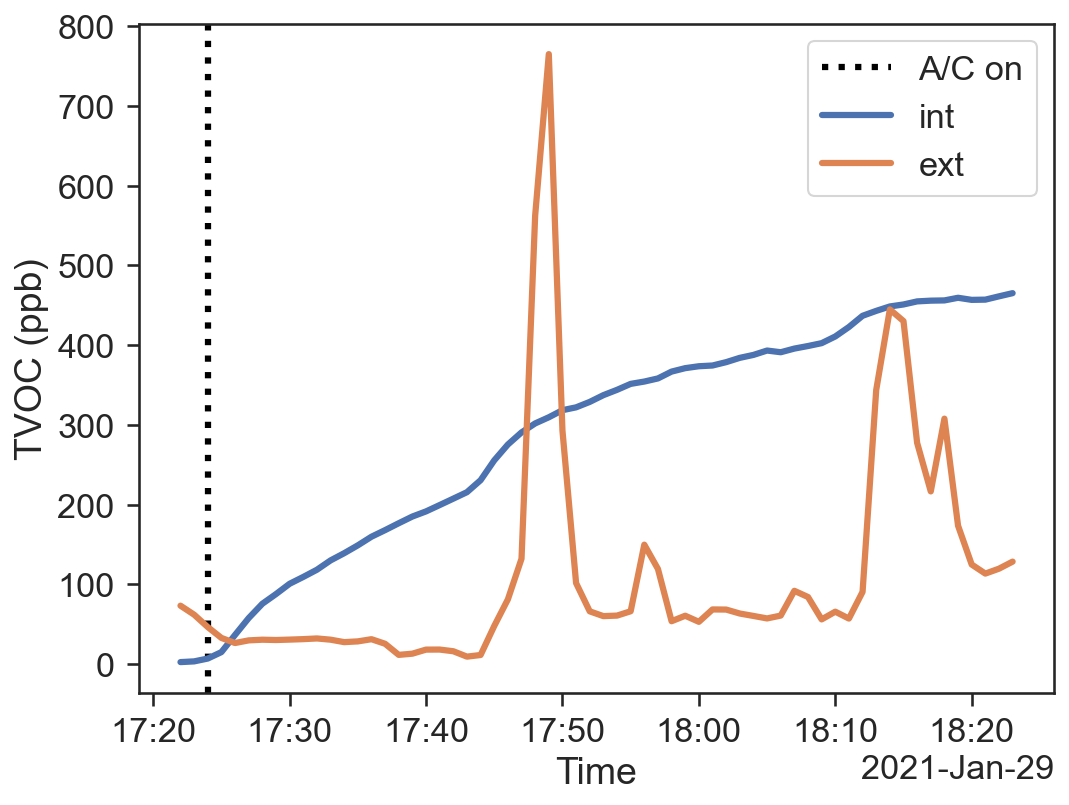

Supplement: Supplementary file 1 [file sensors-22-00543-s001.zip › sensors-1450505 -supp -for pub-final/img/TVOC_recON.png]

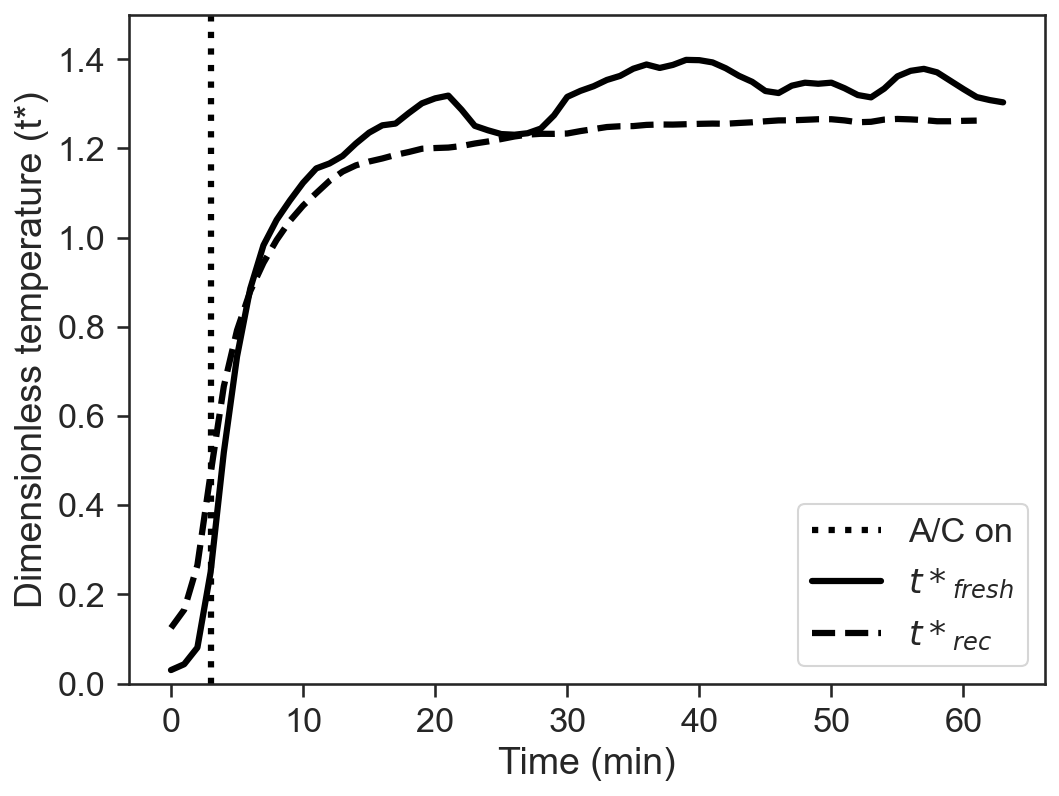

Supplement: Supplementary file 1 [file sensors-22-00543-s001.zip › sensors-1450505 -supp -for pub-final/img/T_adim.png]

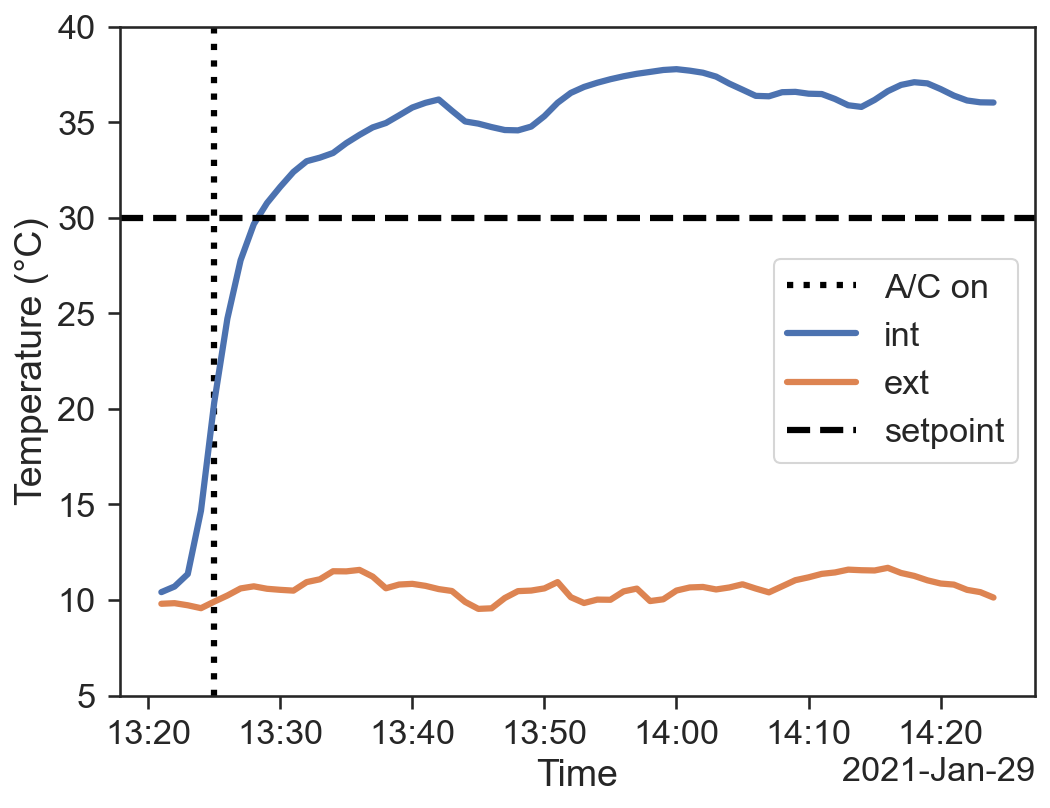

Supplement: Supplementary file 1 [file sensors-22-00543-s001.zip › sensors-1450505 -supp -for pub-final/img/T_recOFF.png]

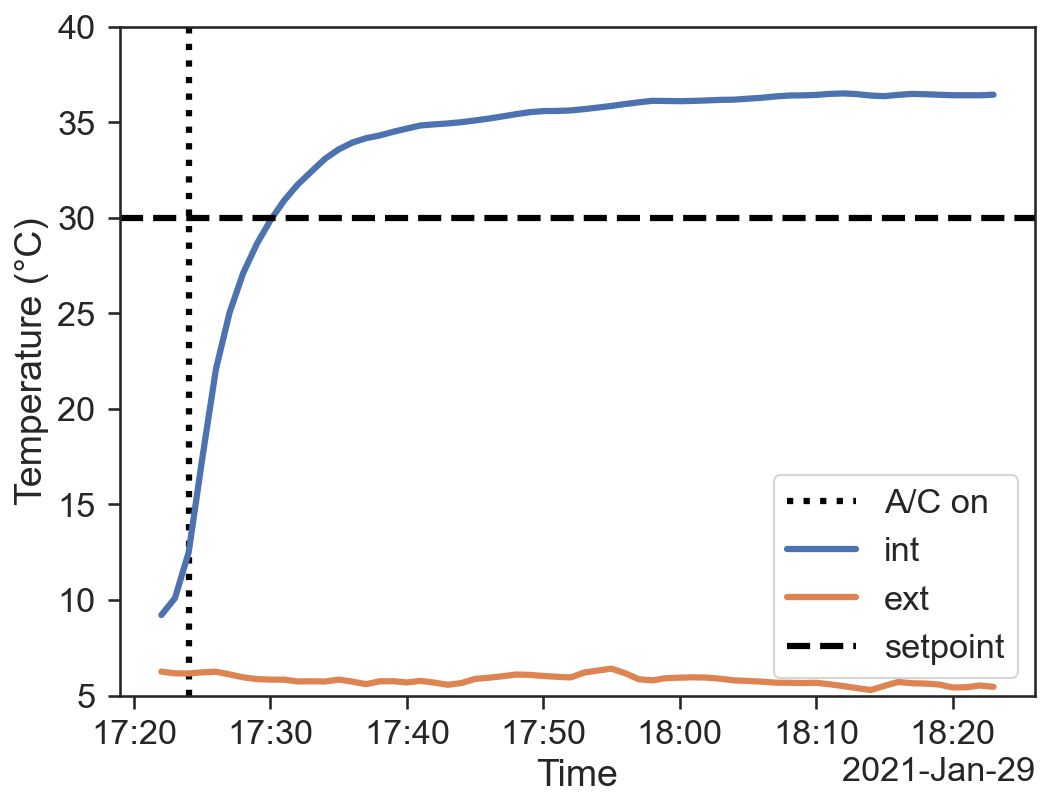

Supplement: Supplementary file 1 [file sensors-22-00543-s001.zip › sensors-1450505 -supp -for pub-final/img/T_recON.png]
